# Targeted β‐Glucan‐Veiled Oral Apremilast Nanotherapy Modulates Key Dysbiosis‐Associated Gut Microbiota and Alleviates Ulcerative Colitis‐Associated Anxiety, Depression, and Neuropsychiatric Behaviors

**DOI:** 10.1002/advs.76566

**Published:** 2026-07-20

**Authors:** Chandrashekhar Jori, Ahmed Shaney Rehman, Taruna Lamba, Anas Ahmad, Jattin Kumar, Aneesh Ali, Ajesh Joshi, Ashraf Ali, Suhel Parvez, Javed N. Agrewala, Rehan Khan

**Affiliations:** ^1^ Chemical Biology Unit Institute of Nano Science and Technology Mohali Punjab India; ^2^ Department of Toxicology School of Chemical and Life Sciences Jamia Hamdard New Delhi India; ^3^ Immunology Laboratory Department of Biomedical Engineering Indian Institute of Technology Ropar, Rupnagar Punjab India; ^4^ Department of Biotechnology Punjab University Chandigarh India

**Keywords:** apremilast, gut–brain axis, gut microbiome homeostasis, inflammatory bowel disease, neurocognitive impairment, psychiatric disorders, targeted drug delivery

## Abstract

Oral nanomedicines that modulate gut microbiota and gut–brain interactions are crucial for effectively treating inflammatory bowel disease (IBD) and associated psychiatric disorders, such as anxiety and depression. However, the underlying causes of psychiatric disorders in patients with IBD remain unclear, and effective treatment strategies have yet to be established. Herein, we developed oral gastroprotective βG@Apr‐WPG NMs (β‐glucan armored apremilast encapsulating tryptophan‐poly(lactic‐co‐glycolic acid)‐glutathione nanomicelles). This system effectively treats IBD and associated anxiety/depression by modulating the microbiota–gut–brain axis. βG@Apr‐WPG NMs demonstrated gastroprotection enhances retention, and enables sustained release within the inflamed colon, enhances drug solubility, and inflammation‐responsive apremilast release significantly improving oral therapeutic efficacy. Oral βG@Apr‐WPG NMs administration outperformed free apremilast by restoring gut barrier integrity, reducing histopathological damage, and modulating microbial dysbiosis. Systemic inflammation and neuroinflammation were markedly suppressed. Notably, the βG@Apr‐WPG NMs ameliorated anxiety‐ and depression‐like symptoms, as well as cognitive deficits in colitis‐induced mice, highlighting its therapeutic impact beyond the gut. Addressing reduced efficacy of conventional therapies, this multifunctional βG@Apr‐WPG NMs platform offers safe, simple, and highly efficient therapeutic strategy integrating targets intestinal inflammation, microbiota–gut–brain axis modulation, in the pathogenesis of IBD with comorbid neuropsychiatric disorders with confirmed safety.

## Graphical abstract

This study introduces β‐glucan‐shielded apremilast laden nanomicelles that withstand gastrointestinal degradation, selectively accumulate in inflamed colon tissue, and modulate the microbiota–gut–brain axis. The formulation restores barrier integrity, reduces systemic and neuroinflammation, and improves anxiety‐ and depression‐like behaviors in colitis models. It presents a safe, targeted nanotherapeutic strategy for ulcerative colitis and associated neuropsychiatric disorders.

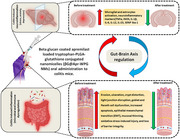

## 1 Introduction

Oral drug delivery has long been the preferred route for treating inflammatory diseases, including inflammatory bowel disease (IBD) and associated neuroinflammation (anxiety, depressive like behavior and other psychiatric disorders) [1, 2]. However, conventional oral formulations often suffer from poor bioavailability, rapid degradation in the gastrointestinal (GI) tract, and systemic side effects, limiting their therapeutic efficacy [3, 4]. These challenges are particularly evident in targeting gut microbiome dysbiosis and brain neuroinflammation through the gut–brain axis (GBA) [5, 6]. Patients with IBD exhibit a significantly higher prevalence of psychiatric disorders, particularly anxiety and depression, compared to the general population. Depression affects approximately 25% of IBD patients, versus 3.4% in the general population [7]. These comorbidities complicate disease management, increase relapse risk, and reduce quality of life [8]. Although the underlying mechanisms remain unclear and treatment options are limited, emerging studies on the effects of antidepressants and IBD‐specific therapies have yielded inconclusive results. This highlights the need for further investigation into the pathophysiology and treatment of psychiatric comorbidities in IBD. Despite numerous attempts to modulate the GBA, current therapeutic strategies, including small molecules and biologics, fail to provide sustained relief due to the poor drug stability, lack of selective targeting, inability to sustain therapeutic concentrations at inflamed sites, degradation by digestive enzymes, and poor permeability across the intestinal and blood–brain barriers [9, 10].

Gut microbiome dysbiosis plays a crucial role in the pathogenesis of cognitive neuroinflammatory disorders (anxiety, depressive like behavior and other psychiatric disorders) [6]. An imbalance in beneficial and pathogenic microbial populations disrupts gut homeostasis, leading to increased intestinal permeability and systemic inflammation [11, 12, 13]. The activation of microglia and the release of pro‐inflammatory cytokines, including tumor necrosis factor‐alpha (TNF‐α) and interleukin‐6 (IL‐6), exacerbate neuronal damage and cognitive decline [14, 15, 16]. While efforts to restore gut microbiota homeostasis through probiotics, dietary interventions, and anti‐inflammatory drugs have shown promise, they often lack targeted and sustained therapeutic action [17, 18, 19, 20].

The gut–brain axis has been widely explored as a potential therapeutic target, yet current treatments have largely failed to achieve meaningful clinical outcomes [21, 22]. Oral formulations often degrade before reaching their intended targets, while systemic administration leads to off‐target effects and toxicity [23, 24]. Moreover, the complex interplay between gut microbiota, intestinal inflammation, and cognitive neuroinflammation demands a multi‐pronged approach that addresses both local and systemic inflammatory cascades. Current ulcerative colitis (UC) therapies suffer from systemic toxicity, poor bioavailability, and non‐specific drug release, failing to address gut–brain axis dysregulation a key driver of cognitive neuroinflammatory comorbidities [25, 26]. Existing nanoformulations lack dual functionality: gastroprotection and inflammation‐responsive drug release.

Apremilast, a phosphodiesterase‐4 (PDE4) inhibitor, has shown anti‐inflammatory effects and is being explored as a potential therapeutic option for immune‐mediated conditions, including IBD [27]. Apremilast, recently shown in a 12‐week phase II trial (NCT02289417) to be safe and effective for UC, with lower serious side effects (0% in 30 mg, 1.8% in 40 mg) vs. placebo (3.4%), offers a promising therapeutic candidate [28, 29, 30]. However, its potential in modulating gut–brain axis pathways via targeted delivery remains unexplored.

To overcome these challenges, we developed an oral administrable nanoformulation, βG@Apr‐WPG NMs, designed to enhance the stability, bioavailability, and targeted delivery of apremilast. This nanomicellar system encapsulates apremilast within a polymeric core conjugated with tryptophan‐poly(lactic‐co‐glycolic acid) (PLGA)‐glutathione (WPG), further coated with β‐glucan (βG). The nanomicellar system incorporates: β‐Glucan (βG) provides gastroprotection, prevents premature drug degradation, and modulates the immune response to promote mucosal healing [31, 32, 33]. Tryptophan (W): Supports gut microbiota balance by enhancing beneficial bacterial populations and producing anti‐inflammatory metabolites [34, 35, 36, 37]. PLGA (P): Ensures controlled and sustained drug release at inflamed sites, improving local efficacy while minimizing systemic side effects [38, 39]. Glutathione (G): Acts as an antioxidant, reducing oxidative stress and preserving gut epithelial integrity [40]. The βG@Apr‐WPG NMs not only shield the drug from premature degradation in the GI tract but also facilitate site‐specific release in inflamed gut regions. Moreover, the inclusion of tryptophan and glutathione helps modulate gut microbiota composition, reducing oxidative stress, and restoring immune homeostasis.

Our study demonstrates that βG@Apr‐WPG NMs effectively address the limitations of conventional oral therapies by: (1) enhancing drug stability and bioavailability through nanomicellar encapsulation. (2) Providing inflammation‐responsive apremilast release at the site of gut inflammation. (3) Restoring gut microbiota balance, promoting beneficial bacterial populations, and reducing pathogenic overgrowth. (4) Suppressing systemic and neuroinflammatory responses (inhibiting activation of the central microglia) via the gut–brain axis.

Through in vitro and in vivo studies, we establish the superior efficacy of oral βG@Apr‐WPG NMs in mitigating gut and brain inflammation in the IBD and IBD‐associated cognitive impairment (anxiety, depressive like behavior and other psychiatric disorders), surpassing the therapeutic performance of free apremilast and existing treatment strategies. This nanoformulation represents a promising advancement in precision nanomedicine for IBD and IBD related psychological comorbidities, offering a multifaceted approach to managing gut dysbiosis, cognitive neuroinflammation, and their systemic consequences.

## 2 Experimental Section

### 2.1 Chemicals

The following materials were used in this study: Apremilast (CAS 608141‐41‐9, MW‐ 460.5 g/mol), beta glucan (CAS 9012‐72‐0), and tryptophan (CAS 73‐22‐3), glutathione (CAS 70‐18‐8) were procured from TCI Chemicals Pvt. Ltd. Toluidine blue (TB), Alcian blue (AB), and the Griess Reagent stains were procured from Alfa Aesar. Specific targeting primary antibodies TLR4, STING, IRF3, cAMP response element‐binding protein (CREB), PDE4, NF‐κB, NLRP3, cluster of differentiation (CD8), AMPK, MUC2, Occludin, tight junction protein (TJP), Claudin, and ZO1 were procured from Cloud Clone. Primary antibody Iba1 (Cat. No. E4O4W, Cell Signaling Technology), secondary antibody, goat anti‐rabbit Alexa Fluor 555 (Invitrogen, Scotland, UK), potassium chloride (KCl), sodium chloride (NaCl), potassium dihydrogen phosphate (KH_2_PO_4_), disodium hydrogen phosphate (Na_2_HPO_4_), paraformaldehyde, and supplementary reagents were procured from Himedia Laboratories. All chemicals employed in this research were of analytical grade and employed as received, without additional modification or dilution. All solutions were prepared using distilled water.

### 2.2 Methodology

#### 2.2.1 Synthesis of Tryptophan (Trp)‐PLGA‐GSH (WPG) Conjugate

The Trp‐PLGA‐glutathione (GSH) (WPG) conjugate was synthesized through a two‐step reaction. First, PLGA (2 g, 57 µmol) was activated in anhydrous N,N‐Dimethylformamide (DMF) using 1‐Ethyl‐3‐(3‐dimethylaminopropyl)carbodiimide (EDC) (13.29 mg, 85.6 µmol) and N‐Hydroxysuccinimide (NHS) (9.8 mg, 85.6 µmol) under nitrogen at low temperature, followed by the addition of glutathione (17.56 mg, 57 µmol) and stirring for 24 h at room temperature. The crude PLGA‐GSH (PG) product was precipitated with diethyl ether, collected by centrifugation, and dried. In the second step, tryptophan (23 mg, 113 µmol) was activated in DMF using EDC (24 mg, 160 µmol), followed by addition of the PG conjugate (4 g, 113 µmol) and 4‐(Dimethylamino)pyridine (DMAP) (20 mg, 113 µmol). The reaction mixture was stirred for 24 h, and the WPG conjugate was obtained by precipitation in diethyl ether and characterized by ^1^H NMR.

#### 2.2.2 Fabrication of βG@Apr‐WPG NMs

The βG@Apr‐WPG NMs (β‐glucan armored apremilast encapsulating tryptophan‐PLGA‐glutathione nanomicelles) were synthesized using the thin‐film hydration method, followed by layer‐by‐layer (LbL) assembly for β‐glucan coating. Initially, 100 mg of WPG conjugate and 30 mg of apremilast were dissolved in an organic solvent. A thin film was formed by solvent evaporation and subsequently rehydrated with distilled water under continuous sonication. To facilitate β‐glucan coating, a 1:1 (w/v) ratio of Apr‐WPG NMs was subjected to LbL assembly with β‐glucan (βG), resulting in the formation of βG@Apr‐WPG NMs. For the preparation of blank (without apremilast) nanomicelles (βG@B‐WPG NMs), the same protocol was followed.

### 2.3 Characterization

The structural, morphological, and physicochemical properties of βG@Apr‐WPG NMs were comprehensively characterized using advanced analytical techniques. Fourier transform infrared spectroscopy (FTIR) was employed to analyze the chemical composition and functional groups of free apremilast, β‐glucan, PLGA, tryptophan, glutathione, and βG@Apr‐WPG NMs, with spectra recorded over the range of 400–4000 cm^−^^1^. Dynamic light scattering (DLS) and zeta potential measurements were conducted using a Zetasizer Nano ZSP to determine the hydrodynamic diameter, size distribution, polydispersity index (PDI), and surface charge of the nanoparticles. Transmission electron microscopy (TEM) and field emission scanning electron microscopy (FESEM) were utilized to examine the morphology and nanostructure of βG@Apr‐WPG NMs, with samples prepared on carbon‐coated copper grids and silicon wafers, respectively. Additionally, ultraviolet–visible (UV–Vis) spectroscopy was used to construct a calibration curve for apremilast and assess the drug‐loading efficiency and encapsulation capacity of the nanoparticles. Together, these techniques provided a detailed understanding of the structural integrity, stability, and functional properties of βG@Apr‐WPG NMs, ensuring their suitability for therapeutic applications.

### 2.4 Evaluation of βg@Apr‐WPG NMs in a DSS‐Induced Colitis Model: Therapeutic Efficacy and Safety Assessment

All experimental procedures were conducted in accordance with the guidelines approved by the Institutional Animal Ethics Committee (IAEC) of Jamia Hamdard, New Delhi, India (CPCSEA Registration No. 173/GO/ReBi/2000/CPCSEA, Project No. 2258). Female BALB/c mice (20–25 g, total 48) were procured from the Central Animal House Facility, Jamia Hamdard, and used to establish the experimental colitis model and treatment. The animals were housed under standard laboratory conditions, maintained on a 12‐h light/dark cycle at a controlled temperature and humidity. Mice had ad libitum access to a standard pellet diet (M/s. Ashirwad Industries Pvt. Ltd., Mohali, Punjab, India) and filtered water (RIOS, Merck Millipore, Billerica, MA, USA).

The therapeutic efficacy of βG@Apr‐WPG NMs was evaluated in dextran sulfate sodium (DSS)‐induced colitis model using Swiss albino mice. A total of 48 mice were randomly divided into six groups (*n* = 8 per group), as detailed below:

- Group 1 (Control, Healthy): Mice were fed a standard diet without any treatment and served as the healthy control group.
- Group 2 (DSS Colitis model): Mice received 2.5% w/v DSS in drinking water ad libitum for 10 days to induce colitis.
- Group 3 (DSS + βG@Apr‐WPG NMs, treatment group): Colitis‐induced mice were treated with βG@Apr‐WPG NMs (equivalent to 50 mg/kg body weight of apremilast) orally for 10 alternate days post‐induction to evaluate therapeutic efficacy.
- Group 4 (DSS + Free apremilast): Colitis‐induced mice received free apremilast (50 mg/kg body weight) orally for 10 alternate days to assess the efficacy of the unencapsulated apremilast.
- Group 5 (DSS + βG@B‐WPG NMs, blank nanomicelles): Colitis‐induced mice were treated with blank nanomicelles (without apremilast) orally for 10 alternate days to evaluate the role of the nanocarrier alone.
- Group 6 (Control + βG@B‐WPG NMs, safety group): Healthy mice received blank nanomicelles orally for 10 alternate days to assess toxicity in vital organs (heart, spleen, kidneys, lungs, and liver).

#### 2.4.1 Colitis Induction

Colitis was induced by administering 2.5% w/v DSS in drinking water ad libitum for 10 days. Colonic inflammation was confirmed by evaluating physical activity, stool consistency, and rectal bleeding [41].

#### 2.4.2 Evaluation of Colitis and Physical Parameters

Humane endpoints were monitored based on signs of colitis, including bloody diarrhea, loose stool, and reduced physical activity. Disease progression was assessed using the Disease Activity Index (DAI), which incorporates body weight loss, fecal consistency, and occult blood. Following euthanasia, colon length was measured as an indicator of inflammation severity, with shorter lengths reflecting more severe colitis. These parameters were used to evaluate the therapeutic efficacy of βG@Apr‐WPG NMs and free apremilast.

#### 2.4.3 Histological Analysis

Colon and vital organ tissues were fixed in 10% neutral buffered formalin for 24 h, embedded in paraffin, and sectioned into 5 µm slices. Alcian blue‐neutral red, Hematoxylin and eosin (H&E), and High Iron Diamine and Alcian Blue (HID‐AB) staining was performed to evaluate histological damage, including mucosal epithelial integrity, interstitial edema, crypt depletion, and inflammatory cell infiltration.

#### 2.4.4 Quantification of Serum Inflammatory Cytokines and Oxidative Stress Markers in Colitis Mice

Following various treatments, serum samples were collected from mice and appropriately diluted for analysis. The concentrations of key inflammatory markers, including IL‐6, IL‐1β, TNF‐α, transforming growth factor‐beta (TGF‐β), myeloperoxidase (MPO), and nitrite, were measured using enzyme‐linked immunosorbent assay (ELISA) kits (eBioscience). This analysis provided critical insights into the anti‐inflammatory efficacy of βG@Apr‐WPG NMs in modulating systemic cytokine levels and oxidative stress markers in the DSS‐induced colitis model.

#### 2.4.5 Inflammatory Cytokine Analysis in the Colon Samples (ELISA)

Colon segments were homogenized in phosphate buffer containing 0.05% Tween 20, 10 mM EDTA, and 20 UI aprotinin A. The homogenate was centrifuged at 3000 × g for 10 min, and the supernatant was collected and stored at −80°C. Cytokine levels (TNF‐α, IL‐6, IFN‐γ, IL‐12, IL‐10, and TGF‐β) were measured using ELISA kits (BD Biosciences) following the manufacturer's protocol. Primary antibodies (2 µg/mL) were coated onto plates and incubated overnight at 4°C. Unsaturated sites were blocked with 1% bovine serum albumin, and standards/supernatants were added and incubated for 1 h. Secondary antibodies (1 µg/mL) and horseradish peroxidase (HRP)‐avidin were added, followed by 3,3',5,5'‐Tetramethylbenzidine substrate for color development. The reaction was stopped with 0.14 M H_2_SO_4_, and absorbance was measured at 450 nm. Total protein in each sample was quantified using a bicinchoninic acid kit for accurate normalization.

#### 2.4.6 Fecal Sample Collection and DNA Extraction

Fecal samples were aseptically collected, placed on ice immediately, and stored at −80°C for subsequent analysis. DNA extraction was performed using the QIAamp Power Fecal Pro DNA Kit. Briefly, 250 mg of fecal sample was homogenized with 800 µL of Solution CD1 in a Power Bead Pro Tube and vortexed for 10 min. The mixture was centrifuged at 15 000 × g for 1 min, and the supernatant was transferred to a clean 2 mL microcentrifuge tube. Solutions CD2 (200 µL) and CD3 (600 µL) were added sequentially, followed by vortexing and centrifugation. The lysate was loaded onto an MB Spin Column, washed with 500 µL of ethanolamine solution and 500 µL of Solution C5, and eluted with Solution C6. DNA amount was quantified via NanoDrop spectrophotometer which further stored for downstream applications.

#### 2.4.7 Bacterial Genus‐Specific Quantitative PCR

For bacterial quantification, 400 ng of extracted genomic DNA was used for real‐time PCR with genus‐specific primers (Table 1) and SYBR Green PCR Master Mix (Applied Biosystems). Reactions were performed on a StepOne Plus PCR system under the following cycling conditions: 95°C for 10 min, followed by 40 cycles of 95°C for 30 s and 60°C for 1 min. Bacterial abundance was expressed as fold change, normalized to universal bacterial DNA as an internal control, enabling precise quantification of specific bacterial genera in fecal and colon samples.

**TABLE 1:** Primer sequences used for amplification of bacterial genus‐specific quantitative PCR.

Target genus	Primers
*Shigella*	Forward‐ 5′‐ACCATGCTCGCAGAGAAACT‐3′ Reverse‐ 5′‐TACGCTTCAGTACAGCATGC‐3′
*Akkermansia muciniphila*	Forward: 5′‐CAGCACGTGAAGGTGGGGAC‐3′ Reverse‐ 5′‐CCTTGCGGTTGGCTTCAGAT‐3′
*Escherichia coli (E. coli)*	Forward: 5′‐CATGCCGCGTGTATGAAGAA‐3′ Reverse: 5′‐CGGGTAACGTCAATGAGCAAA‐3′
*Firmicutes*	Forward: 5′‐GCGTGAGTGAAGAAGT‐3′ Reverse: 5′‐CTACGCTCCCTTTACAC‐3′
Proteobacteria	Forward: 5′‐TGGTGTAGGGGTAAAATCCG‐3′ Reverse: 5′‐AGGTAAGGTTCTTCGYGTATC‐3′

#### 2.4.8 Immunofluorescence Staining for Microglial Activation

Mice were anesthetized and perfused transcardially with 4% paraformaldehyde (PFA). Brains were post‐fixed in 4% PFA for 24 h, embedded in paraffin, and sectioned coronally (4–5 µm). Sections were deparaffinized, rehydrated, and subjected to antigen retrieval in 10 mM sodium citrate buffer (pH 6.0) via microwave heating. After permeabilization with 0.1% Triton X‐100 and blocking with 5% normal goat serum at 37°C for 1 h, sections were incubated overnight at 4 °C with Iba1/Allograft Inflammatory Factor‐1 (AIF‐1) primary antibody (1:50, CST). Following phosphate‐buffered saline (PBS) washes, Alexa Fluor 555‐conjugated secondary antibody (1:500, Invitrogen) was applied for 1 h at room temperature. Sections were counterstained with 4',6‐Diamidino‐2‐Phenylindole, mounted in antifade medium, and imaged at 20× magnification using a Zeiss fluorescence microscope. Microglial activation was assessed in the ipsilateral cerebral cortex.

### 2.5 Behavioral Assessment of Motor Function and Coordination in Colitis Mice

#### 2.5.1 Beam Walk Test

Motor coordination and balance were assessed using a beam walk test adapted from standard protocols. Mice performed five trials on a 160 cm‐long, 2.5 cm wide beam, with the mean of three consistent trials used for analysis. Each trial ended when the mouse traversed 1 m or after 60 s. A scoring system (0–5) was applied, awarding one point for every 12 s the mouse remained on the beam. Fall frequency and traversal time were recorded as measures of vestibulomotor function to evaluate the impact of colitis‐induced motor coordination and the therapeutic effect of βG@Apr‐WPG NMs.

#### 2.5.2 Grip Strength Test

Muscular strength was evaluated in DSS‐induced colitis mice and other experimental groups using a grip strength apparatus consisting of a 50 cm long metal wire tightly stretched between two vertical supports, positioned 40 cm above a level surface. The test was scored on a six‐point scale: 0 = falling, 1 = holding the wire with both forelimbs, 2 = attempting to climb the wire, 3 = holding the wire with both forelimbs and one or both hindlimbs, 4 = holding the wire with both forelimbs and the tail wrapped around it and 5 = escaping. This test assessed the neuromuscular function and overall physical strength of the mice, providing insights into the systemic effects of colitis and the therapeutic efficacy of βG@Apr‐WPG NMs.

### 2.6 Preparation of Single‐Cell Suspensions from Gut‐Associated Lymphoid Tissue (GALT)

To investigate gut immune responses and evaluate the immunomodulatory effects of βG@Apr‐WPG NMs in a murine model of colitis, immune cells were isolated from gut‐associated lymphoid tissues (GALT), specifically targeting lamina propria (LP) and intraepithelial lymphocytes (IELs) from the colon. Following euthanasia, mouse colons were carefully excised and thoroughly rinsed with Hank's Balanced Salt Solution (HBSS) to remove residual debris. Any attached adipose tissue was gently trimmed away. To clear the lumen of fecal content, colons were flushed with Dulbecco's Modified Eagle Medium (DMEM) supplemented with 10% fetal bovine serum (FBS). The cleaned tissues were then cut into small fragments and incubated in IEL isolation buffer (PBS containing 0.1 mM EDTA) at 37°C to facilitate the release of intraepithelial lymphocytes. Post‐incubation, the tissue mixture was filtered through a cell strainer and vigorously vortexed to further dislodge IELs. The filtrate, containing IELs, was collected for downstream processing. The remaining tissue fragments were subjected to enzymatic digestion using collagenase in DMEM supplemented with 10% FBS to liberate lamina propria lymphocytes. This mixture was incubated under agitation and subsequently passed through a 70 µm cell strainer to obtain a single‐cell suspension. Cells from both IEL and LP fractions were washed, stained with fluorochrome‐conjugated antibodies specific to cell surface markers, and fixed for flow cytometric analysis. This procedure enabled the identification, quantification, and functional assessment of distinct immune cell populations within the colon.

### 2.7 Flow Cytometric Profiling of Brain Immune Cells

To characterize brain immune cells via flow cytometry, mice brains were harvested 24 h post‐injury following anesthesia and perfusion. Hemispheres were separated, dissociated enzymatically and mechanically using a modified Neural Tissue Dissociation Kit protocol (Miltenyi Biotec). Myelin was removed via 30% Percoll density gradient. Microglia were enriched using CD11b/c magnetic sorting. Cells were stained with a multicolor antibody panel targeting M1/M2 markers, including viability dye and secondary antibodies, and analyzed using an LSRII flow cytometer. Compensation and dimensionality reduction (tSNE) were performed using standard software (Kaluza and FlowJo).

### 2.8 Statistical Analysis

Data were analyzed using GraphPad Prism (GraphPad Software, San Diego, CA, USA). For comparisons between two groups, an unpaired Student's *t*‐test was applied. When evaluating differences across multiple groups, a One‐way ANOVA was performed, followed by Tukey's multiple‐comparison testpost hoc test for pairwise comparisons. Statistical significance was set at #*p* < 0.05, ##*p* < 0.01, ###*p* < 0.001, ####*p* < 0.0001 vs. Control group; *p < 0.05, **p < 0.01, ***p < 0.001, ****p < 0.0001 vs. DSS group.

## 3 Results and Discussion

### 3.1 Synthesis and Characterization of Beta Glucan Glazed WPG Conjugate Nanomicelles

**SCHEME 1:**
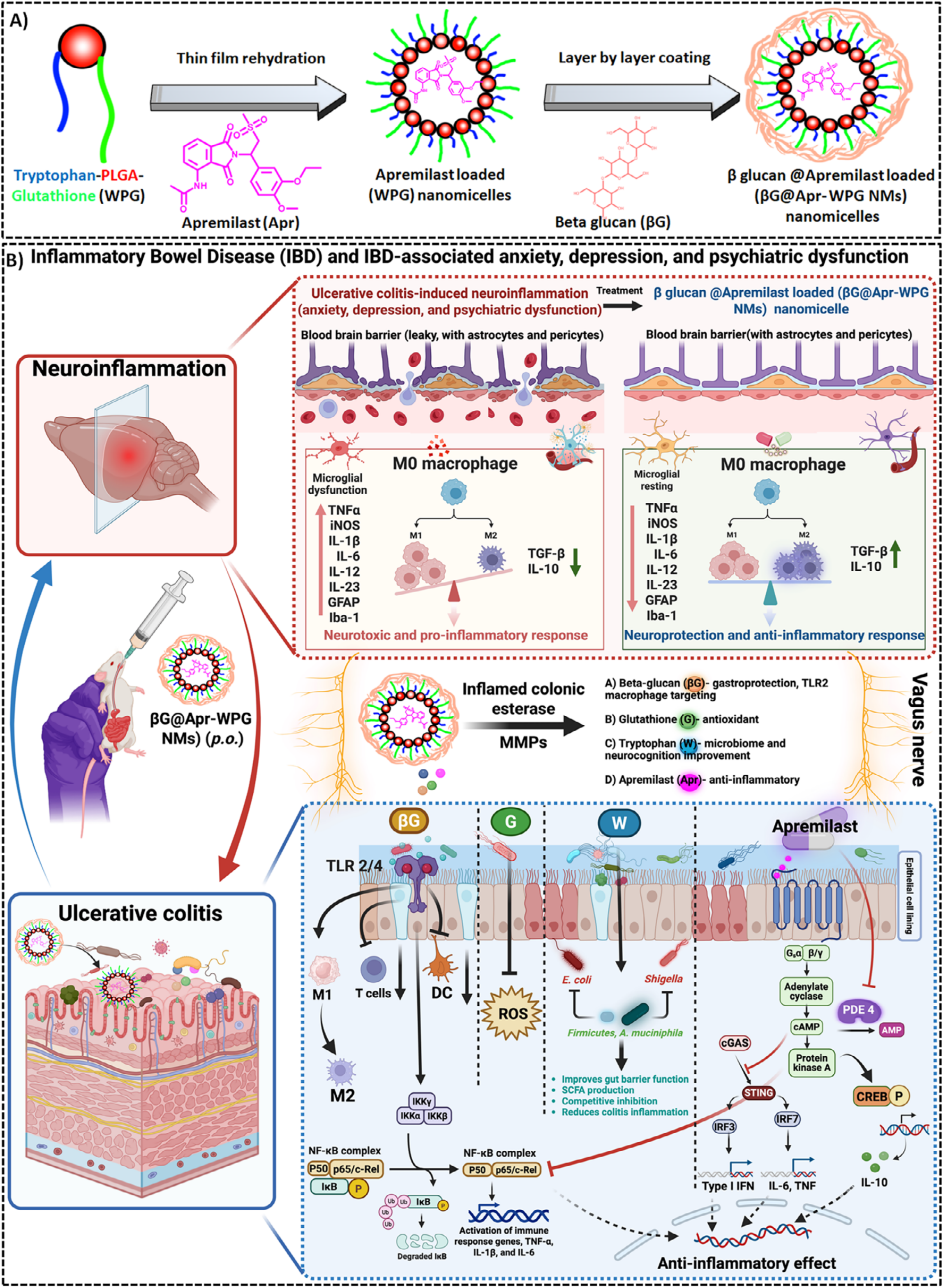
Schematic illustration of the mechanism of inflammation‐responsive targeted βG@Apr‐WPG NMs for enhanced therapeutic modulation of the gut microbiome‐brain axis in ulcerative colitis. (A) Schematic representation for the fabrication of beta‐glucan (βG) armored apremilast (Apr) encapsulated Tryptophan‐PLGA‐Glutathione (WPG) conjugated nanomicelles (NMs) (βG@Apr‐WPG NMs) nanomedicines. (B) βG@Apr‐WPG NMs were administered orally via gavage, effectively traversing the harsh gastric environment due to the protective beta‐glucan (βG) coating. Upon reaching the intestinal tract, the βG layer gradually degraded, enabling the Apr‐WPG NMs to accumulate at the inflamed colon site through mechanisms, such as electrostatic interactions and βG mediated adhesion. At the target site, βG@Apr‐WPG NMs modulated the inflammatory microenvironment by suppressing inflammatory responses and restoring the balance of intestinal microbiota. Additionally, the NMs alleviated neuroinflammation in the brains of IBD mice via the gut–brain axis, as evidenced by reduced activation of astrocytes, microglia, and other neuroinflammatory cells. This mitigation of brain inflammation subsequently led to improved cognitive function in the treated mice. (B) Created with Biorender.com.

**FIGURE 1:**
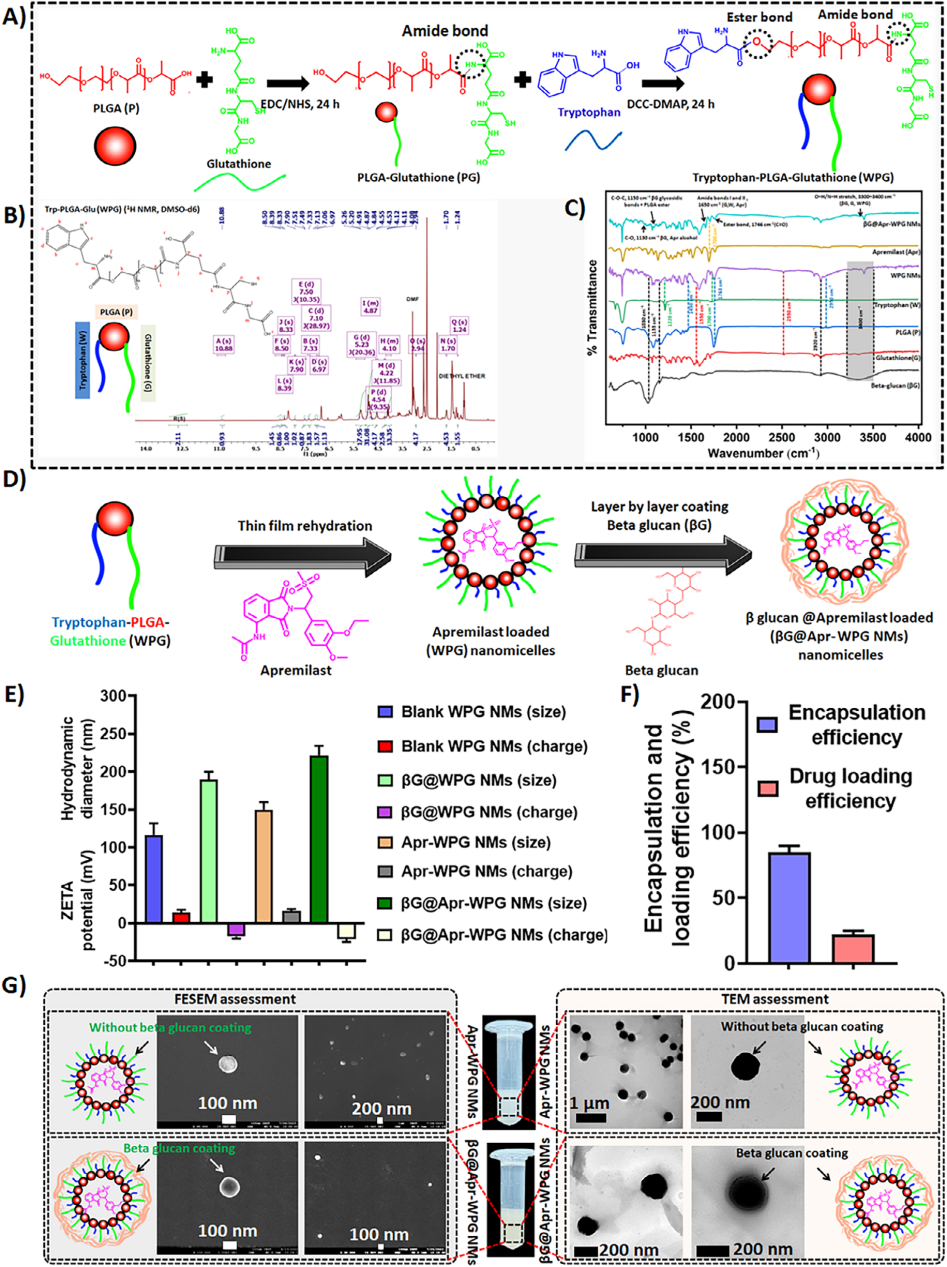
Fabrication and characterization of βG@Apr‐WPG NMs: (A) Schematic representation of the synthesis of the Tryptophan‐PLGA‐Glutathione (WPG) conjugate. (B) ^1^H NMR spectra confirming the structure of the WPG conjugate. (C) FTIR analysis for functional group characterization Beta‐glucan (βG), glutathione (G), PLGA (P), tryptophan (W), WPG NMs, apremilast (Apr), βG@Apr‐WPG NMs. (D) Fabrication of βG@Apr‐WPG NMs using thin film rehydration (Apr‐WPG NMs) and layer‐by‐layer coating (βG@Apr‐WPG NMs). (E) Evaluation of hydrodynamic diameter (size) and zeta potential (charge) for WPG NMs, βG@WPG NMs, Apr‐WPG NMs, and βG@Apr‐WPG NMs. (F) Apremilast encapsulation and loading efficiency in βG@Apr‐WPG NMs (*n* = 3, mean ± SD). (G) Morphological analysis via FESEM and TEM, upper panel showing uncoated (Apr‐WPG NMs) and lower panel showing beta‐glucan‐coated (βG@Apr‐WPG NMs) structures.

Schematic illustration of how βG@Apr‐WPG NMs (β‐glucan armored apremilast encapsulating tryptophan‐PLGA‐glutathione nanomicelles) target inflammation and improve therapy in the IBD, gut microbiome‐brain axis, and IBD‐associated psychiatric dysfunction. (Scheme 1 and Scheme S1). The WPG conjugate were fabricated by conjugating PLGA (P) (50:50) to glutathione (G) by EDC/NHS reaction followed by tryptophan (W) esterification (DCC/DMAP) to previous PG conjugate to form WPG conjugate as shown in Figure 1A. The ^1^ H NMR, and HRMS spectra of WPG conjugate are shown in Figure 1B and Figures S1–S4. The results of FTIR and XRD measurements were showed in Figure 1C and Figures S5–S6. The WPG conjugate nanomicelles (WPG NMs) were synthesize by the self‐assembly process (thin film layer) and glazed with beta‐glucan (Figure 1D).

DLS was initially used to assess the size and PDI of blank WPG NMs and apremilast loaded WPG NMs. DLS measurement analysis exhibits 116 nm, and 190 nm of hydrodynamic diameter (particle size) for WPG NMs, and βG@WPG NMs (beta‐glucan coated WPG NMs, respectively. Upon incorporating apremilast into WPG NMs (Apr‐WPG NMs) and subsequently coating those with beta‐glucan (βG@Apr‐WPG NMs), the mean hydrodynamic diameter exhibited increments to 150 and 221 nm, respectively (Figure 1E). Moreover, WPG NMs, βG@WPG NMs, Apr‐WPG NMs, and βG@Apr‐WPG NMs display monodispersity, as shown by PDI values of 0.3, 0.2, 0.298, and 0.239 which suggest the lack of agglomeration formation. Zeta potential analysis showed a change from positive (Apr‐WPG NMs, +14 mV) to anionic by coating with gastro resistance beta‐glucan (βG), i.e., βG@Apr‐WPG NMs was confirmed by a zeta potential measurement of ‐21 mV (Figure 1E). Storage stability of βG@Apr‐WPG nanomicelles was assessed over 60 days by monitoring changes in hydrodynamic size (nm), zeta potential (mV), and encapsulation efficiency under defined storage conditions (Table S1). The UV–vis spectroscopy measurement and calibration curve (Figure S7) showed that the βG@Apr‐WPG NMs, apremilast encapsulation efficiency (EE) was around 85% and the drug loading (DL) was approximately 22% into the NMs, which is excellent for apremilast delivery (Figure 1F). The variations in hydrodynamic size, zeta potential, and encapsulation efficiency were systematically monitored to assess the formulation's stability. The βG@Apr‐WPG NMs exhibited consistent stability over the 60 day storage period, with only minimal fluctuations observed in these critical parameters, indicating their suitability for long‐term application.

Given that the βG@Apr‐WPG NMs are intended for delivering medication regionally on the intestinal barrier, the NMs must be stable for sufficient time to reach the inflamed mucosa and be disintegrated by the inflammatory cascades near the inflamed colon. We evaluated the morphologies of Apr‐WPG NMs (without beta glucan coated) and βG@Apr‐WPG NMs (beta glucan coated) using a FESEM and transmission electron microscope (TEM). FESEM and TEM analysis results confirmed that Apr‐WPG NMs (Figure 1G, upper panels) and βG@Apr‐WPG NMs (Figure 1G, lower panels) had spherical, monodisperse, agglomeration‐free nanomicelles with a size of 200–230 nm. The TEM micrographs clearly show the coating of beta glucan (Figure 1G, right panel), which are employed to fabricate the βG@Apr‐WPG NMs.

The viscoelastic behavior of βG@Apr‐WPG nanomicelles (NMs) was characterized via amplitude and frequency sweep rheology. In the amplitude sweep (0.01%–100% strain), the storage modulus (*G*′) exhibited a plateau at ∼30 Pa across the linear viscoelastic region (LVR), significantly exceeding the loss modulus (*G*″), which remained near ∼5 Pa. Beyond ∼10% strain, both *G*′ and *G*″ declined, indicating network structure breakdown under mechanical deformation (Figure S8A). In the frequency sweep (0.1–100 rad/s), *G*′ remained consistently dominant over *G*″ across the tested range, with *G*′ ∼35 Pa and *G*″ ∼6 Pa at 10 rad/s, signifying a predominantly elastic, solid‐like response. The weak frequency dependence of the moduli supports the presence of a physically cross‐linked nanostructure with stable internal dynamics. These rheological signatures underscore the mechanical robustness and viscoelastic stability of the βG@Apr‐WPG NMs, essential for their functional performance in physiological or dynamic environments (Figure S8B).

### 3.2 Biocompatible, Digestive Enzyme‐Resistant, In Vitro Drug Release Kinetics, Cytocompatibility and Anti‐Inflammatory Studies

**FIGURE 2:**
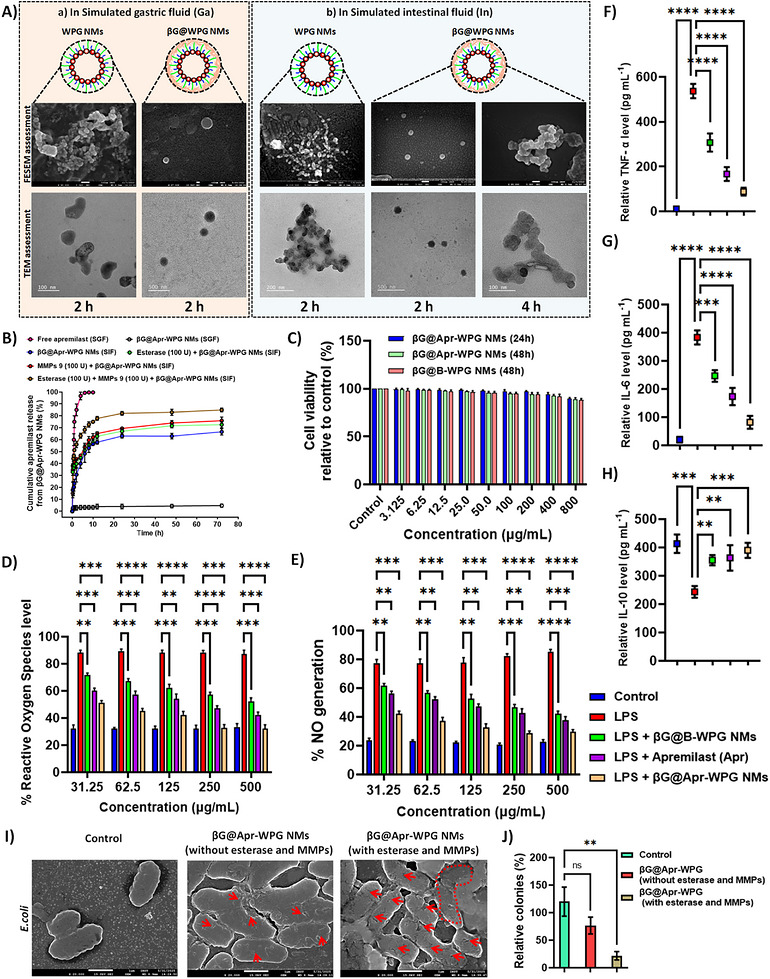
In vitro evaluation of digestive enzyme resistance, apremilast release kinetics, biocompatibility, and anti‐inflammatory activity of the βG@Apr‐WPG NMs. (A) FESEM and TEM microscopic images of WPG NMs and βG@WPG NMs following incubation at room temperature simulated in (a) gastric fluid (Ga) at pH 1.2 for 2 h, and (b) intestinal fluid (In) at pH 6.8 for 2 h (WPG NMs) and 2 and 4 h (βG@WPG NMs). (B) Release kinetics of apremilast from βG@Apr‐WPG NMs in simulated gastric fluid (SGF) and simulated intestinal fluid (SIF) under varying enzymatic conditions: without enzymes, with esterase (100 U), with MMP‐9 (100 U), and with a combination of esterase (100 U) and MMP‐9 (100 U). (C) Cell viability assessment following 24 h incubation with βG@Apr‐WPG NMs and 48 h incubation with βG@Apr‐WPG NMs and βG@WPG NMs at concentrations ranging from 0 to 800 µg mL^−1^. (D,E) Evaluation of ROS scavenging properties and anti‐inflammatory activity of βG@WPG NMs by quantifying the percentage reduction in reactive oxygen species (ROS) and nitrate levels in LPS‐stimulated RAW 264.7 macrophage cells. (F–H) Enzyme‐linked immunosorbent assay (ELISA) results demonstrating the extracellular expression of (F) TNF‐α, (G) IL‐6, and (H) IL‐10. (I) FE‐SEM micrographs illustrating E. coli morphology after treatment. Red arrowheads indicate sites of cell membrane damage. Untreated control cells exhibited intact membranes, whereas cells exposed to βG@Apr‐WPG NMs with and without esterase show pronounced membrane disruption. (J) Quantitative analysis of colony formation of *E*. *coli*. (Data are expressed as mean ± SD; statistical significance is denoted as *p < 0.05, **p < 0.01, ***p < 0.001, and ****p < 0.0001).

We then investigated at the impact of the inflammatory environment on the gastrointestinal (GI) tract, which includes gastric acid and intestinal fluid, as well as the physiological procedure of peristalsis. To evaluate the pH‐responsive disintegrating properties, we incubate the samples in 1.2 pH simulated gastric fluid (Ga) and then in 6.8 pH (PBS). We have used FESEM and TEM photomicrographs to assess the structural alterations. Following a 2 h incubation of WPG NMs at the Ga, observable modifications in their structure and integrity were evident. Conversely, βG@WPG NMs, post‐coating, exhibited no discernible changes in morphology, maintaining a stable spherical shape and integrity in the Ga (Figure 2A(a)). On the other hand, in the In (at pH 6.8), WPG NMs began to lose their form after just 2 h, while βG@WPG NMs started to swell and disintegrate the β‐glucan layer in 2 h. The βG@WPG NMs slowly started to lose their shape and integrity after 4 h of incubation in In, which would result in the cargo release (Figure 2A(b)). These findings indicated the gastroprotective efficacy of βG@WPG NMs and cargo release in the specific inflamed colon vicinity, which is crucial for oral nanoformulation [41].

When treating IBD, a controlled release of drugs is preferred as it offers an advantage in reaching the appropriate concentration needed for targeted action and sustaining it for an extended amount of time within the intestine or colon. βG@Apr‐WPG NMs were utilized in vitro apremilast release experiments under simulated gastric fluid (SGF) and simulated intestinal fluid (SIF) environment. In SGF, free apremilast demonstrated rapid release within 2 h. In βG@Apr‐WPG NMs, the apremilast was enclosed in a WPG NMs core that may not disintegrate quickly in an acidic environment. This resisted release observed in βG@Apr‐WPG NMs (SIF) can be attributed to the encapsulation of apremilast within the nanomicellar core and showed gastroprotective characteristics of βG@Apr‐WPG NMs due to beta glucan coating. In βG@Apr‐WPG NMs, to check inflammatory enzyme (esterase [42] and matrix metalloproteinases 9 (MMP‐9 [43]), 100 U, (the concentration relevant to in vivo condition) responsive apremilast drug release kinetic profiles in the SIF demonstrated that in all samples, βG@Apr‐WPG NMs (only SIF) without any enzyme demonstrated the ∼60% apremilast release, βG@Apr‐WPG NMs (SIF + 100 U esterase enzyme) which showed approximate 70% apremilast release. βG@Apr‐WPG NMs (SIF + 100 U MMPs enzyme) which showed approximate 72% apremilast release, but in the presence of both inflammatory environment enzyme, i.e., βG@Apr‐WPG NMs (SIF + 100 U esterase + 100 U MMPs enzyme) showed enhanced apremilast release up to 80% after 72 h with sustained manner. Enzyme‐responsive βG@Apr‐WPG nanomicelles enable controlled, concentration‐dependent apremilast release in the presence of esterase and MMP‐9 found in the inflamed colon (Figure S9). It showed promising gastro‐resistance and inflammation responsive sustained apremilast drug release through βG@Apr‐WPG NMs. It again confirms the gastroprotective behavior of βG@Apr‐WPG NMs. There were notable variations in the apremilast release behavior at inflamed colonic pH 6.8 [44], with a slow release continuing for 4 h, as demonstrated in Figure 2B. The βG@Apr‐WPG NMs remained noncytotoxic in the concentration range of 0–800 µg/mL (Figure 2C). βG@B‐WPG NMs (without Apr) and βG@Apr‐WPG NMs were screened for cytocompatibility against HCT‐116 (human colorectal carcinoma cells) at 24‐ and 48‐h intervals so as to substantiate that the material was not affecting the normal cells. Based on the non‐significant change in cell viability, we noticed that βG@B‐WPG NMs nearly 400 µg/mL dosages were not damaging, i.e., biocompatible to HCT‐116 cells and had negligible effects.

Using lipopolysaccharide (LPS), an well‐established inducer of inflammatory response trigger via TLR‐4 receptor activation, murine macrophage cell line (RAW264.7) cells underwent stimulation to analyze the anti‐inflammatory potential of naïve Apr only, βG@B‐WPG NMs (without Apr) and βG@Apr‐WPG NMs. After subjecting cell lines to a 1 µg/mL dosage of the LPS, different doses of naïve Apr, βG@B‐WPG NMs, and βG@Apr‐WPG NMs were added to inflammatory response cells. Notably, treatment with βG@Apr‐WPG NMs showed a dose‐dependent decrease in nitrite levels significantly decreased than in the group treated with free Apr and βG@B‐WPG NMs (Figure 2E). This reduction was dose‐dependent, indicating potential enhancement of Apr therapeutic efficacy upon encapsulation in βG@Apr‐WPG NMs. Furthermore, similar results were found with the ROS production estimated through dichlorofluorescin diacetate (DCFDA) assay (Figure 2D). The anti‐inflammatory potential of βG@Apr‐WPG NMs in vitro was determined by assessing cytokine levels in LPS activated RAW264.7 macrophage cells. The findings indicated that βG@Apr‐WPG NMs considerably reduced the expression of TNF‐α and interleukin‐6 (IL‐6) (Figure 2F,G). Additionally, βG@Apr‐WPG NMs showed the strongest anti‐inflammatory activity, which was ascribed to the combination of the ROS‐scavenging of βG@B‐WPG NMs and free Apr. Furthermore, the relative expression levels of the genes linked to M2 macrophages, i.e., IL‐10 were evaluated. The results showed that βG@Apr‐WPG NMs considerably enhanced the production of IL‐10 (Figure 2H), indicating that βG@Apr‐WPG NMs exerted anti‐inflammatory effects by suppressing ROS, NO, TNF‐α, IL‐6 levels, and M1 macrophage polarization and stimulation of M2 macrophage activity (IL‐10). To gain further insight into the direct delivery of the neuroprotective effects of βG@Apr‐WPG NMs in the blood–brain barrier (BBB), an in vitro BBB co‐culture model was set up using bEnd.3 endothelial cells and U87 cells. The TEER values were significantly lower for LPS stimulation, reflecting BBB disruption, but TEER values were preserved with the use of βG@Apr‐WPG NMs relative to the free apremilast and βG@B‐WPG NMs (Figure S10A,B). In addition, ex vivo ICG‐nanomicelles imaging showed a negligible fluorescent signal in the brain, while high levels of signaling were detected in the colon (Figure S10C). The results indicate that βG@Apr‐WPG NMs are not readily translocated across the BBB and that their beneficial effects on neuroinflammation are more likely to be through modulation of the intestinal inflammation and microbiota–gut–brain axis signaling. FESEM analysis revealed pronounced morphological disruption in E. coli treated with βG@Apr‐WPG NMs both with and without esterase compared to the control (Figure 2I,J). These results demonstrate the enhanced antibacterial and bactericidal efficacy of βG@Apr‐WPG NMs in the presence of inflammatory enzymes against E. coli.

### 3.3 Therapeutic Effect of βg@Apr‐WPG NMs Against IBD Model

#### 3.3.1 ΒG@Apr‐WPG NMs for Effective Treatment of Colitis

**FIGURE 3:**
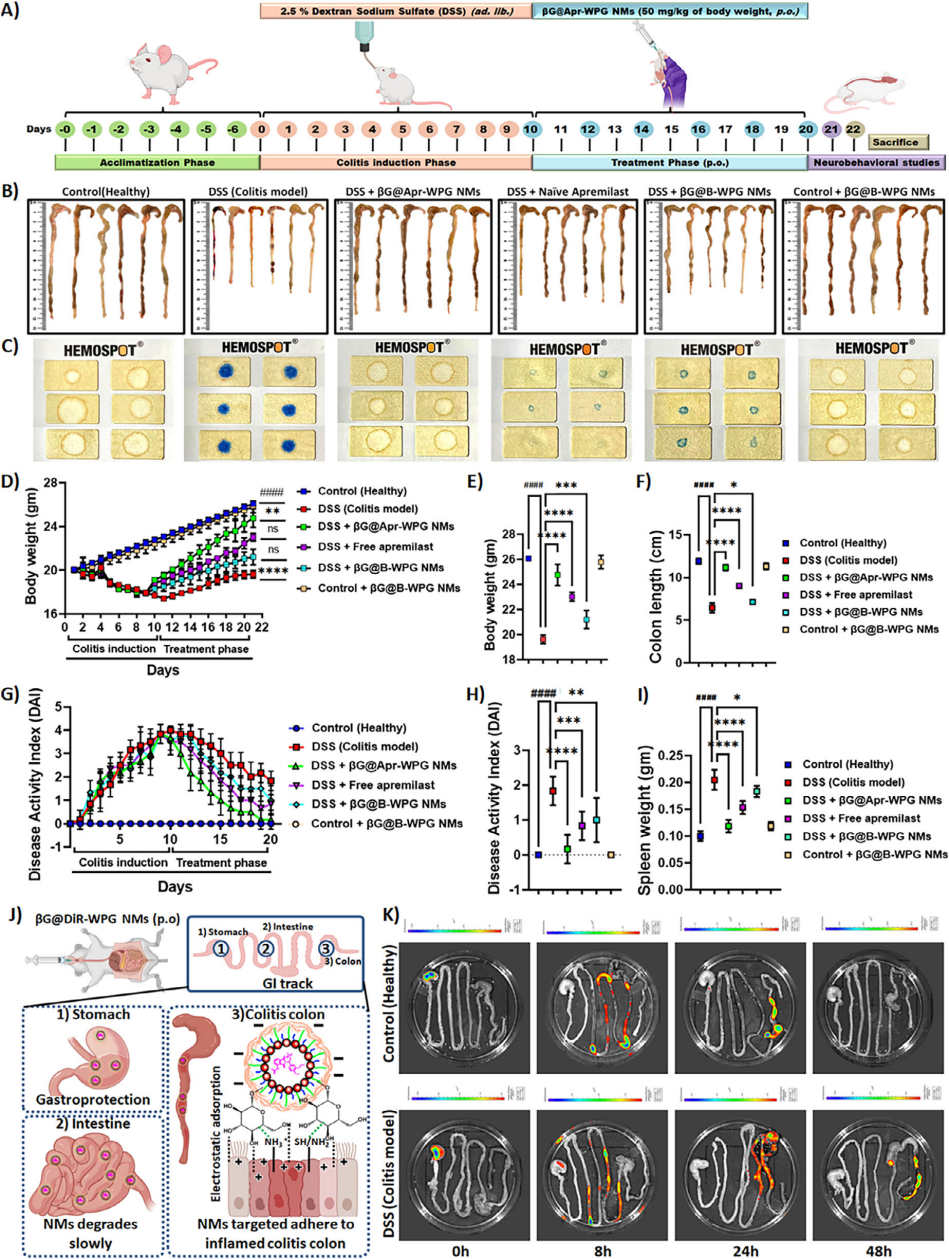
In vivo assessment of βG@Apr‐WPG NMs for therapeutic efficacy in inflammatory bowel disease. (A) Schematic representation of the experimental design for βG@Apr‐WPG NMs treatment in a DSS‐induced colitis model. Colitis was induced in mice by administering 2.5% DSS in drinking water for 10 days. Oral treatments were initiated on the 10th day and administered alternately until the 21st day when neurobehavioral assessments were conducted. On the 22nd day, mice were euthanized via cervical dislocation, and colon tissues were collected for further analysis. Abbreviations: ad lib. (ad libitum), p.o. (per os/oral) (*n* = 8). Created with Biorender.com. (B,F) Colon lengths were measured and analyzed (*n* = 6). (C) Evaluation of fecal occult blood test (FOBT) strip results of stool samples (*n* = 6). (D,E) Daily body weight changes in mice were recorded throughout the treatment period and on the 21st day prior to sacrifice, with detailed analysis (*n* = 6). (G,H) Disease activity index (DAI) was monitored throughout the treatment period and on the 21st day before sacrifice, followed by detailed analysis (*n* = 8). (I) Spleen weights in mice were measured and analyzed (*n* = 8). Long‐term retention of β‐glucan coated nanomedicine in the inflamed colon. (J) Schematic illustration depicting the oral administration pathway of βG@Apr‐WPG nanomicelles (NMs), highlighting their resistance to enzymatic and acidic degradation within the gastrointestinal tract. The β‐glucan outer layer enhances mucosal adhesion and enables targeted accumulation and prolonged retention within the inflamed colonic tissue. (K) In vivo fluorescence imaging showing the retention of βG@DiR‐WPG nanomicelles in healthy and DSS‐induced colitis mice (2.5% DSS for 10 days) at 0, 8, 24, and 48 h post‐intragastric administration. Prolonged retention up to 48 h in the inflamed colon was observed. (Data are expressed as mean ± SD; statistical significance is denoted as #*p* < 0.05, ##*p* < 0.01, ###*p* < 0.001, ####*p* < 0.0001 vs. Control group; *p < 0.05, **p < 0.01, ***p < 0.001, ****p < 0.0001 vs. DSS group, ns—non‐significant).

To further investigate the therapeutic potential of βG@Apr‐WPG NMs in DSS‐treated mice (2.5%, w/v), the oral gavage of βG@Apr‐WPG NMs was carried out (Figure 3A). βG@Apr‐WPG NMs‐mediated therapy potently protected mice against DSS‐induced shortening of colon, the relief of blood in the stool (interestingly, treatment with βG@Apr‐WPG NMs improved activity (Video S1) and considerably lowered occult blood than naïve Apr, and βG@B‐WPG NMs treated group (Figure S11). Using the FOBT kit, fecal occult blood was evaluated; in untreated colitis mice, the presence of blood in the fecal samples were indicated by a blue stain. The treatment of βG@Apr‐WPG NMs diminished occult blood and loss of body weight (Figure 3B–F). The disease activity index (DAI score, the measure of physical activity, stool consistency, and rectal bleeding) notably decreased after the treatment with βG@Apr‐WPG NMs, whereas the group treated with naïve apremilast, and without apremilast βG@B‐WPG NMs generally showed higher DAI score and spleen weight (Figure 3G–I). The typical symptoms of colitis, including the loss of body weight and the decrease of colon length were relieved in the naïve apremilast, and without apremilast βG@B‐WPG NMs group. Bloating is a prominent symptom of UC [45]. Using a 2.5% DSS‐induced colitis mouse model, significant abdominal bloating and colon damage were observed. Treatment with βG@Apr‐WPG nanomicelles effectively reduced bloating and restored colon morphology (Figure S12), highlighting their therapeutic potential for UC. To enhance oral delivery and site‐specific retention, βG@Apr‐WPG NMs were engineered to resist gastrointestinal degradation and adhere to inflamed colonic tissue. Their spherical, mucoadhesive structure enables adaptation to varying GI conditions such as pH shifts, motility, and fluid content facilitating durable mucosal coating and sustained drug release. The β‐glucan outer shell protects the nanomicelles from enzymatic and acidic breakdown and promotes selective accumulation at inflammation sites through microbial‐triggered activation and mucosal adhesion.

To evaluate bioadhesive performance, DiR‐labeled βG@WPG NMs (βG@DiR‐WPG) were administered orally to healthy and DSS‐induced colitis mice. Ex vivo fluorescence imaging at 0, 8, 24, and 48 h post‐gavage revealed strong and prolonged signal retention in the inflamed colons of DSS‐treated mice, indicating effective mucosal binding and long‐term residence. In contrast, minimal fluorescence was detected in healthy controls after 24 h. This prolonged retention aligns with in vitro bioadhesion data and highlights the importance of surface engineering for targeted colonic delivery. The β‐glucan coating interacts with inflamed mucosa through hydrogen bonding, hydrophobic effects, and electrostatic interactions with positively charged proteins in the colonic environment, enabling precise localization and therapeutic efficacy in colitis treatment (Figure 3J,K).

#### 3.3.2 Histological Inflammation Mitigation by βg@Apr‐WPG NMs in Colitis Mice

**FIGURE 4:**
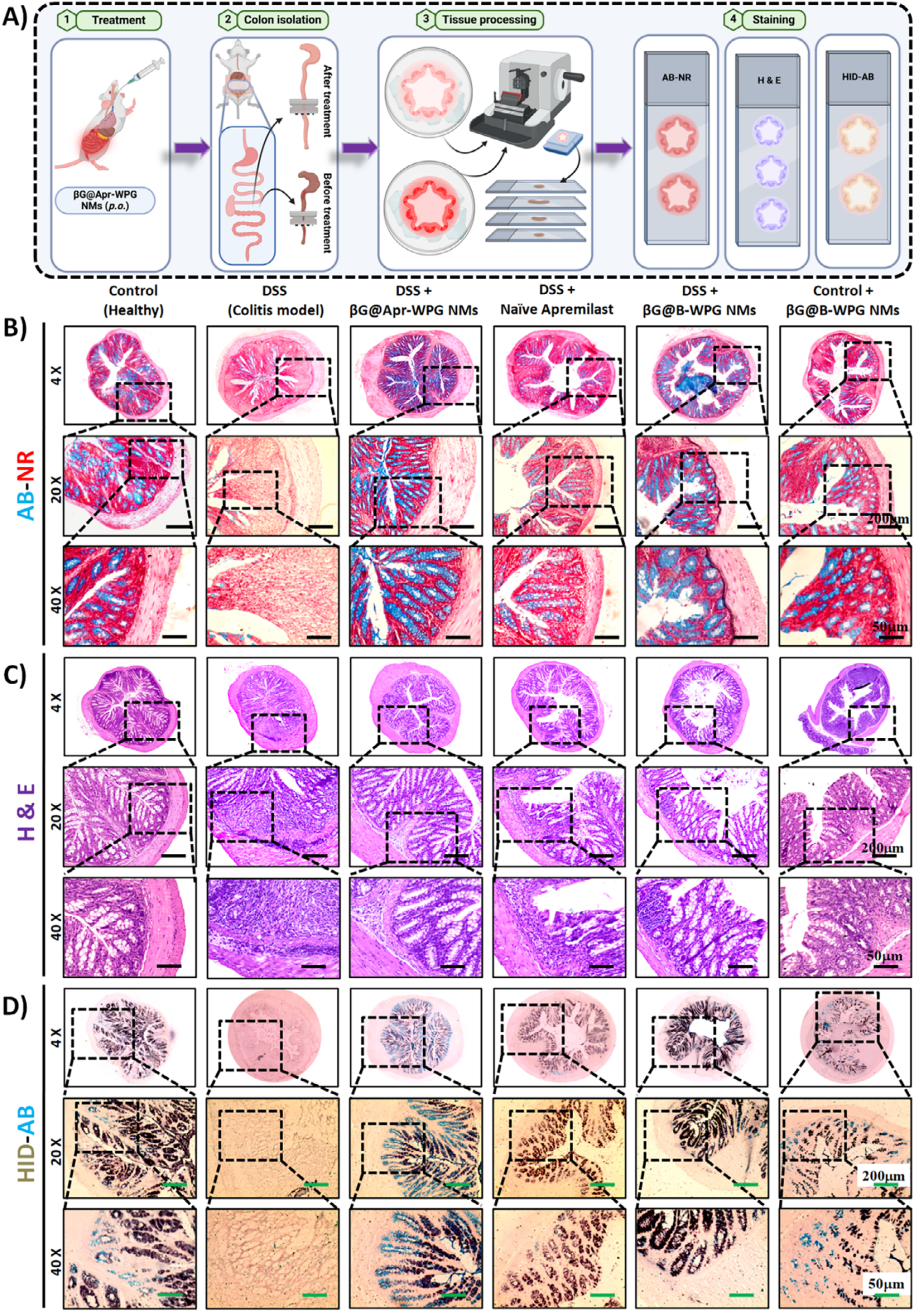
Histological analysis demonstrating inflammation mitigation in colitis mice treated with βG@Apr‐WPG NMs. (A) Schematic representation of colon isolation, highlighting the specific region (e.g., distal colon) primarily focused on histological inflammation analysis using various staining methods. Created with Biorender.com. (B) Alcian blue‐neutral red (AB‐NR) staining: Blue staining indicates a continuous mucin layer in the control (healthy) and βG@Apr‐WPG NMs groups. The DSS (colitis model) group showed loss of mucin from colon tissue. In the βG@B‐WPG NMs + DSS group (colitis mice), the mucin layer was significantly reduced than the βG@Apr‐WPG NMs groups. The naive apremilast group exhibited partial recovery of mucin content, though less pronounced compared to βG@B‐WPG NMs treatment. (C) Representative H&E‐stained micrographs: Control, βG@Apr‐WPG NMs, and naive apremilast groups displayed minimal histological inflammation. Untreated colitis mice and DSS + βG@B‐WPG NMs groups exhibited severe histological damage, including compromised mucosal epithelial integrity, partial erosion of the colonic epithelium and lamina propria, crypt ablation, and significant inflammatory cell infiltration. βG@Apr‐WPG NMs treatment in colitis mice reduced mucin breakdown and inflammation. (D) Representative High Iron Diamine and Alcian Blue (HID‐AB) staining micrographs: βG@Apr‐WPG NMs showed greater efficacy in sulfomucin induction compared to naive apremilast treatment. Scale bars: 200 µm (20X magnification) and 50 µm (40X magnification).

Goblet cells in the colon release a mucin that protects, and the devastation of these cells exacerbates the disease the state of colitis. In colitis group, populations of goblet cells were significantly reduced, evidenced by Alcian blue‐neutral red staining. The goblet cells preservation was enhanced by free apremilast therapy, while maintaining the mucin layer's integrity was disturbed. βG@Apr‐WPG NMs showed a continuous, undamaged mucin layer (blue staining), and more goblet cells, which recommended improved defense against damage to the colonic crypt and maintained stability of the intestinal epithelium (Figure 4B). Staining with hematoxylin and eosin (H&E) [46, 47] representative images revealed that the untreated colitis mice with βG@Apr‐WPG NMs had significantly less histological inflammation. Conversely, mice with untreated colitis (2.5% DSS), free apremilast, and βG@B‐WPG NMs showed histological damages, such as impaired integrity of the mucosal epithelial lining, crypt ablation, interstitial edema, significant infiltration of inflammatory cells, and partial erosion of the colonic epithelial layer or lamina propria. In the H&E stained colon sections, the colitic mice, and free apremilast, DSS + βG@B‐WPG NMs (without apremilast) group demonstrated pronounced morphological damage like epithelial disruption, crypt loss, and inflammatory cell infiltration compared to the other groups, particularly those treated with control, and βG@Apr‐WPG NMs groups. Indicating that the βG@B‐WPG NMs had lacked significant therapeutic effects than βG@Apr‐WPG NMs. These changes were indicative of severe colonic injury and inflammation caused by DSS‐induced colitis. In contrast, the βG@Apr‐WPG NMs (apremilast loaded nanomicelles) group showed marked histological improvements, including preserving crypt structure, reducing inflammatory cell infiltration, and restoring the epithelial lining. The control group exhibited normal colon morphology, while the free apremilast treated group demonstrated partial recovery with reduced histopathological damage. The differences observed under H&E staining highlighted the therapeutic role of apremilast in mitigating colonic damage and inflammation, reinforcing the importance of the apremilast loaded nanomicelles, i.e., βG@Apr‐WPG NMs in protecting colon morphology (Figure 4C). Goblet cells exert two different mucins: sialomucin and sulfomucin. Studies have shown that intestinal inflammation is linked with higher secretions of sialomucin, while functional mucosa mainly produces sulfomucin [48, 49]. Goblet cell‐produced sulfomucin (brown stain) and sialomucin (blue stain) were distinguished by High Iron Diamine and Alcian Blue (HID‐AB) stains. Higher levels of sulfomucin were found in control colons, but colitis tissue had a higher concentration of sialomucin. Mice in the free apremilast group excreted more sulfomucin, whereas βG@Apr‐WPG NMs showed a noteworthy and higher augmentation of sulfomucin in colitis mice. Sulfomucin levels were higher than sialomucin levels, primarily in control and βG@Apr‐WPG NMs groups. The study findings indicate that βG@Apr‐WPG NMs have shown more effectiveness in stimulating the synthesis of sulfomucin, highlighting its ability to protect the integrity of the intestinal barrier in colitis mice by enhancing healthy mucin (Figure 4D) and quantified as Figure S13. Colitis has been seen to have mast cell infiltration in the submucosal layers of the inflammatory colon. Histamine is released at the inflammation site due to mast cell activation driven on by this invasion [50]. Consequently, histamine actively mediates alterations caused by inflammation through cellular and humoral pathways. Mast cells invading the inflammatory colon, a hallmark of colitis, was shown by toluidine blue (TB) staining. Activation of mast cells occurred in the submucosal layer of the colon of colitis disease mice, however, they were missing at healthy and βG@B‐WPG NMs animals and βG@Apr‐WPG NMs (Figure S14). βG@Apr‐WPG NMs proficiently averted mast cell infiltration and alleviated inflammatory alterations in the colitis.

#### 3.3.3 Modulation of Inflammatory Pathways and Restoration of Gut Homeostasis by βG@Apr‐WPG NMs in Ulcerative Colitis

The pathogenesis of UC is closely linked to the dysregulation of inflammatory signaling pathways, such as TLR4/NF‐κB and STING/IRF3, which drive pro‐inflammatory cytokine production and disrupt epithelial integrity. Down regulation of tight junction proteins (e.g., Occludin, TJP, Claudin, ZO1) and mucin (MUC2) exacerbates mucosal barrier dysfunction, facilitating bacterial translocation and systemic inflammation. In this study, βG@Apr‐WPG NMs effectively targeted these pathways, suppressing pro‐inflammatory signaling and enhancing epithelial repair mechanisms. Notably, their therapeutic efficacy surpassed that of free apremilast and DSS + βG@B‐WPG NMs, as evidenced by a more pronounced reduction in inflammatory markers and greater restoration of barrier function biomarkers.

**FIGURE 5:**
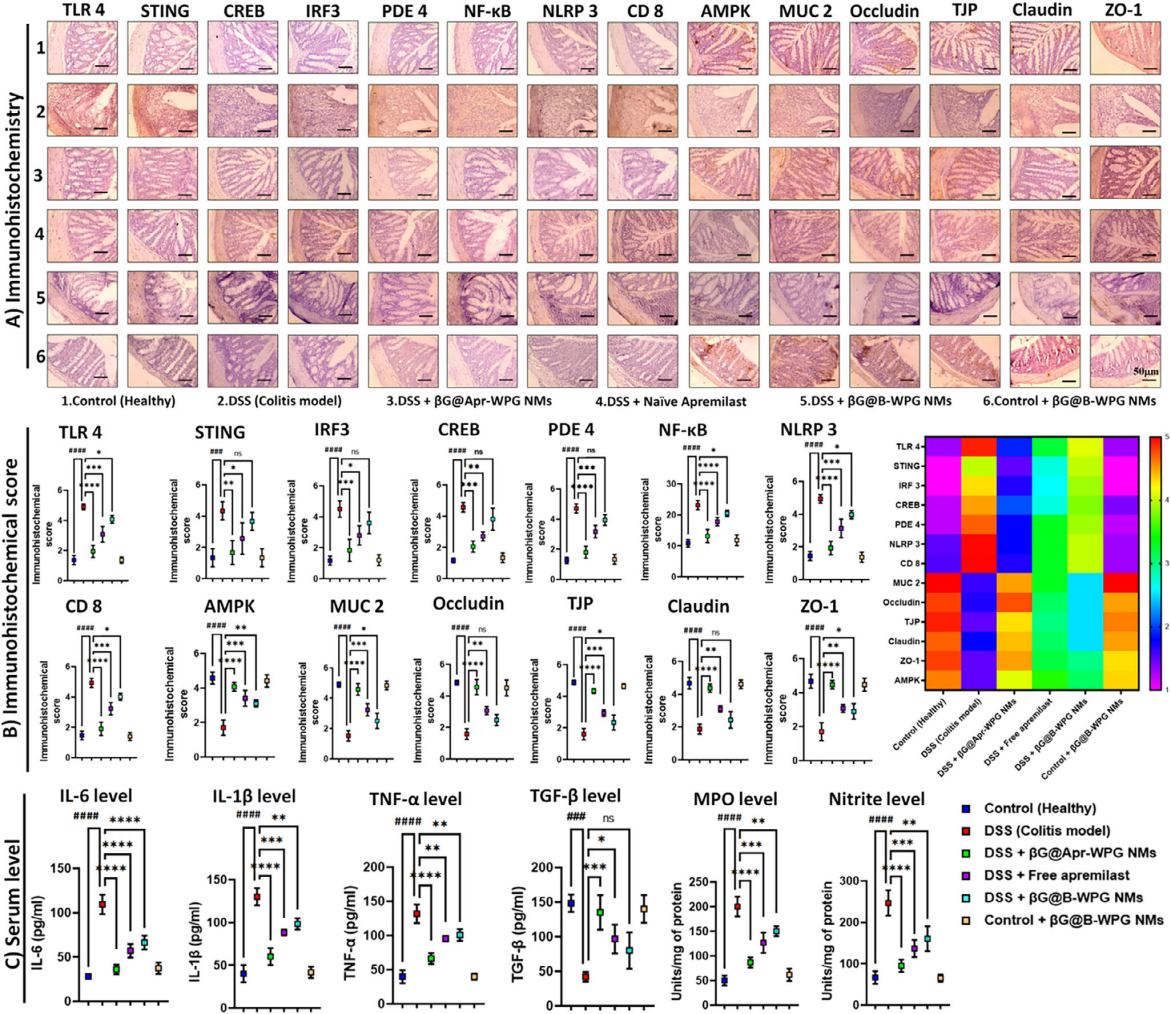
βG@Apr‐WPG NMs mitigate colitis symptoms by regulating pro‐inflammatory cytokines and restoring gut homeostasis. (A) Immunohistochemical staining revealed that βG@Apr‐WPG NMs suppressed pro‐inflammatory markers (TLR4, STING, IRF3, PDE4, NLRP3, CD8, AMPK) and restored epithelial barrier integrity (MUC2, Occludin, TJP, Claudin, ZO‐1) in colitis mice. Colitis conditions elevated pro‐inflammatory factors but reduced gut repair markers, which were reversed by βG@Apr‐WPG NMs treatment. Scale bars: 50 µm (40X magnification). (B) Heatmap analysis quantified immunopositive cells for TLR4, STING, IRF3, PDE4, NLRP3, CD8, AMPK, MUC2, Occludin, TJP, Claudin, and ZO‐1. (C) ELISA results showed reduced levels of inflammatory cytokines (IL‐6, IL‐1β, TNF‐α, TGF‐β) and markers (MPO, nitrite) in serum after βG@Apr‐WPG NMs treatment. (Data are expressed as mean ± SD; statistical significance is denoted as #*p* < 0.05, ##*p* < 0.01, ###*p* < 0.001, ####*p* < 0.0001 vs. Control group; *p < 0.05, **p < 0.01, ***p < 0.001, ****p < 0.0001 vs. DSS group, ns—non‐significant).

We performed immunohistochemical analysis to evaluate key biomarkers associated with UC, including TLR4, STING, IRF3, CREB, PDE4, NF‐κB, NLRP3, CD8, AMPK, MUC2, Occludin, TJP, Claudin, and ZO1 (Figure 5A,B). Treatment with βG@Apr‐WPG NMs significantly reduced pro‐inflammatory markers (TLR4, STING, NF‐κB, NLRP3, CD8) and induced anti‐inflammatory and epithelial integrity biomarkers (AMPK, MUC2, Occludin, TJP, Claudin, ZO1) than free apremilast and DSS + βG@B‐WPG NMs treatment. These results highlight the superior potential of βG@Apr‐WPG NMs in modulating inflammatory pathways and restoring gut barrier function in a DSS‐induced colitis model compared to free apremilast and DSS + βG@B‐WPG NMs. The enhanced efficacy of βG@Apr‐WPG NMs is attributed to their unique composition and mode of action. Apremilast, a PDE4 inhibitor, modulates cAMP levels and suppresses NF‐κB activation. Encapsulation within βG@Apr‐WPG NMs improves its bioavailability and targeted delivery. Additionally, the inclusion of tryptophan and glutathione supports microbial homeostasis and reduces oxidative stress. Tryptophan metabolites, such as indole derivatives, promote beneficial commensals, and strengthen epithelial barrier function, while glutathione mitigates oxidative damage and supports mucosal healing.

Our findings demonstrate that βG@Apr‐WPG NMs address the inflammatory cascade, promote gut microbiota homeostasis, and enhance epithelial repair, offering a multifaceted therapeutic approach for UC. The significant reduction in pro‐inflammatory markers and induction of anti‐inflammatory and barrier integrity biomarkers underscore their potential as a promising therapeutic strategy, with superior efficacy compared to free apremilast and DSS + βG@B‐WPG NMs. These results highlight the importance of integrating targeted drug delivery with microbial and oxidative stress modulation in treating inflammatory bowel diseases.

#### 3.3.4 Therapeutic Efficacy of βG@Apr‐WPG NMs in Modulating Inflammatory Cytokines, Oxidative Stress, and Neutrophil Infiltration in a DSS‐Induced Colitis Model

To investigate the therapeutic efficacy of βG@Apr‐WPG NMs in a DSS‐induced colitis model by analyzing key serum parameters associated with UC. Interleukin‐6 (IL‐6), a pro‐inflammatory cytokine, was significantly elevated in the colitis group, reflecting its role in driving chronic inflammation and tissue damage. Treatment with βG@Apr‐WPG NMs markedly reduced IL‐6 levels, demonstrating their ability to suppress systemic inflammation. Similarly, interleukin‐1 beta (IL‐1β), another critical mediator of inflammation, was significantly lowered by βG@Apr‐WPG NMs, highlighting their potential to modulate inflammatory cascades.

Tumor necrosis factor‐alpha (TNF‐α), a central cytokine in UC pathogenesis, was also elevated in the colitis group, contributing to mucosal barrier disruption and immune dysregulation. βG@Apr‐WPG NMs effectively reduced TNF‐α level, outperforming free apremilast and DSS + βG@B‐WPG NMs, indicating their superior anti‐inflammatory properties. In contrast, TGF‐β, an anti‐inflammatory cytokine that promotes tissue repair and immune tolerance, was significantly enhanced by βG@Apr‐WPG NMs treatment, further supporting their role in restoring immune homeostasis.

MPO activity, a marker of neutrophil infiltration, was significantly elevated in the colitis group, reflecting heightened inflammation. βG@Apr‐WPG NMs treatment markedly reduced MPO activity, indicating decreased neutrophil recruitment and inflammation. Additionally, nitrite levels, a nitric oxide generating indicator linked to oxidative stress and inflammation, were significantly lowered by βG@Apr‐WPG NMs, further underscoring their antioxidant and anti‐inflammatory effects (Figure 5C).

Collectively, the analysis of IL‐1β, IL‐6, TGF‐β, TNF‐α, MPO, and nitrite levels demonstrates the multifaceted therapeutic efficacy of βG@Apr‐WPG NMs in alleviating colitis symptoms than free apremilast and DSS + βG@B‐WPG NMs treatment. By suppressing pro‐inflammatory cytokines, enhancing anti‐inflammatory signaling, reducing neutrophil infiltration, and mitigating oxidative stress, βG@Apr‐WPG NMs offer a promising nanotherapeutic strategy for managing UC, with superior efficacy compared to free apremilast and DSS + βG@B‐WPG NMs.

#### 3.3.5 βG@Apr‐WPG NMs Modulate Pro‐ and Anti‐Inflammatory Cytokines to Restore Immune Homeostasis and Alleviate Colitis in a DSS‐Induced Model

**FIGURE 6:**
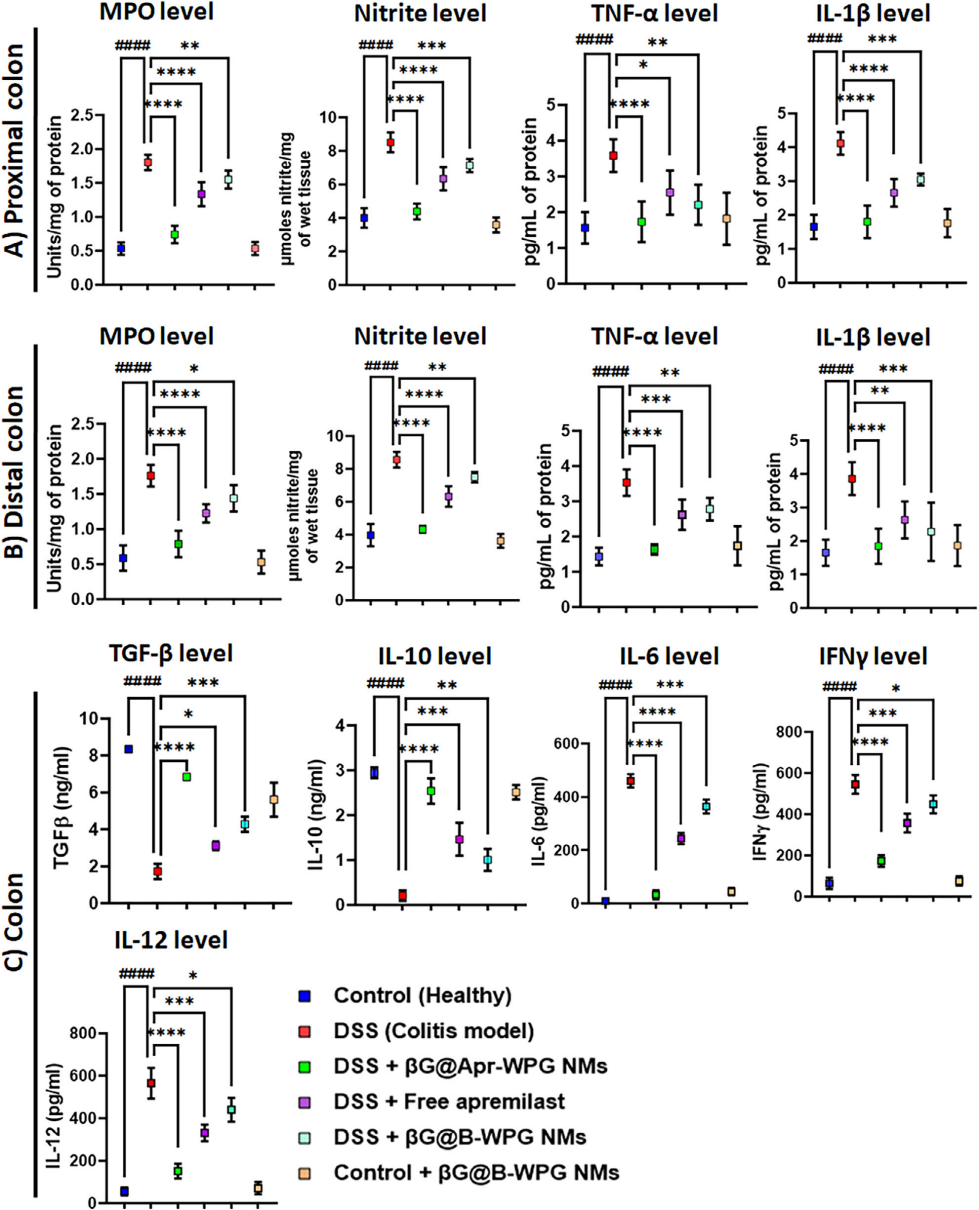
Pro‐ and anti‐inflammatory cytokine levels in the colon of colitis models. (A) Pro‐inflammatory cytokine levels in the proximal colon (MPO, nitrite, TNF‐α, and IL‐1β levels) (*n* = 8). (B) Pro‐inflammatory cytokine levels in the distal colon (MPO, nitrite, TNF‐α, and IL‐1β levels) (*n* = 8). (C) Levels of anti‐inflammatory cytokines (TGF‐β, IL‐10) and pro‐inflammatory cytokines (IL‐6, IFN‐γ, IL‐12) in the whole colon (*n* = 8). (Data are expressed as mean ± SD; statistical significance is denoted as #*p* < 0.05, ##*p* < 0.01, ###*p* < 0.001, ####*p* < 0.0001 vs. Control group; *p < 0.05, **p < 0.01, ***p < 0.001, ****p < 0.0001 vs. DSS group).

To further assess the therapeutic efficacy of βG@Apr‐WPG NMs, we performed a detailed analysis of pro‐inflammatory and anti‐inflammatory biomarkers in both proximal and distal colon tissues, as well as whole colon tissue, in a colitis model (induced with 2.5% DSS). The chemically induced models of colitis exhibited heterogeneous inflammation, with more severe effects observed in the distal colon compared to the proximal colon [51]. Consistent with this, we quantified MPO activity, nitrite levels, TNF‐α, and IL‐1β in both proximal (Figure 6A) and distal colon tissues (Figure 6B), while TGF‐β, IL‐10, IL‐6, interferon‐gamma (IFN‐γ), and IL‐12 were analyzed in whole colon tissue (Figure 6C). Remarkably, βG@Apr‐WPG NMs treatment significantly reduced MPO activity and nitrite levels in both proximal and distal colon tissues, indicating decreased neutrophil infiltration and oxidative stress. The expression of pro‐inflammatory cytokines, including TNF‐α and IL‐1β, was significantly reduced in both proximal and distal colon tissues compared to the colitis control. Notably, the reduction in TNF‐α and IL‐1β levels was more pronounced in the βG@Apr‐WPG NMs group than in the free apremilast and DSS + βG@B‐WPG NMs treatment groups, highlighting the superior anti‐inflammatory efficacy of βG@Apr‐WPG NMs (Figure 6A,B).

In whole colon tissue, βG@Apr‐WPG NMs treatment significantly enhanced the levels of anti‐inflammatory cytokines, such as TGF‐β and IL‐10, which play critical roles in promoting tissue repair, immune tolerance, and mucosal healing. TGF‐β is known to suppress inflammatory responses and facilitate the differentiation of regulatory T cells (Tregs), while IL‐10 inhibits the production of pro‐inflammatory cytokines and supports gut barrier integrity. Conversely, βG@Apr‐WPG NMs significantly reduced the levels of pro‐inflammatory cytokines, including IL‐6, IFN‐γ, and IL‐12. IFN‐γ and IL‐12 are key drivers of Th1‐mediated inflammation, which is central to the pathogenesis of ulcerative colitis. By modulating these cytokines, βG@Apr‐WPG NMs effectively restored immune homeostasis and mitigated inflammation than free apremilast and DSS + βG@B‐WPG NMs treatment (Figure 6C).

Collectively, these results demonstrate that βG@Apr‐WPG NMs not only suppress pro‐inflammatory signaling but also enhance anti‐inflammatory and tissue‐repair mechanisms, offering a comprehensive therapeutic approach for ulcerative colitis. The superior efficacy of βG@Apr‐WPG NMs compared to free apremilast and DSS + βG@B‐WPG NMs treatment underscores their potential as a promising nanotherapeutic strategy for managing inflammatory bowel diseases.

### 3.4 ΒG@Apr‐WPG NMs Treatment Modulates Gut Microbiota and Demonstrates Safety in DSS‐Induced Colitis Mice Model

**FIGURE 7:**
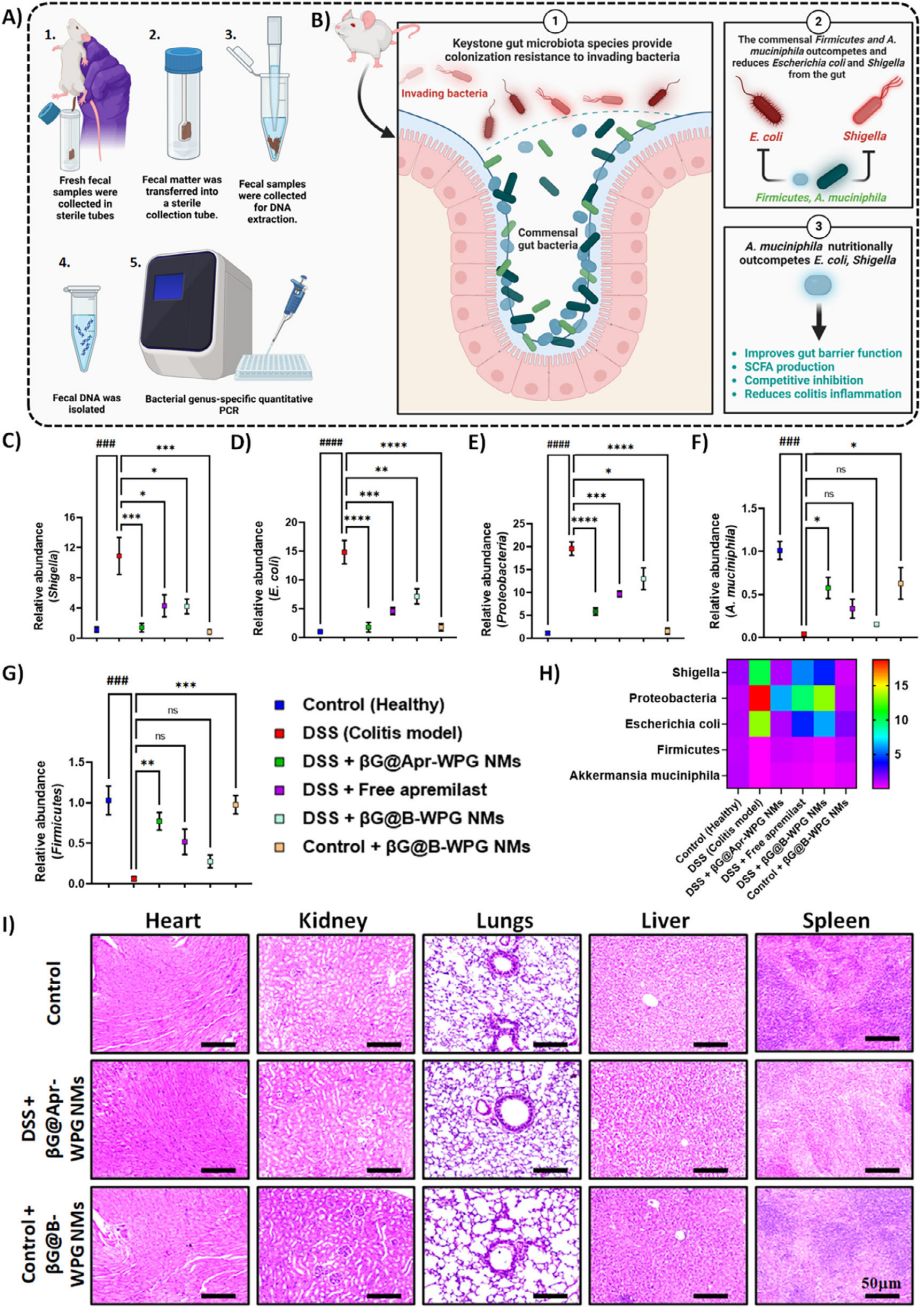
Analysis of gut microbiota–brain interactions regulated by βG@Apr‐WPG NMs. (A) Schematic illustration of aseptic fecal samples collection and bacterial genus‐specific quantitative PCR analysis. Created with Biorender.com. (B) Pictorial representation of the mechanism by which commensal bacteria outcompete pathogenic invaders. (C–G) RT‐qPCR analysis of microbial alterations in fecal samples: pathogenic bacteria (C) *Shigella*, (D) *E. coli*, (E) *Proteobacteria*, and commensal bacteria regulating gut homeostasis (F) *Akkermansia muciniphila* and (G) *Firmicutes*. (Data are expressed as mean ± SD; statistical significance is denoted as #*p* < 0.05, ##*p* < 0.01, ###*p* < 0.001, ####*p* < 0.0001 vs. Control group; *p < 0.05, **p < 0.01, ***p < 0.001, ****p < 0.0001 vs. DSS group, ns—non‐significant). (H) Heatmap quantification of microbial alterations in the same fecal samples (*n* = 8). (I) Safety evaluation of βG@Apr‐WPG NMs: H&E‐stained photomicrographs of heart, kidney, lungs, liver, and spleen from Control, DSS + βG@Apr‐WPG NMs, and Control + βG@B‐WPG NMs treatment groups. Scale bars: 50 µm (40X magnification).

In our study, we evaluated the efficacy of βG@Apr‐WPG NMs in modulating microbial dysbiosis in a DSS‐induced colitis model, characterized by a pathological shift in gut microbiota composition [52]. The colitis model exhibited a significant reduction in beneficial Firmicutes and Akkermansia muciniphila [53], alongside an overgrowth of pro‐inflammatory species such as Shigella [54], E. coli [55], and Enterobacteriaceae. These alterations compromise mucosal integrity, facilitating bacterial translocation and systemic release of pro‐inflammatory cytokines (e.g., TNF‐α, IL‐6), which activate microglia and disrupt BBB function [56]. Further analysis revealed that the restoration of commensal Firmicutes and A. muciniphila played a critical role in outcompeting pathogenic bacteria for nutritional resources and ecological niches. Firmicutes metabolize dietary fibers into short‐chain fatty acids (SCFAs), such as butyrate, which serve as an energy source for colonocytes and exert anti‐inflammatory effects [57]. Similarly, A. muciniphila degrades mucin, producing acetate and propionate, which enhance gut barrier integrity and immune regulation [58]. By promoting these beneficial microbes, βG@Apr‐WPG NMs created a competitive environment that limited the growth of pro‐inflammatory Shigella, E. coli, and Enterobacteriaceae. This nutritional competition, coupled with SCFA production, established an unfavorable milieu for pathogenic overgrowth, reducing their abundance and mitigating inflammation (Figure 7A,B).

Additionally, we explored the role of tryptophan and glutathione in microbial homeostasis. Tryptophan, an essential amino acid, is metabolized by gut microbiota into bioactive compounds, such as indole derivatives and serotonin, which are crucial for maintaining intestinal barrier function and immune regulation [59]. Glutathione, a potent antioxidant, reduces oxidative stress and supports the growth of beneficial microbes, including Firmicutes and A. muciniphila [60]. Treatment with βG@Apr‐WPG NMs enhanced the availability of tryptophan and glutathione, further promoting commensal bacteria and suppressing pro‐inflammatory pathogens. This dual mechanism underscores the therapeutic potential of βG@Apr‐WPG NMs in restoring microbial balance and alleviating colitis‐associated inflammation.

Our findings demonstrated that βG@Apr‐WPG NMs effectively reversed dysbiotic changes in fecal samples, restoring Firmicutes and A. muciniphila levels while significantly reducing Shigella, E. coli, and Enterobacteriaceae abundance (Figure 7C–G). The therapeutic efficacy of βG@Apr‐WPG NMs surpassed that of free apremilast and DSS + βG@B‐WPG NMs, although partial recovery was observed in these groups. The heatmap quantification of microbial alterations in fecal samples is illustrated in Figure 7H. RT‐quantitative polymerase chain reaction (qPCR) data further confirmed the ability of βG@Apr‐WPG NMs to suppress pathogenic overgrowth and restore commensal bacteria, essential for gut homeostasis. By reversing pathological microbial shifts and suppressing pro‐inflammatory cytokines, βG@Apr‐WPG NMs favorably modulated key microbial signatures associated with DSS‐induced colitis, suggesting partial restoration of intestinal microbial homeostasis. These findings position βG@Apr‐WPG NMs as a promising therapeutic strategy for ulcerative colitis, offering a multifaceted approach to managing microbial dysbiosis and its inflammatory consequences.

Assessing the toxicological effects of βG@Apr‐WPG NMs nanomedicines is critical for ensuring safety by evaluating their effects on vital organs, including the liver, kidneys, spleen, heart, and lungs. Histological analysis revealed well‐preserved tissue architecture in all organs across Control and treated groups (DSS + βG@Apr‐WPG NMs and Control + βG@B‐WPG NMs). Liver samples showed healthy hepatocytes with no signs of inflammation or vascular abnormalities. Kidney tissues displayed intact renal tubules and glomerular tufts without cellular infiltration. Spleen segments exhibited normal lymphoid follicles and no inflammatory cell influx. Heart tissues showed no cellular infiltration or cytoplasmic/nuclear damage. Lungs analysis confirmed preserved alveolar structures and bronchiolar epithelia, with no signs of inflammation or tissue destruction (Figure 7I). Serum biomarkers of liver and kidney function were evaluated to assess the systemic safety profile of blank nanomicelles (Control, and Control + βG@B‐WPG NMs group), confirming their safety for administration (Table S2). In order to evaluate the biosafety of βG@Apr‐WPG NMs, hemolysis assay was conducted, revealing no significant hemolysis in the range of tested concentrations and thus a good hemocompatibility of the nanoplatform (Figure S15). These findings demonstrate that βG@Apr‐WPG NMs do not induce adverse effects on vital organs, supporting their safety and therapeutic potential for treating IBD.

### 3.5 Therapeutic Efficacy of βG@Apr‐WPG NMs in Alleviating Neurocognitive Impairment and Neuroinflammation in Colitis Model

**FIGURE 8:**
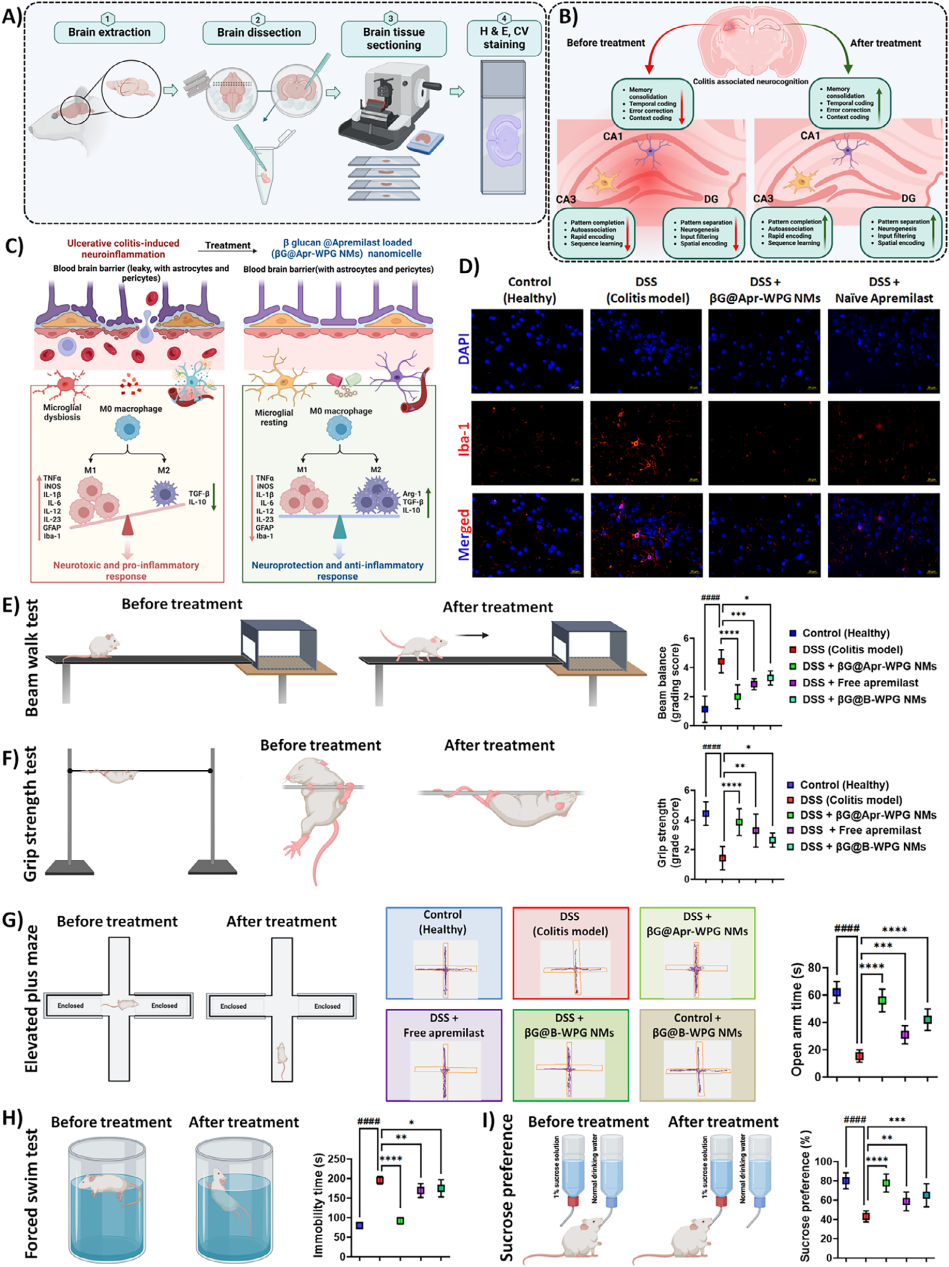
βG@Apr‐WPG NMs enhance learning and cognitive function in IBD mice. (A) Schematic illustration of brain extraction and isolation of the hippocampal region for analysis. (B,C) Pictorial mechanism of action of the dentate gyrus (DG), cornu ammonis 1 (CA1), and cornu ammonis 3 (CA3) in alleviating neurocognitive impairment in ulcerative colitis through βG@Apr‐WPG NMs treatment. (D) Immunofluorescence staining of Iba1 in the hippocampal region of control (healthy), DSS (colitis model), DSS + βG@Apr‐WPG NMs, and DSS + naive apremilast groups. Scale bars: 20 µm. (E) Beam walk test: Schematic and quantified results (*n* = 8). (F) Grip strength test: Schematic and quantified results (*n* = 8). (G) Elevated Plus Maze (EPM) test: Schematic and quantified results (*n* = 8). βG@Apr‐WPG NMs‐treated mice spent significantly more time in the open arms, indicating recovery from anxiety‐like behavior induced by DSS treatment. (H) Forced Swim Test (FST): Schematic and quantified results (*n* = 8). βG@Apr‐WPG NMs‐treated mice showed reduced immobility time, suggesting recovery from depression‐like behavior (I) Sucrose Preference Test (SPT) showed that the experimental groups that received the βG@Apr‐WPG NMs regained their sucrose preference, suggesting recovery from anhedonia‐like behavior and gut–brain axis‐associated neurobehavioral dysfunction in mice with DSS‐induced colitis (*n* = 8). (Data are expressed as mean ± SD; statistical significance is denoted as #*p* < 0.05, ##*p* < 0.01, ###*p* < 0.001, ####*p* < 0.0001 vs. Control group; *p < 0.05, **p < 0.01, ***p < 0.001, ****p < 0.0001 vs. DSS group). (A), (B), (C), (E), (F), (G), (H), and (I) schematics created with Biorender.com.

UC is a chronic inflammatory bowel disease (IBD) characterized by persistent inflammation of the colon, leading to symptoms, such as abdominal pain, diarrhea, and mucosal ulceration. Beyond gastrointestinal manifestations, UC is increasingly recognized for its systemic effects, including neurocognitive impairments such as anxiety, stress, and depression. These neuropsychiatric symptoms are thought to arise from the gut–brain axis, a bidirectional communication system linking the gut microbiota, immune responses, and the central nervous system (CNS) [61, 62]. Chronic inflammation in UC disrupts this axis, leading to the release of pro‐inflammatory cytokines (e.g., TNF‐α, IL‐6, IL‐1β) that can cross the BBB and activate microglia, the resident immune cells of the brain [63]. This microglial activation, marked by increased expression of ionized calcium‐binding adapter molecule 1 (Iba‐1), contributes to neuroinflammation and subsequent neurocognitive dysfunction [16]. The hippocampus, vital for memory, learning, and emotional regulation, is highly susceptible to inflammation and oxidative stress. In UC, systemic inflammation disrupts the gut–brain axis, impairing hippocampal function and leading to neurocognitive deficits [9]. Studying hippocampal changes in UC provides insights into neuroinflammation and guides therapies targeting both gastrointestinal and neuropsychiatric symptoms (Figure 8A–C).

To further elucidate the mechanisms underlying these improvements, we performed immunohistochemical analysis of hippocampal tissue sections, focusing on the dentate gyrus (DG) and cortex regions. These brain areas are critical for memory, learning, and emotional regulation, and are particularly vulnerable to neuroinflammation. We assessed the expression of key biomarkers associated with neuroinflammation and BBB integrity, including TNF‐α, IL‐1β, inducible nitric oxide synthase (iNOS), nuclear factor‐kappa B (NF‐κB), cyclooxygenase‐2 (COX‐2), IL‐18, fibronectin, beclin, occludin, and claudin. TNF‐α and IL‐1β are pro‐inflammatory cytokines that play a central role in neuroinflammation, promoting microglial activation and neuronal damage. iNOS and COX‐2 are enzymes involved in the production of nitric oxide and prostaglandins, respectively, which exacerbate oxidative stress and inflammation. NF‐κB is a transcription factor that regulates the expression of these pro‐inflammatory mediators, while IL‐18 is a cytokine that amplifies inflammatory responses. Fibronectin, a glycoprotein involved in extracellular matrix remodeling, is often upregulated in neuroinflammatory conditions, contributing to tissue damage. Conversely, beclin, a key autophagy‐related protein, supports cellular homeostasis by clearing damaged organelles and proteins. Occludin and claudin are tight junction proteins critical for maintaining BBB integrity, which is often compromised in neuroinflammatory conditions. Our immunohistochemical analysis revealed that DSS‐induced colitis mice exhibited elevated expression of TNF‐α, IL‐1β, iNOS, NF‐κB, COX‐2, IL‐18, and fibronectin in the hippocampal DG and cortex regions, along with reduced levels of beclin, occludin, and claudin. These findings indicate heightened neuroinflammation, oxidative stress, and BBB disruption in the colitis model. However, treatment with βG@Apr‐WPG NMs significantly reduced the expression of pro‐inflammatory markers (TNF‐α, IL‐1β, iNOS, NF‐κB, COX‐2, IL‐18, and Fibronectin (FN)) and restored the levels of beclin, occludin, and claudin, indicating a reduction in neuroinflammation and restoration of BBB integrity than free apremilast and DSS + βG@Apr‐WPG NMs treatment (Figure S16). The restoration of BBB integrity by βG@Apr‐WPG NMs is particularly significant, as it prevents the infiltration of peripheral inflammatory mediators into the CNS, thereby mitigating neuroinflammation and its downstream effects on neurocognition. The upregulation of beclin suggests enhanced autophagy, which may further contribute to the clearance of inflammatory debris and cellular repair.

Furthermore, immunofluorescence analysis [64] of brain tissues demonstrated a significant reduction in IbaS‐1 expression in the βG@Apr‐WPG NMs‐treated group compared to the DSS‐induced colitis group. This reduction in ionized calcium‐binding adapter molecule‐1 (IBA‐1), a marker of microglial activation, suggests that βG@Apr‐WPG NMs effectively mitigate neuroinflammation (Figure 8D). The mechanism underlying this effect likely involves the modulation of systemic inflammation and gut microbiota homeostasis by βG@Apr‐WPG NMs. By restoring gut microbial balance and reducing the release of pro‐inflammatory cytokines, βG@Apr‐WPG NMs prevent the activation of microglia and subsequent neuroinflammation, thereby alleviating neurocognitive symptoms such as anxiety, stress, and depression.

In this study, we investigated the impact of βG@Apr‐WPG NMs on neurocognitive impairment in a DSS‐induced colitis model. Behavioral tests, including the beam walk test and grip strength test, were employed to assess motor coordination, anxiety‐like behavior, and muscle strength, which are often compromised in neuroinflammatory conditions. The beam walk test evaluates balance and coordination, while the grip strength test measures neuromuscular function, both of which are sensitive to CNS inflammation and stress. Our results revealed that DSS‐induced colitis mice exhibited significant deficits in these tests, reflecting neurocognitive and motor impairments. However, treatment with βG@Apr‐WPG NMs restored performance in both the beam walk (Figure 8E and Video S2) and grip strength tests (Figure 8F and Figure S17, Video S3) to levels comparable to the healthy control group, indicating a reversal of neurocognitive dysfunction.

In order to further investigate gut–brain axis‐associated behavior changes, anxiety‐ and depression‐like behaviors were evaluated using the Elevated Plus Maze (EPM), Forced Swim Test (FST) and Sucrose Preference Test (SPT). Colitis mice showed significant behavioral deficits, such as reduced open arm exploration in the EPM, increased immobility time in the FST and, decreased sucrose preference which was observed in DSS‐induced colitis mice. Important, the treatment with βG@Apr‐WPG NMs significantly reduced these behavioral abnormalities, and the number of open arms exploration, the duration of immobility, and the sucrose consumption were recovered relative to the DSS group. The results suggest that βG@Apr‐WPG NMs significantly reverse anxiety‐like, depressive‐like and anhedonic behaviors resulting from colitis‐induced gut–brain axis dysfunction (Figure 8G–I and Figure S18). In conclusion, our study demonstrates that βG@Apr‐WPG NMs effectively modulate gut microbiota, reduce systemic and neuroinflammation, and restore neurocognitive function in a DSS‐induced colitis model. By targeting key biomarkers of neuroinflammation and BBB integrity, βG@Apr‐WPG NMs offer a promising therapeutic strategy for addressing the neurocognitive complications of UC, highlighting their potential to improve both gastrointestinal and neuropsychiatric outcomes in patients with IBD.

#### 3.5.1 H&E and CV Staining Reveal βG@Apr‐WPG NMs Mitigate Neuroinflammation and Restore Neuronal Integrity in UC

To further investigate the impact of UC on neuroinflammation, we focused on histological analysis of hippocampal brain tissue regions, including cornu ammonis 1 (CA1), cornu ammonis 3 (CA3), dentate gyrus (DG), and the cortex, using hematoxylin and eosin (H&E) and cresyl violet (CV) staining. These staining techniques are essential for visualizing cellular and structural changes in brain tissue, providing critical insights into neuroinflammation and neuronal integrity. H&E staining allows for the identification of general tissue morphology, including the presence of pyknotic neurons (shrunken, condensed nuclei indicative of cell death) and inflammatory infiltrates, while CV staining specifically highlights Nissl bodies in neuronal cell bodies, enabling the assessment of neuronal health and density.

**FIGURE 9:**
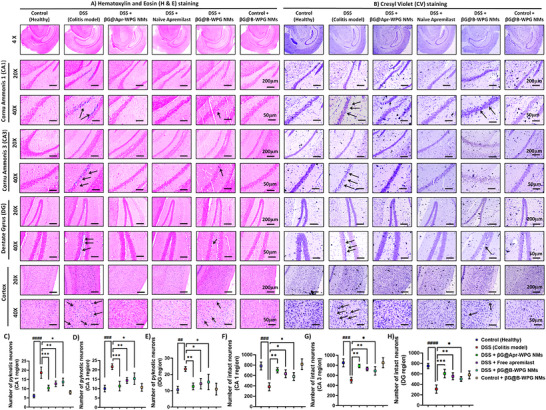
βG@Apr‐WPG NMs mitigate histopathological neurocognitive markers in the cornu ammonis 1 (CA 1), cornu ammonis 3 (CA 3), and dentate gyrus (DG) regions of IBD mice. (A) H&E and (B) Cresyl Violet staining of hippocampal brain tissue, showing neuroinflammation, pyknosis (condensed, shrunken nuclei; black arrows), neuronal loss, and reduced Nissl substance (black arrows), respectively. Increased microglial activation and astrocytosis indicate neuroinflammatory responses. Quantitative analysis of pyknotic neurons in (C) CA1, (D) CA3, and (E) DG regions, and intact neurons in (F) CA1, (G) CA3, and (H) DG regions. Scale bars: 200 µm (20X magnification) and 50 µm (40X magnification). (Data are expressed as mean ± SD; statistical significance is denoted as #*p* < 0.05, ##*p* < 0.01, ###*p* < 0.001, ####*p* < 0.0001 vs. Control group; *p < 0.05, **p < 0.01, ***p < 0.001, ****p < 0.0001 vs. DSS group).

In the DSS‐induced colitis model, we observed significant neuroinflammation, characterized by increased pyknotic neurons and a reduction in intact neurons in the CA1, CA3, DG, and cortex regions. These changes reflect the detrimental effects of systemic inflammation and oxidative stress on hippocampal and cortical integrity, which are closely linked to the neurocognitive impairments observed in UC. The use of H&E and CV staining provided a comprehensive evaluation of neuronal damage and recovery, offering a clear visualization of the pathological changes induced by UC and the therapeutic effects of βG@Apr‐WPG NMs. Treatment with βG@Apr‐WPG NMs significantly reduced the number of pyknotic neurons and enhanced the population of intact neurons in the CA1, CA3, DG, and cortex regions (Figure 9A,B), as quantified in (Figure 9C–H) than free apremilast and DSS + βG@B‐WPG NMs treatment. This restoration of neuronal integrity highlights the ability of βG@Apr‐WPG NMs to mitigate neuroinflammation and protect against neuronal damage in UC. The reduction in pyknotic neurons and preservation of intact neurons in these critical brain regions further support the therapeutic potential of βG@Apr‐WPG NMs in addressing both gastrointestinal and neurocognitive symptoms of UC, underscoring their role in modulating the gut–brain axis and improving overall disease outcomes.

The combination of H&E and CV staining in our study provided a robust methodological approach to assess neuroinflammation and neuronal health, enabling us to demonstrate the efficacy of βG@Apr‐WPG NMs in preserving brain tissue integrity and mitigating UC‐associated neurocognitive deficits. These findings highlight the importance of histological techniques in understanding the complex interplay between systemic inflammation and brain health in UC.

### 3.6 Immune Homeostasis Restoration in Galt by βG@Apr‐WPG NMs

This study investigated the immunomodulatory effects of βG@Apr‐WPG NMs on immune cell dynamics within the gut‐associated lymphoid tissue (GALT) in a murine model of DSS‐induced colitis. Flow cytometry was used to analyze immune subsets isolated from lamina propria (LP) compartments of colonic tissue.

Macrophages and dendritic cells (DCs), which play central roles in gut immunity, are often dysregulated in IBD, contributing to impaired microbial clearance and sustained inflammation. DSS exposure significantly increased the proportions of both cell types in LP compartments.

**FIGURE 10:**
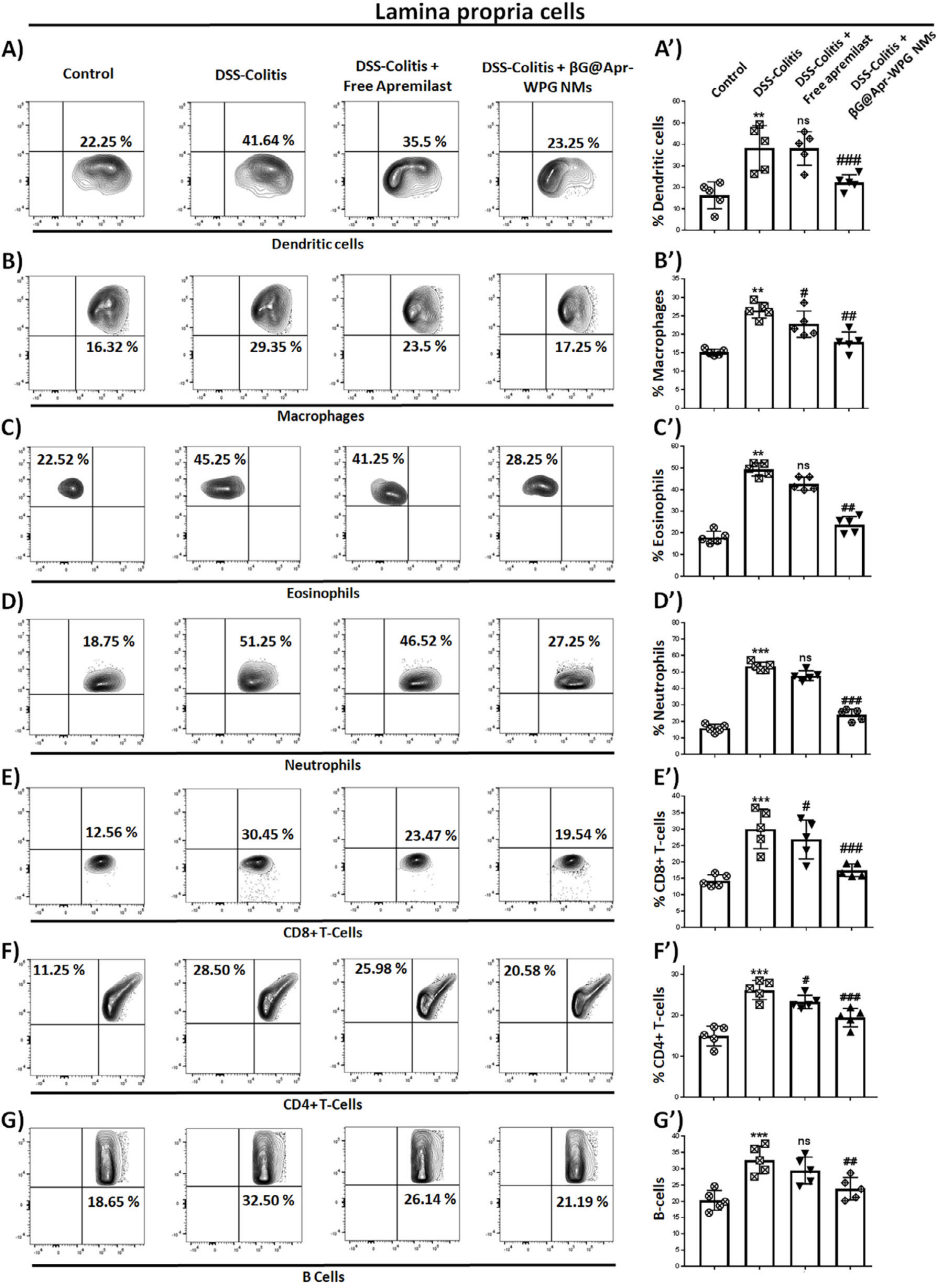
Immunomodulatory impact of βG@Apr‐WPG NMs on myeloid and lymphoid cell populations isolated from the colonic lamina propria cells Quantitative and qualitative flow cytometry analyses were performed to assess immune cell subsets across various treatment groups. Lamina propria‐derived immune cells were evaluated for: (A) Dendritic cells (MHC class II+ SiglecF−) (B) Macrophages marked by dual expression of MHC class II and SiglecF. (C) Eosinophils cells. (D) Neutrophils identified as Ly6G+ CD11c+ double‐positive cells. (E) CD8+ cytotoxic T cells. (F) CD4+ helper T cells (G) B cells. Quantification of all FACS plots was shown in (A’‐G’). Flow cytometry data were quantified for four experimental groups: the Control group, the colitis model group, DSS + Free apremilast and the colitis model treated with βG@Apr‐WPG NMs.

βG@Apr‐WPG NMs administration significantly reduced these populations, unlike free apremilast, which did not elicit comparable changes (Figure 10A,B). Eosinophils, while absent in the esophageal mucosa under normal conditions, are prominent in IBD pathology and correlate with disease severity. Consistent with this, our study detected an increased eosinophil presence in colitis mice, particularly in the LP compartments. βG@Apr‐WPG NMs treatment significantly lowered eosinophil levels in the LP tissue fractions, suggesting attenuation of eosinophil‐driven inflammation (Figure 10C). In the context of inflammation, neutrophils, monocytes, Th17, Th9, and CD8+ T cells are known contributors to proinflammatory cytokine production, including IL‐17 and IL‐6. Our findings revealed significantly elevated neutrophil populations in LP compartments of colitis mice, corroborating previous reports. However, treatment with βG@Apr‐WPG NMs led to a marked reduction in neutrophil frequencies in the LP tissues (Figure 10D). Further analysis of lymphocyte subsets revealed elevated levels of CD4+ helper T cells, CD8+ cytotoxic T cells, and B cells in colitis mice, consistent with chronic intestinal inflammation. Notably, βG@Apr‐WPG NMs treatment effectively decreased these proinflammatory lymphocyte populations in the LP fractions (Figure 10E–G) unlike the free apremilast group as further supported by quantification in (Figure 10A′–G′). The gating strategy and representative dot plots are shown in Figure S19.

These results collectively suggest that βG@Apr‐WPG NMs mitigate inflammation by modulating key immune cell subsets implicated in IBD pathogenesis. Their ability to restore immune balance and suppress overactive myeloid and lymphoid responses underlines their therapeutic potential for ulcerative colitis.

### 3.7 ΒG@Apr‐WPG NMs Attenuate Microglial Activation Induced by Experimental Colitis

**FIGURE 11:**
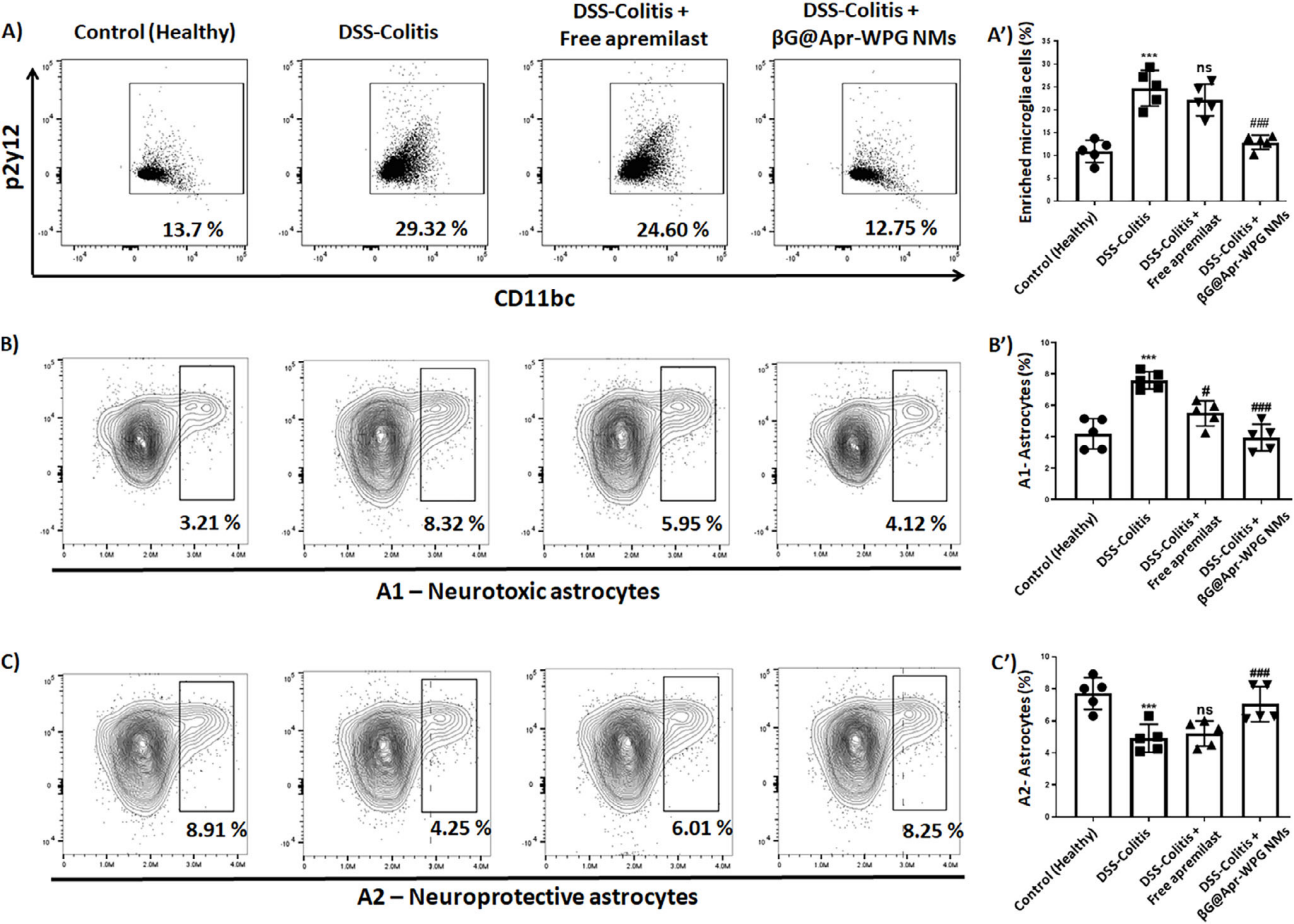
βG@Apr‐WPG NMs attenuate microglial activation induced by experimental colitis. Flow cytometric analysis was performed on brain‐derived single‐cell suspensions after colitis induction. Microglia were identified as CD45^+^CD11b/c^+^P2Y12^+^ cells following exclusion of doublets and dead cells. Gating strategy included identification of the main population by FSC and SSC. (A) Colitis led to increased microglial size, granularity, and cell number, indicative of activation. (B,C) Treatment with βG@Apr‐WPG nanomicelles significantly reduced these activation markers, restoring microglial parameters toward baseline. Astrocytes were first gated based on FSC/SSC to exclude debris and doublets, followed by viability dye exclusion and selection of GFAP^+^ cells. A1 neurotoxic astrocytes were identified as CD86^+^GFAP^+^, while A2 neuroprotective astrocytes were defined as S100A10^+^GFAP^+^. DSS‐induced mice treated with βG@Apr‐WPG NMs showed a reduced proportion of A1 astrocytes and a corresponding increase in A2 astrocytes compared to untreated controls, indicating effective modulation of neuroinflammation via gut–brain axis regulation. Quantitative analysis of FACS plots, (A’–C’). Flow cytometry data were quantified for four experimental groups: the Control group, the colitis model group, DSS + Free apremilast and the colitis model treated with βG@Apr‐WPG.

Colitis‐induced marked microglial activation in the brain, as demonstrated by increased cell size, granularity, and a significant rise in CD45^+^CD11b/c^+^P2Y12^+^ microglia. These cells exhibited elevated expression of pro‐inflammatory (M1‐like) markers, including CD86 and MHC‐II [65]. To characterize this response, DSS (colitis model) and DSS + βG@Apr‐WPG NMs groups microglia were analyzed using flow cytometry of enriched single‐cell suspensions. Microglia were identified after exclusion of doublets and dead cells and assessed by forward and side scatter profiles. Colitis led increase in microglial count per mg of brain tissue compared to DSS + βG@Apr‐WPG NMs. Treatment with βG@Apr‐WPG nanomicelles significantly attenuated microglial activation, reducing cell size, granularity, and abundance to near‐baseline levels, indicating effective suppression of neuroinflammation driven by peripheral immune signals (Figure 11A). To assess the impact of βG@Apr‐WPG nanomicelles on neuroinflammation, we examined astrocyte polarization into neurotoxic (A1) and neuroprotective (A2) phenotypes. Flow cytometric analysis was conducted on brain tissue from DSS‐induced colitis mice. Cells were gated based on forward and side scatter properties, followed by selection of viable, singlet populations. Astrocytes were identified using GFAP expression, and further classified into A1 and A2 subtypes using CD86 and S100A10 markers, respectively.

Compared to untreated DSS controls, mice treated with βG@Apr‐WPG NMs exhibited a marked reduction in the proportion of A1 (CD86^+^GFAP^+^) astrocytes and a significant increase in A2 (S100A10^+^GFAP^+^) astrocytes, indicating a shift toward a neuroprotective phenotype. This polarization pattern supports the anti‐neuroinflammatory effect of the nanomicelles and their capacity to modulate glial activation through gut‐brain axis regulation (Figure 11B,C). The gating strategy and representative dot plots are shown in Figures S20 and S21 and quantification shown in the (Figure 11A′–C′).

## 4 Conclusion

This study successfully demonstrated the synthesis, characterization, and therapeutic efficacy of βG@Apr‐WPG NMs (β‐glucan armored apremilast encapsulating tryptophan‐PLGA‐glutathione nanomicelles) as a targeted method of administering medication for UC. By leveraging a nanomicellar platform incorporating apremilast, beta‐glucan, and tryptophan‐PLGA‐glutathione (WPG) conjugates, the system exhibited remarkable gastroprotection, inflammation‐responsive drug release, and enhanced therapeutic potential. The nanomicelles remained stable in simulated gastric environments while facilitating controlled apremilast release at inflamed colonic sites. Their anti‐inflammatory properties were validated through in vitro and in vivo studies, highlighting their ability to suppress pro‐inflammatory cytokines (TNF‐α, IL‐6, IL‐1β), modulate oxidative stress markers, and enhance anti‐inflammatory responses (IL‐10, TGF‐β).

The therapeutic efficacy of βG@Apr‐WPG NMs in a DSS‐induced colitis model confirmed their potential in alleviating intestinal inflammation, preserving gut barrier integrity, and restoring microbial homeostasis. Histopathological analyses revealed significant protection against mucosal damage, crypt loss, and immune cell infiltration. Notably, βG@Apr‐WPG NMs effectively reduced mast cell infiltration, modulated tight junction proteins, and enhanced mucin layer stability, ensuring improved gut epithelial defense mechanisms. Furthermore, their ability to regulate gut microbiota composition by promoting beneficial Firmicutes and Akkermansia muciniphila while reducing pathogenic Shigella and E. coli suggests a multifaceted approach to UC treatment that integrates both immune modulation and microbial balance.

Beyond gastrointestinal benefits, this study highlighted the gut–brain axis's role in UC‐related neuroinflammation. Treatment with βG@Apr‐WPG NMs significantly reduced microglial activation, oxidative stress, and neuroinflammatory markers (TNF‐α, IL‐1β, NF‐κB, iNOS, COX‐2) while restoring BBB integrity and neuronal health. Behavioral tests demonstrated improved cognitive function and motor coordination, reinforcing the potential of βG@Apr‐WPG NMs in mitigating UC‐associated neurocognitive impairments.

**SCHEME 2:**
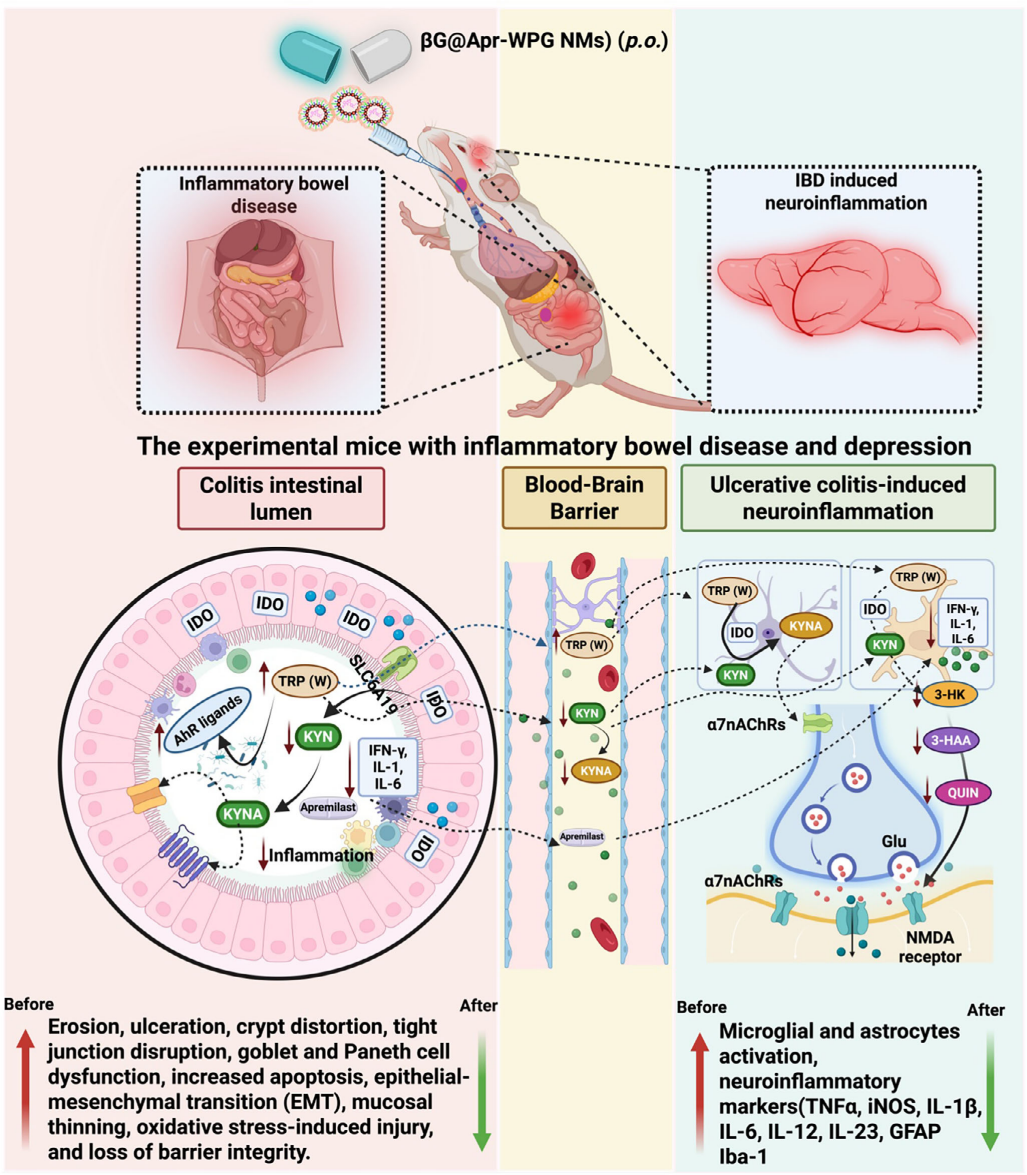
βG@Apr‐WPG NMs alleviates colon inflammation and neuroinflammation in colitis mice: The gut–brain axis in IBD. The link between intestinal inflammation, the kynurenine pathway (KP), and neurocognitive disorders (anxiety, depression, neuroinflammation) in IBD. Inflammatory activity in IBD triggers the production of cytokines (IFN‐γ, IL‐6, IL‐1), activating IDO and increasing tryptophan (TRP) degradation to kynurenine (KYN). KYN crosses the blood–brain barrier and is metabolized into neurotoxic metabolites (QUIN, 3‐HK, 3‐HAA) and neuroprotective KYNA. Neurotoxic metabolites contribute to hippocampal neuron damage and depression, while KYNA exerts neuroprotective effects. These KP metabolites influence mood through complex neurobiological responses. (Abbreviations: IFN, interferon; IL, interleukin; α7nAChRs, α7 nicotinic acetylcholine receptors; Glu, glutamate; NMDA, *N*‐methyl‐D‐aspartate). βG@Apr‐WPG NMs regulate tryptophan levels, mitigating colitis symptoms, oxidative stress, and inflammation while providing neuroprotective effects in gut–brain inflammatory conditions. Created with Biorender.com.

Importantly, βG@Apr‐WPG NMs exhibited favorable preliminary biocompatibility and safety profile, with no observed toxicity in vital organs, validating their translational potential. Compared to free apremilast, βG@Apr‐WPG NMs showcased superior drug stability, bioavailability, and therapeutic performance, underscoring their viability as an advanced nanotherapeutic for UC. These findings collectively suggest that βG@Apr‐WPG NMs provide a comprehensive therapeutic strategy that addresses both intestinal inflammation and its systemic consequences, offering a promising platform for the effective management of UC and other IBDs (Scheme 2).

Although the present results show that βG@Apr‐WPG NMs have therapeutic promise for intestinal inflammation, barrier restoration, gut dysbiosis and behavioral dysfunction associated with neuroinflammation, certain limitations have to be taken into account. The DSS induced colitis model used in the present study was useful in recapitulating key features of ulcerative colitis, such as sustained intestinal inflammation, loss of barrier function, microbial imbalance, and gut–brain axis‐associated behavioral impairments, but lacks the multi‐stage nature and longer duration of neuropsychiatric effects in chronic IBD patients. Hence, future studies using long‐term models of colitis, human‐relevant models, and longer neurobehavioral assessments will be beneficial for further validating the translatability of therapeutic findings in this study. The exact mechanisms of gut–brain communication mediated by microbial metabolites, vagal signaling pathways, and systemic immune mediators remain to be well understood. Moreover, in‐depth sequencing of the microbiome, pharmacokinetic analysis, long‐term immunotoxicity analysis, and precision investigations like fecal microbiota transplantation will be helpful for further validation of the therapeutic mechanism of the developed nanoplatform. Future investigation into these aspects will help to understand regulation of the microbiota–gut–brain axis and help bring βG@Apr‐WPG NMs to the clinic for ulcerative colitis and related neurological disorders. The therapeutic efficacy of βG@Apr‐WPG NMs was validated in a well‐established murine colitis model but further studies using human intestinal organoids, human primary colonocytes, patient‐derived samples, and humanized mouse models will help confirm the translation of the therapeutic effects and better elucidate human‐specific mechanisms of microbiota–gut–brain axis regulation.

## Author Contributions

**Chandrashekhar Jori**: conceptulization, methodology, software, data curation, formal analysis, validation, investigation, writing – original draft, visualization. **Ahmed Shaney Rehman**: investigation, methodolgy, data curation, formal analysis. Taruna Lamba: methodology, software. **Anas Ahmad**: software, formal analysis. Jattin Kumar: investigation, methodology. **Aneesh Ali**: methodology, software. **Ajesh Joshi**: methodolgy, visualization, investigation. **Ashraf Ali**: methodology, investigation, visualization. **Suhel Parvez**: conceptualization, investigation, funding acquisition, validation, formal analysis, software, writing – review and editing, data curation. **Javed N. Agrewala**: conceptualization, supervision, funding acquisition, resources, investigation, software, data curation. **Rehan Khan**: conceptualization, resources, formal analysis, funding acquisition, writing – original draft, wrting – review and editing, visualization, supervision, project administration, software,

## Funding

Department of Science and Technology (DST), ANRF, India with Grant No. ANRF/ARG/2025/005584/LS , Indian Council of Medical Research (ICMR), India with Grant No. FIW‐2025‐01‐00000682 supports this work.

## Conflicts of Interest

The authors declares no conflicts of interests

## Supporting information




**Supporting Information**: advs76566‐sup‐0001‐SuppMat.docx.

**Supplemental Movie 1**: advs76566‐sup‐0002‐Beam walk test.MOV.

**Supplemental Movie 2**: advs76566‐sup‐0003‐Grip strength test.MOV.

**Supplemental Movie 3**: advs76566‐sup‐0004‐Physical activity test.MOV.

## Data Availability

Due to confidentiality of the data, it is available only upon valid request from the corresponding author.

## References

[1] J. F. Cryan , K. J. O'Riordan , C. S. M. Cowan , et al., “The Microbiota‐Gut‐Brain Axis,” Physiological Reviews 99, no. 4 (2019): 1877–2013, 10.1152/physrev.00018.2018. 31460832

[2] M. J. Mitchell , M. M. Billingsley , R. M. Haley , M. E. Wechsler , N. A. Peppas , and R. Langer , “Engineering Precision Nanoparticles for Drug Delivery,” Nature Reviews Drug Discovery 20, no. 2 (2021): 101–124, 10.1038/s41573-020-0090-8. 33277608 PMC7717100

[3] S. Mitragotri , D. G. Anderson , X. Chen , et al., “Accelerating the Translation of Nanomaterials in Biomedicine,” ACS Nano 9, no. 7 (2015): 6644–6654, 10.1021/acsnano.5b03569. 26115196 PMC5227554

[4] C. Jori , A. A. Chaudhary , S. Rashid , et al., “Biomaterial‐Based Strategies for Immunomodulation in IBD: Current and Future Scenarios,” Journal of Materials Chemistry B 11, no. 25 (2023): 5668–5692, 10.1039/D3TB00276D. 37249518

[5] D. Zheng , T. Liwinski , and E. Elinav , “Interaction between Microbiota and Immunity in Health and Disease,” Cell Research 30, no. 6 (2020): 492–506, 10.1038/s41422-020-0332-7. 32433595 PMC7264227

[6] J. S. Loh , W. Q. Mak , L. K. S. Tan , et al., “Microbiota–Gut–Brain Axis and Its Therapeutic Applications in Neurodegenerative Diseases,” Signal Transduction and Targeted Therapy 9, no. 1 (2024): 1–53, 10.1038/s41392-024-01743-1. 38360862 PMC10869798

[7] B. Barberio , M. Zamani , C. J. Black , E. V. Savarino , and A. C. Ford , “Prevalence of Symptoms of Anxiety and Depression in Patients with Inflammatory Bowel Disease: A Systematic Review and Meta‐Analysis,” The Lancet Gastroenterology & Hepatology 6, no. 5 (2021): 359–370, 10.1016/S2468-1253(21)00014-5. 33721557

[8] R. A. Marrie , L. A. Graff , J. D. Fisk , S. B. Patten , and C. N. Bernstein , “The Relationship between Symptoms of Depression and Anxiety and Disease Activity in IBD over Time,” Inflammatory Bowel Diseases 27, no. 8 (2021): 1285–1293, 10.1093/ibd/izaa349. 33393632 PMC8314114

[9] H. He , Q. Qin , F. Xu , et al., “Oral Polyphenol‐Armored Nanomedicine for Targeted Modulation of Gut Microbiota–Brain Interactions in Colitis,” Science Advances 9, no. 21 (2023): adf3887, 10.1126/sciadv.adf3887. PMC10219598 37235662

[10] D. Wu , Q. Chen , X. Chen , F. Han , Z. Chen , and Y. Wang , “The Blood–brain Barrier: Structure, Regulation and Drug Delivery,” Signal Transduction and Targeted Therapy 8, no. 1 (2023): 1–27, 10.1038/s41392-023-01481-w. 37231000 PMC10212980

[11] A. P. Shoubridge , J. M. Choo , A. M. Martin , et al., “The Gut Microbiome and Mental Health: Advances in Research and Emerging Priorities,” Molecular Psychiatry 27, no. 4 (2022): 1908–1919, 10.1038/s41380-022-01479-w. 35236957

[12] L. Liu , J. R. Huh , and K. Shah , “Microbiota and the Gut‐Brain‐Axis: Implications for New Therapeutic Design in the CNS,” eBioMedicine 77, (2022): 103908, 10.1016/j.ebiom.2022.103908. 35255456 PMC8897630

[13] C. Jori , A. Ahmad , A. Kumar , N. Ali , Y.‐O. Son , and R. Khan , “Mucin Armoured Diethylaminoethyl‐Dextran Cloaked Rebamipide Based Oral Nanoformulation for the Therapy of Inflammatory Bowel Disease,” Materials Today Bio 39 (2026): 103322, 10.1016/j.mtbio.2026.103322. PMC13276610 42326067

[14] W. Zhang , D. Xiao , Q. Mao , and H. Xia , “Role of Neuroinflammation in Neurodegeneration Development,” Signal Transduction and Targeted Therapy 8, no. 1 (2023): 1–32, 10.1038/s41392-023-01486-5. 37433768 PMC10336149

[15] K. J. O'Riordan , G. M. Moloney , L. Keane , G. Clarke , and J. F. Cryan , “The Gut Microbiota‐Immune‐Brain Axis: Therapeutic Implications,” Cell Reports Medicine 6, no. 3, (2025): 101982, 10.1016/j.xcrm.2025.101982. 40054458 PMC11970326

[16] I.‐A. Gampierakis , Y. Koutmani , M. Semitekolou , et al., “Hippocampal Neural Stem Cells and Microglia Response to Experimental Inflammatory Bowel Disease (IBD),” Molecular Psychiatry 26, no. 4 (2021): 1248–1263, 10.1038/s41380-020-0651-6. 31969694

[17] J. Suez , N. Zmora , E. Segal , and E. Elinav , “The Pros, Cons, and Many Unknowns of Probiotics,” Nature Medicine 25, no. 5 (2019): 716–729, 10.1038/s41591-019-0439-x. 31061539

[18] N. Zmora , G. Zilberman‐Schapira , J. Suez , et al., “Personalized Gut Mucosal Colonization Resistance to Empiric Probiotics Is Associated with Unique Host and Microbiome Features,” Cell 174, no. 6 (2018): 1388–1405.e21, 10.1016/j.cell.2018.08.041. 30193112

[19] M. Vétizou , J. M. Pitt , R. Daillère , et al., “Anticancer Immunotherapy by CTLA‐4 Blockade Relies on the Gut Microbiota,” Science 350, no. 6264 (2015): 1079–1084, 10.1126/science.aad1329. 26541610 PMC4721659

[20] B. Chassaing , J. De Bodt , M. Marzorati , T. Van de Wiele , and A. T. Gewirtz , “Dietary Emulsifiers Directly Alter Human Microbiota Composition and Gene Expression Ex Vivo Potentiating Intestinal Inflammation,” Gut 66, no. 8 (2017): 1414–1427, 10.1136/gutjnl-2016-313099. 28325746 PMC5940336

[21] M. Valles‐Colomer , G. Falony , Y. Darzi , et al., “The Neuroactive Potential of the Human Gut Microbiota in Quality of Life and Depression,” Nature Microbiology 4, no. 4 (2019): 623–632, 10.1038/s41564-018-0337-x. 30718848

[22] S. Zhu , Y. Jiang , K. Xu , et al., “The Progress of Gut Microbiome Research Related to Brain Disorders,” Journal of Neuroinflammation 17, no. 1 (2020): 25, 10.1186/s12974-020-1705-z. 31952509 PMC6969442

[23] Y. Funai , K. Ichijo , S. Suzuki , et al., “Quantitative Analysis of Gastrointestinal Fluid Absorption and Secretion to Estimate Luminal Fluid Dynamics in Rats,” Scientific Reports 13, no. 1 (2023): 17454, 10.1038/s41598-023-44742-y. 37838772 PMC10576741

[24] B. Javdan , J. G. Lopez , P. Chankhamjon , et al., “Personalized Mapping of Drug Metabolism by the Human Gut Microbiome,” Cell 181, no. 7 (2020): 1661–1679, 10.1016/j.cell.2020.05.001. 32526207 PMC8591631

[25] U. N. Shivaji , O. M. Nardone , R. Cannatelli , S. C. Smith , S. Ghosh , and M. Iacucci , “Small Molecule Oral Targeted Therapies in Ulcerative Colitis,” The Lancet Gastroenterology & Hepatology 5, no. 9 (2020): 850–861, 10.1016/S2468-1253(19)30414-5. 32171056

[26] M. Shehab , F. Alrashed , A. Alsayegh , et al., “Comparative Efficacy of Biologics and Small Molecule in Ulcerative Colitis: A Systematic Review and Network Meta‐Analysis,” Clinical Gastroenterology and Hepatology 23, no. 2 (2025): 250–262, 10.1016/j.cgh.2024.07.033. 39182898

[27] S. Danese , M. F. Neurath , A. Kopon , et al., “Effects of Apremilast, an Oral Inhibitor of Phosphodiesterase 4, in a Randomized Trial of Patients with Active Ulcerative Colitis,” Clinical Gastroenterology and Hepatology 18, no. 11 (2020): 2526–2534, 10.1016/j.cgh.2019.12.032. 31926340

[28] S. Danese , M. F. Neurath , A. Kopon , et al., “Effects of Apremilast, an Oral Inhibitor of Phosphodiesterase 4, in a Randomized Trial of Patients with Active Ulcerative Colitis,” Clinical Gastroenterology and Hepatology 18, no. 11 (2020): 2526–2534.e9, 10.1016/j.cgh.2019.12.032. 31926340

[29] H. Li , Y. Zhang , M. Liu , et al., “Targeting PDE4 as a Promising Therapeutic Strategy in Chronic Ulcerative Colitis through Modulating Mucosal Homeostasis,” Acta Pharmaceutica Sinica B 12, no. 1 (2022): 228–245, 10.1016/j.apsb.2021.04.007. 35127382 PMC8799862

[30] A. Blokland , P. Heckman , T. Vanmierlo , R. Schreiber , D. Paes , and J. Prickaerts , “Phosphodiesterase Type 4 Inhibition in CNS Diseases,” Trends in Pharmacological Sciences 40, no. 12 (2019): 971–985, 10.1016/j.tips.2019.10.006. 31704172

[31] F. Yang , Y. Su , C. Yan , T. Chen , and P. C. K. Cheung , “Attenuation of Inflammatory Bowel Disease by Oral Administration of Mucoadhesive Polydopamine‐Coated Yeast β‐Glucan via ROS Scavenging and Gut Microbiota Regulation,” Journal of Nanobiotechnology 22, no. 1 (2024): 166, 10.1186/s12951-024-02434-3. 38610032 PMC11010398

[32] K.‐H. Chen , N. Nguyen , T.‐Y. Huang , et al., “Macrophage‐Hitchhiked Orally Administered β‐Glucans‐Functionalized Nanoparticles as “Precision‐Guided Stealth Missiles” for Targeted Pancreatic Cancer Therapy,” Advanced Materials 35, no. 40 (2023): 2304735, 10.1002/adma.202304735. 37363886

[33] F. Ahmad , S. Ahmad , T. K. Upadhyay , et al., “Rifabutin Loaded Inhalable β‐Glucan Microparticle Based Drug Delivery System for Pulmonary TB,” Scientific Reports 14, no. 1 (2024): 16437, 10.1038/s41598-024-66634-5. 39013991 PMC11253001

[34] K. Gao , C. Mu , A. Farzi , and W. Zhu , “Tryptophan Metabolism: A Link between the Gut Microbiota and Brain,” Advances in Nutrition 11, no. 3 (2020): 709–723, 10.1093/advances/nmz127. 31825083 PMC7231603

[35] L.‐M. Chen , C.‐H. Bao , Y. Wu , et al., “Tryptophan‐Kynurenine Metabolism: A Link between the Gut and Brain for Depression in Inflammatory Bowel Disease,” Journal of Neuroinflammation 18, no. 1 (2021): 135, 10.1186/s12974-021-02175-2. 34127024 PMC8204445

[36] H. M. Roager and T. R. Licht , “Microbial Tryptophan Catabolites in Health and Disease,” Nature Communications 9, no. 1 (2018): 3294, 10.1038/s41467-018-05470-4. PMC6098093 30120222

[37] G. Wang , S. Huang , Y. Wang , et al., “Bridging Intestinal Immunity and Gut Microbiota by Metabolites,” Cellular and Molecular Life Sciences 76, no. 20 (2019): 3917–3937, 10.1007/s00018-019-03190-6. 31250035 PMC6785585

[38] J. Zhao , Y. Sun , H. Yang , et al., “PLGA‐Microspheres‐Carried circGMCL1 Protects against Crohn's Colitis through Alleviating NLRP3 Inflammasome‐Induced Pyroptosis by Promoting Autophagy,” Cell Death & Disease 13, no. 9 (2022): 1–14, 10.1038/s41419-022-05226-5. PMC9464224 36088391

[39] A. Chaplin , H. Gao , C. Asase , et al., “Systemically‐Delivered Biodegradable PLGA Alters Gut Microbiota and Induces Transcriptomic Reprogramming in the Liver in an Obesity Mouse Model,” Scientific Reports 10, no. 1 (2020): 13786, 10.1038/s41598-020-69745-x. 32796856 PMC7429827

[40] Y. Li , B. Zhu , T. Chen , et al., “Glutathione‐Responsive Nanoplatforms Trigger Gaseous Intervention of Intestinal Inflammation through TLR4/MD2/MyD88/NF‐κB/iNOS Pathway Activation and Gut Microbiota Modulation,” Chemical Engineering Journal 493 (2024): 152849, 10.1016/j.cej.2024.152849.

[41] C. Jori , A. Ahmad , A. Kumar , et al., “Bioactive Chitosan‐BSA Maillard‐Derived Chrysin‐Loaded Nanoparticles: A Gastroprotective, Biomucoadhesive Approach for Enhanced Oral Therapy in Ulcerative Colitis,” Carbohydrate Polymers 359 (2025): 123537, 10.1016/j.carbpol.2025.123537. 40306769

[42] J. Ye , X. Zhang , W. Xie , et al., “An Enzyme‐Responsive Prodrug with Inflammation‐Triggered Therapeutic Drug Release Characteristics,” Macromolecular Bioscience 20, no. 9 (2020): 2000116, 10.1002/mabi.202000116. 32603032

[43] C. Medina , S. Videla , A. Radomski , et al., “Increased Activity and Expression of Matrix Metalloproteinase‐9 in a Rat Model of Distal Colitis,” American Journal of Physiology‐Gastrointestinal and Liver Physiology 284, no. 1 (2003): G116–G122, 10.1152/ajpheart.00036.2002. 12488238

[44] S. G. Nugent , D. Kumar , D. S. Rampton , and D. F. Evans , “Intestinal Luminal pH in Inflammatory Bowel Disease: Possible Determinants and Implications for Therapy with Aminosalicylates and Other Drugs,” Gut 48, no. 4 (2001): 571–577, 10.1136/gut.48.4.571. 11247905 PMC1728243

[45] C. N. Chew , D. J. Nolan , and D. P. Jewell , “Small Bowel Gas in Severe Ulcerative Colitis,” Gut 32, no. 12 (1991): 1535–1537, 10.1136/gut.32.12.1535. 1773962 PMC1379257

[46] U. Erben , C. Loddenkemper , K. Doerfel , et al., “A Guide to Histomorphological Evaluation of Intestinal Inflammation in Mouse Models,” International Journal of Clinical and Experimental Pathology 7, no. 8 (2014): 4557–4576. 25197329 PMC4152019

[47] K. N. Gibson‐Corley , A. K. Olivier , and D. K. Meyerholz , “Principles for Valid Histopathologic Scoring in Research,” Veterinary Pathology 50, no. 6 (2013): 1007–1015, 10.1177/0300985813485099. 23558974 PMC3795863

[48] J. A. Croix , F. Carbonero , G. M. Nava , M. Russell , E. Greenberg , and H. R. Gaskins , “On the Relationship between Sialomucin and Sulfomucin Expression and Hydrogenotrophic Microbes in the Human Colonic Mucosa,” PLoS ONE 6, no. 9 (2011): 24447, 10.1371/journal.pone.0024447. PMC3170330 21931721

[49] B. Deplancke and H. R. Gaskins , “Microbial Modulation of Innate Defense: Goblet Cells and the Intestinal Mucus Layer,” The American Journal of Clinical Nutrition 73, no. 6 (2001): 1131S–1141S, 10.1093/ajcn/73.6.1131S. 11393191

[50] A. C. C. C. Branco , F. S. Y. Yoshikawa , A. J. Pietrobon , and M. N. Sato , “Role of Histamine in Modulating the Immune Response and Inflammation,” Mediators of Inflammation 2018 (2018): 1–10, 10.1155/2018/9524075. PMC6129797 30224900

[51] P. K. Randhawa , K. Singh , N. Singh , and A. S. Jaggi , “A Review on Chemical‐Induced Inflammatory Bowel Disease Models in Rodents,” The Korean Journal of Physiology & Pharmacology 18, no. 4 (2014): 279–288, 10.4196/kjpp.2014.18.4.279. 25177159 PMC4146629

[52] G. Sharon , N. J. Cruz , D.‐W. Kang , et al., “Human Gut Microbiota from Autism Spectrum Disorder Promote Behavioral Symptoms in Mice,” Cell 177, no. 6 (2019): 1600–1618.e17, 10.1016/j.cell.2019.05.004. 31150625 PMC6993574

[53] M. Zheng , R. Han , Y. Yuan , et al., “The Role of Akkermansia Muciniphila in Inflammatory Bowel Disease: Current Knowledge and Perspectives,” Frontiers in Immunology 13 (2023): 1089600, 10.3389/fimmu.2022.1089600. 36685588 PMC9853388

[54] J. Xu , P. Li , Z. Li , et al., “Gut Bacterial Type III Secretion Systems Aggravate Colitis in Mice and Serve as Biomarkers of Crohn's Disease,” eBioMedicine 107 (2024): 105296, 10.1016/j.ebiom.2024.105296. 39216231 PMC11402190

[55] H. C. Mirsepasi‐Lauridsen , B. A. Vallance , K. A. Krogfelt , and A. M. Petersen , “Escherichia Coli Pathobionts Associated with Inflammatory Bowel Disease,” Clinical Microbiology Reviews 32, no. 2 (2019): e00060–e00118, 10.1128/CMR.00060-18. 30700431 PMC6431131

[56] M. E. Caetano‐Silva , L. Rund , M. Vailati‐Riboni , et al., “The Emergence of Inflammatory Microglia During Gut Inflammation is Not Affected by FFAR2 Expression in Intestinal Epithelial Cells or Peripheral Myeloid Cells,” Brain, Behavior, and Immunity 118 (2024): 423–436, 10.1016/j.bbi.2024.03.016. 38467381

[57] V. Singh , G. Lee , H. Son , et al., “Butyrate Producers, “The Sentinel of Gut”: Their Intestinal Significance with and Beyond Butyrate, and Prospective Use as Microbial Therapeutics,” Frontiers in Microbiology 13 (2023): 1103836, 10.3389/fmicb.2022.1103836. 36713166 PMC9877435

[58] H. Zhang , Y. Pan , Y. Jiang , et al., “Akkermansia Muciniphila ONE Effectively Ameliorates Dextran Sulfate Sodium (DSS)‐Induced Ulcerative Colitis in Mice,” npj Science of Food 8, no. 1 (2024): 97, 10.1038/s41538-024-00339-x. 39562574 PMC11576909

[59] S.‐K. Seo and B. Kwon , “Immune Regulation through Tryptophan Metabolism,” Experimental & Molecular Medicine 55, no. 7 (2023): 1371–1379, 10.1038/s12276-023-01028-7. 37394584 PMC10394086

[60] A. H. Gaike , S. D. Kalamkar , V. Gajjar , et al., “Effect of Long‐Term Oral Glutathione Supplementation on Gut Microbiome of Type 2 Diabetic Individuals,” FEMS Microbiology Letters 370 (2023): fnad116, 10.1093/femsle/fnad116. 37935462

[61] R. Abdel‐Haq , J. C. M. Schlachetzki , C. K. Glass , and S. K. Mazmanian , “Microbiome–Microglia Connections via the Gut–Brain Axis,” Journal of Experimental Medicine 216, no. 1 (2019): 41–59, 10.1084/jem.20180794. 30385457 PMC6314531

[62] H. Wang , J. S. Labus , F. Griffin , et al., “Functional Brain Rewiring and Altered Cortical Stability in Ulcerative Colitis,” Molecular Psychiatry 27, no. 3 (2022): 1792–1804, 10.1038/s41380-021-01421-6. 35046525 PMC9095465

[63] A. Shahini and A. Shahini , “Role of Interleukin‐6‐Mediated Inflammation in the Pathogenesis of Inflammatory Bowel Disease: Focus on the Available Therapeutic Approaches and Gut Microbiome,” Journal of Cell Communication and Signaling 17, no. 1 (2023): 55–74, 10.1007/s12079-022-00695-x. 36112307 PMC10030733

[64] A. S. Rehman , P. Kumar , and S. Parvez , “Dopamine‐D2‐Agonist Targets Mitochondrial Dysfunction via Diminishing Drp1 Mediated Fission and Normalizing PGC1‐α/SIRT3 Pathways in a Rodent Model of Subarachnoid Haemorrhage,” Neuroscience 564 (2025): 60–78, 10.1016/j.neuroscience.2024.11.028. 39542343

[65] N. Toledano Furman , A. Gottlieb , K. S. Prabhakara , et al., “High‐Resolution and Differential Analysis of Rat Microglial Markers in Traumatic Brain Injury: Conventional Flow Cytometric and Bioinformatics Analysis,” Scientific Reports 10, no. 1 (2020): 11991, 10.1038/s41598-020-68770-0. 32686718 PMC7371644

