# Supplementary material for: Targeted β‐Glucan‐Veiled Oral Apremilast Nanotherapy Modulates Key Dysbiosis‐Associated Gut Microbiota and Alleviates Ulcerative Colitis‐Associated Anxiety, Depression, and Neuropsychiatric Behaviors
Source: Adv Sci (Weinh). 2026 Jul 20;13(55):e76566. doi: 10.1002/advs.76566 (PMC13383883; doi:10.1002/advs.76566)
Supplement: Supplementary file 1 [file ADVS-13-e76566-s002.docx]

Supporting Information

**Targeted β-Glucan-Veiled Oral Apremilast Nanotherapy Modulates Key Dysbiosis-Associated Gut Microbiota and Alleviates Ulcerative Colitis-Associated Anxiety, Depression, and Neuropsychiatric Behaviors**

*Chandrashekhar Jori^1^, Ahmed Shaney Rehman^2^, Taruna Lamba^3^, Anas Ahmad^1^, Jattin Kumar^1^, Aneesh Ali^1^, Ajesh Joshi^1^, Ashraf Ali^1^, Suhel Parvez^2^, Javed N. Agrewala^3^,  Rehan Khan^1*^*

^1^Chemical Biology Unit, Institute of Nano Science and Technology, Sector 81, Knowledge city, Sahibzada Ajit Singh Nagar, Mohali, Punjab 140306, India.

^2^Department of Toxicology, School of Chemical and Life Sciences, Jamia Hamdard, New Delhi, 110 062, India.

^3^Immunology Laboratory, Department of Biomedical Engineering, Indian Institute of Technology, Ropar, Rupnagar, 140001, Punjab, India

***Corresponding author:**

Dr. Rehan Khan

Chemical Biology Unit, Institute of Nano Science and Technology (INST)

Knowledge City, Sector 81, S. A. S Nagar, Punjab - 140306

E-mail: rehankhan@inst.ac.in

Phone No. +91-172-2210075.

Contents

[1. Characterization techniques	5](#_Toc233206206)

[1.1 ^1^H Nuclear Magnetic Resonance (NMR)	5](#_Toc233206207)

[1.2 High-Resolution Mass Spectrometry (HRMS)	5](#_Toc233206208)

[1.3 Fourier Transform Infrared spectroscopy (FTIR)	5](#_Toc233206209)

[1.4 Hydrodynamic size of nanomicelles	5](#_Toc233206210)

[1.5 Zeta potential (ZETA SIZER) of nanomicelles	6](#_Toc233206211)

[1.6 Transmission Electron Microscopy (TEM) of nanomicelles	6](#_Toc233206212)

[1.7 Field Emission Scanning Electron Microscopy (FESEM) of nanomicelles	6](#_Toc233206213)

[1.8 Ultraviolet-visible (UV-Vis) spectroscopy	6](#_Toc233206214)

[1.9 Apremilast loading and release kinetics of nanomicelles	7](#_Toc233206215)

[1.10 Powder X-ray diffraction (PXRD)	7](#_Toc233206216)

[1.11 X-ray Photoelectron Spectroscopy (XPS)	8](#_Toc233206217)

[2. Cell Experiment	8](#_Toc233206218)

[2.1 Cytotoxicity Assay	8](#_Toc233206219)

[2.2 *In vitro* anti-inflammatory activity of βG@Apr-WPG NMs in LPS-stimulated RAW264.7 cells	8](#_Toc233206220)

[2.3 Experimental procedure	9](#_Toc233206221)

[2.4. Stability and morphological analysis of βG@Apr-WPG NMs in simulated gastrointestinal conditions	9](#_Toc233206222)

[2.5. TEER measurement for *in vitro* BBB co-culture model	9](#_Toc233206223)

[3. Evaluation of βG@Apr-WPG NMs in a DSS-induced colitis model	10](#_Toc233206224)

[3.1 Colitis model induction in mice	11](#_Toc233206225)

[3.2 Assessment of physical parameters	11](#_Toc233206226)

[3.3 Histological analysis	12](#_Toc233206227)

[3.4 Assessment of Hematoxylin and Eosin (H&E) staining	12](#_Toc233206228)

[3.5 Assessment of Alcian Blue and neutral red (AB-NR) staining	12](#_Toc233206229)

[3.6 Assessment of High Iron Diamine-Alcian Blue (HID- AB) staining	12](#_Toc233206230)

[3.7 Assessment of immunohistochemical staining	13](#_Toc233206231)

[3.8 Assessment of myeloperoxidase (MPO) activity	13](#_Toc233206232)

[3.9 Nitrite determine via the Griess reaction	13](#_Toc233206233)

[3.10 Protein content analysis	13](#_Toc233206234)

[3.11 Hemolysis assay	13](#_Toc233206235)

[4. Neurobehavioral tests in IBD mice treated with βG@Apr-WPG NMs	14](#_Toc233206236)

[4.1. Beam walk test	14](#_Toc233206237)

[4.2. Grip strength test	15](#_Toc233206238)

[4.3. Elevated Plus Maze (EPM) test	16](#_Toc233206239)

[4.4. Forced Swim Test (FST)	16](#_Toc233206240)

[4.5. Sucrose Preference Test (SPT)	16](#_Toc233206241)

[5.	Preparation of Single-Cell Suspensions from Gut-Associated Lymphoid Tissue (GALT)	17](#_Toc233206242)

[6.	Detailed protocol for brain dissociation, microglia enrichment, and flow cytometric analysis	18](#_Toc233206243)

[Figure S1: ^1^H NMR of PLGA-GSH (PG) conjugate.	19](#_Toc233206244)

[Figure S2: ^1^H NMR of Trp-PLGA-GSH (WPG) conjugate.	20](#_Toc233206245)

[Figure S3: ^1^H NMR Stacking spectra of Glutathione (G), PLGA-Glutathione (PG), and Tryptophan-PLGA-Glutathione (WPG) conjugate in DMSO-d6.	21](#_Toc233206246)

[Figure S4. HRMS assessment for Tryptophan–PLGA–Glutathione conjugate (ESI-QTOF, Positive Mode)	22](#_Toc233206247)

[Figure S5. FTIR analysis for functional group characterization	23](#_Toc233206248)

[Figure S6. XRD analysis of	24](#_Toc233206249)

[Figure S7. Calibration curve of apremilast at varying concentrations (μg/mL).	25](#_Toc233206250)

[Figure S8. Rheological characterization of βG@Apr-WPG nanomicelles (NMs).	26](#_Toc233206251)

[Figure S9. Concentration-dependent release of apremilast from βG@Apr-WPG nanomicelles (NMs) in response to esterase and MMP-9 at pH 7.4	27](#_Toc233206252)

[Figure S10. Establishment of an *in vitro* and *ex vivo* BBB co-culture model and evaluation of βG@Apr-WPG nanomicelles	28](#_Toc233206253)

[Figure S11. Assessment of rectal bleeding in experimental groups	29](#_Toc233206254)

[Figure S12. Bloating is a prominent clinical characteristic of ulcerative colitis.	30](#_Toc233206255)

[Figure S13. Quantitative assessment of colonic injury and mucin preservation.	31](#_Toc233206256)

[Figure S14. Mast cell activation in the submucosal layer of the colon in colitis and treatment groups.	32](#_Toc233206257)

[Figure S15. Hemolysis assay of βG@Apr-WPG NMs.	33](#_Toc233206258)

[Figure S16. βG@Apr-WPG NMs mitigate colitis-induced neuroinflammation.	34](#_Toc233206259)

[Figure S17. Grip strength assessment in experimental groups.	35](#_Toc233206260)

[Figure S18. Elevated plus maze (EPM) analysis of anxiety-like behavior in DSS-induced colitis mice	36](#_Toc233206261)

[Figure S19. Complete gating strategy employed in flow cytometry for acquisition and analysis of populations of various cell types	37](#_Toc233206262)

[Figure S20. Gating strategy for microglia identification	38](#_Toc233206263)

[Figure S21. Gating strategy for astrocytes identification	39](#_Toc233206264)

[Table S1. Storage stability study evaluating changes in hydrodynamic size (nm), Zeta potential (mV), and encapsulation efficiency under storage conditions.	43](#_Toc233206265)

[Table S2*.* Serum biomarkers for liver and kidney function were assessed to evaluate the safety profile of Blank nanomicelles (Control, and Control + βG@B-WPG NMs).	44](#_Toc233206266)

[Legends for Supporting video	44](#_Toc233206267)

[Supporting video file, SV 1: Assessment of physical activity in DSS-induced colitis mice (colitis model) and treated with βG@Apr-WPG NMs (DSS + βG@Apr-WPG NMs) (n = 3).	44](#_Toc233206268)

[Supporting video file, SV 2: Beam walk test to assess motor coordination in IBD mice treated with βG@Apr-WPG NMs for (A) Control (healthy), (B) DSS-induced colitis, (C) DSS + βG@Apr-WPG NMs (n = 3).	44](#_Toc233206269)

[Supporting video file, SV 3: Grip strength test: assessing neuromuscular function in UC mice treated with βG@Apr-WPG NMs for (A) Control (healthy), (B) DSS-induced colitis, (C) DSS + βG@Apr-WPG NMs (n = 3).	44](#_Toc233206270)

# 1. Characterization techniques

## 1.1 ^1^H Nuclear Magnetic Resonance (NMR)

The tryptophan–PLGA–glutathione (WPG) conjugated product was characterized by **¹H nuclear magnetic resonance (NMR) spectroscopy** using a **Bruker Avance-II spectrometer** operating at **400 MHz,** with **DMSO-d₆** as the solvent.

## 1.2 High-Resolution Mass Spectrometry (HRMS)

The structural confirmation of the tryptophan–PLGA–glutathione (WPG) conjugate was performed using high-resolution mass spectrometry (HRMS). Analysis was conducted on an Agilent 6500 Series Quadrupole Time-of-Flight (Q-TOF) mass spectrometer equipped with an electrospray ionization (ESI) source, operating in positive ion mode. Samples were dissolved in LC-MS grade methanol:water (1:1, v/v) and filtered through a 0.22 µm syringe filter prior to analysis. Data processing and peak identification were carried out using Agilent MassHunter Qualitative Analysis Software (version 10.0). The retention time and exact m/z values of the detected peaks were used to infer the presence of conjugated and fragment species corresponding to tryptophan, glutathione, and the PLGA backbone.

## 1.3 Fourier Transform Infrared spectroscopy (FTIR)

Fourier Transform Infrared (FTIR) spectra for Beta-glucan (βG), glutathione (G), PLGA (P), tryptophan (W), WPG NMs, apremilast (Apr), βG@Apr-WPG NMs (5 mg each) were recorded in order to determine which functional groups are included in the nanoformulation using a Cary Agilent 660 IR spectrophotometer. Each spectrum was obtained with 256 scans at a resolution of 4 cm⁻¹, covering a spectral range from 400 to 4000 cm⁻¹.

## 1.4 Hydrodynamic size of nanomicelles

The mean hydrodynamic diameter and polydispersity index (PDI) of nanomicelles, and zeta potential of different blank WPG NMs, and apremilast loaded βG@Apr-WPG NMs (n=3) were analyzed using dual-angle dynamic light scattering (DLS), referred as photon correlation spectroscopy, employing a DLS Zetasizer Nano ZSP (Model ZEN5600; Malvern Instruments Ltd., Worcestershire, UK). Each measurement was performed in triplicate, with the average of the three readings reported as the final particle size. All measurements were carried out at a controlled temperature of 25 °C.

## 1.5 Zeta potential (ZETA SIZER) of nanomicelles

Using palladium electrodes and the Phase Analysis Light Scattering (PALS) approach, the zeta potential of βG@Apr-WPG NMs (n=3) was detected in disposable folding capillary cells (Model DTS1070; Malvern) using a Zetasizer Nano ZSP (Model ZEN5600; Malvern Instruments Ltd., Malvern, UK). The final zeta potential value was determined by averaging three independent readings.

## 1.6 Transmission Electron Microscopy (TEM) of nanomicelles

For TEM analysis, a small drop of βG@Apr-WPG NMs and WPG NMs aqueous suspension was deposited onto a carbon-coated 300-mesh copper grid (Ted Pella Inc.) and allowed to incubate for 20–30 minutes to ensure adequate adsorption. The loaded grids were subsequently placed in a desiccator for overnight air drying. Imaging was analyzed using a JEOL JEM-2100 transmission electron microscope (Tokyo, Japan) equipped with a tungsten filament, operated at an accelerating voltage of 120 kV. TEM micrographs were captured and processed digitally using Gatan camera software.

## 1.7 Field Emission Scanning Electron Microscopy (FESEM) of nanomicelles

For Field Emission Scanning Electron Microscopy (FESEM) analysis, Milli-Q water was used to prepare the βG@Apr-WPG NMs and WPG NMs samples, and laid out a silicon wafer by drop casting, followed by overnight drying through slow evaporation. The morphology of the nanomicelles was examined using a JEOL JSM-7600F scanning electron microscope (Tokyo, Japan), operated at an accelerating voltage of 5.0–10.0 kV.

## 1.8 Ultraviolet-visible (UV-Vis) spectroscopy

A Shimadzu UV-2600 UV-Vis spectrophotometer was employed to establish the standard calibration curve of Apremilast (Apr) at its maximum absorbance wavelength (λmax = 270nm). This calibration facilitated the quantification of the drug loaded into βG@Apr-WPG NMs and subsequent determination of the nanomicelles loading capacity and drug encapsulation efficiency (n=3). Apremilast solutions at various concentrations were examined using a quartz cuvette with a path length of 1 cm.

## 1.9 Apremilast loading and release kinetics of nanomicelles

The apremilast loading capacity and encapsulation efficiency of βG@Apr-WPG NMs (n=3) were evaluated to confirm the efficacy of the nanomicelles as a drug delivery system for Apremilast. During the fabrication of WPG NMs, the apremilast was incorporated into the nanomicelles, followed by centrifugation to separate the formulations. The supernatant containing free, unencapsulated drug was collected and analyzed using a UV-visible spectrophotometer. Based on these measurements, the loading capacity of the nanomicelles and the encapsulation efficiency of the drug were calculated using the following equations:

**Loading capacity of WPG NMs (%, W/W) =**

$$\frac{Amount of apremilast loaded in WPG NMs}{The total quantity of WPG NMs employed}\times100$$

**Encapsulation efficiency of Apremilast (%, W/W) =**

$$\frac{The quantity of apremilast encapsulated in WPG NMs}{The total amount of WPG NMs employed}\times100$$

The release kinetics of the apremilast from βG@Apr-WPG NMs was investigated at 37 °C in phosphate-buffered saline (PBS) at pH 7.4 using the dialysis method with minor modifications ^[1]^. The study assessed the progressive release of the apremilast into PBS. βG@Apr-WPG NMs, lyophilized nanomicelles were suspended in 2 mL of PBS and placed inside a pre-activated dialysis pouch (Dialysis Membrane-70, 12–14 kDa molecular weight cutoffs, HiMedia Laboratories Pvt. Ltd., India) measuring 5 cm in length. The βG@Apr-WPG NMs loaded dialysis membrane was immersed in 100 mL of PBS under constant stirring at 100 rpm. At predefined time intervals (0.25, 0.5, 1, 2, 4, 8, 12, 24, 36, 48, 60, 72, 84, and 96 hours), To maintain sink conditions, 1 milliliter of receptor medium was removed, followed by swapped out for an equivalent volume of fresh PBS. The concentration of the apremilast released from the nanomicelles in every test sample was quantified using a UV-Vis spectrophotometer.

## 1.10 Powder X-ray diffraction (PXRD)

To investigate the crystal structure, X-ray diffraction (XRD) analysis of Beta-glucan (βG), glutathione (G), PLGA (P), tryptophan (W), WPG NMs, apremilast (Apr), βG@Apr-WPG NMs (approximate 5 mg each) was performed using a Bruker D8 Advance diffractometer. The measurements were conducted with Cu-Kα radiation (λ = 1.5406 Å) over a 2θ range of 10° to 70°, employing an acceleration voltage of 40 kV to ensure sufficient resolution and intensity for phase identification.

## 1.11 X-ray Photoelectron Spectroscopy (XPS)

The bonding configuration and surface elemental composition of the prepared samples of βG@Apr-WPG NMs (β-glucan coated) and Apr-WPG NMs (without β-glucan coated) were analyzed using a K-Alpha 1063 X-ray Photoelectron Spectroscopy (XPS) system. The measurements were conducted in an ultrahigh vacuum (UHV) chamber maintained at a pressure of 7 × 10⁻⁹ Torr, employing monochromatic Al-Kα radiation with a photon energy of 1486.6 eV.

# 2. Cell Experiment

RAW264.7 and HCT-116 cells were cultured in high-glucose Dulbecco’s Modified Eagle’s Medium (DMEM; Gibco) supplemented with 10% fetal bovine serum (FBS; Gibco) and 1% penicillin-streptomycin solution (Gibco) in a 37°C, 5% CO₂ incubator.

## 2.1 Cytotoxicity Assay

The cytotoxicity of βG@Apr-WPG NMs was evaluated in HCT-116 cells using the 3-(4,5-dimethylthiazol-2-yl)-2,5-diphenyltetrazolium bromide (MTT) assay. Briefly, HCT-116 cells were seeded at a density of 1 × 10^4^ cells/mL in 96-well plates and incubated until fully adherent. The culture medium was replaced with high-glucose DMEM containing varying concentrations of βG@Apr-WPG NMs (0, 3.125, 6.25, 12.5, 25, 50, 100, 200, 400, and 800 µg mL⁻¹). After 24 and 48 hours of incubation, 100 µL of MTT solution (5 mg mL⁻¹) was added to each well and incubated for 4 hours at 37°C. The medium was then removed, and 200 µL of dimethyl sulfoxide (DMSO) was added to dissolve the formazan crystals. The absorbance was measured at 590 nm using an ELISA microplate reader. Cell viability was calculated as a percentage relative to the control group (no NMs).

## ****2.2 *In vitro* anti-inflammatory activity of βG@Apr-WPG NMs in LPS-stimulated RAW264.7 cells****

The supernatant from cultured cells is collected to measure the levels of secreted inflammatory cytokines, which are released into the extracellular medium in response to stimuli such as lipopolysaccharide (LPS). These cytokines, including tumor necrosis factor-α (TNF-α), interleukin-6 (IL-6), and interleukin-10 (IL-10), serve as key markers of inflammatory responses. By analyzing the supernatant, researchers can quantify the anti-inflammatory effects of therapeutic agents, such as βG@Apr-WPG NMs, on immune cells like RAW264.7 macrophages.

## 2.3 Experimental procedure

RAW264.7 cells were seeded into 12-well culture plates at a density of 10⁷ cells per well. The cells were stimulated with LPS (500 ng mL⁻¹) to induce inflammation and treated with 200 µg mL⁻¹ of βG@Apr-WPG NMs. After 24 hours of incubation, the supernatant was collected and centrifuged to remove any cellular debris. The levels of TNF-α, IL-6, and IL-10 in the supernatant were quantified using enzyme-linked immunosorbent assay (ELISA) kits (eBioscience) according to the manufacturer’s protocol. This approach allowed for the evaluation of the anti-inflammatory efficacy of βG@Apr-WPG NMs by measuring their ability to modulate cytokine secretion in LPS-stimulated RAW264.7 cells.

## 2.4. Stability and morphological analysis of βG@Apr-WPG NMs in simulated gastrointestinal conditions

The stability of βG@Apr-WPG NMs under simulated gastrointestinal conditions was assessed using an adapted protocol. Briefly, 1 mL of βG@Apr-WPG NMs (10 mg mL⁻¹) was mixed with simulated gastric fluid (SGF; pH 2) consisted pepsin (10 mg mL⁻¹) in a 1:1 (v/v) ratio and incubated at 37°C for 2 hours. The mixture was then centrifuged at 10,000 rpm for 10 minutes, and the pellet was resuspended in simulated intestinal fluid (SIF; pH 6.8) for further incubation at room temperature for 4 hours. Post-incubation, the NM/SIF solution was centrifuged again at 10,000 rpm for 10 minutes. Subsequently, TEM and FESEM samples were prepared as described earlier to analyze the morphological integrity of the nanoparticles. This protocol evaluated the stability and structural resilience of βG@Apr-WPG NMs in conditions mimicking the gastrointestinal tract, offering essential insights into their suitability for oral therapeutic delivery.

## 2.5. TEER measurement for *in vitro* BBB co-culture model

An *in vitro* blood–brain barrier (BBB) model was established using a Transwell co-culture system comprising bEnd.3 mouse brain endothelial cells and U87 cells. U87 cells (1 × 10^5^ cells/well) were seeded in the basolateral compartment and cultured for 24 h. Subsequently, bEnd.3 cells (1 × 10^5^ cells/insert) were seeded onto collagen-coated Transwell inserts (0.4 μm pore size). Cells were maintained in DMEM supplemented with 10% FBS and 1% penicillin-streptomycin at 37°C under 5% CO₂. The co-culture was maintained for 4–5 days with medium replacement every 48 h until a stable endothelial monolayer was established, as confirmed by TEER measurements. Inflammatory BBB disruption was induced by treating the apical compartment with lipopolysaccharide (LPS, 1 μg mL⁻¹) for 12 h. Experimental groups included: Control, LPS, LPS + free apremilast, LPS + βG@B-WPG nanomicelles, and LPS + βG@Apr-WPG nanomicelles. Treatment formulations were added concurrently with LPS and incubated for 12 h.

# 3. Evaluation ****of**** β****G@Apr-WPG NMs in a DSS-induced colitis model****

The therapeutic effects of βG@Apr-WPG NMs were assessed using dextran sulfate sodium (DSS)-induced colitis model. A total of 48 mice were randomly allocated into six groups, each comprising eight animals, as outlined below.

- **Group 1 (Control, i.e. healthy):** The mice were fed a typical baseline diet without any treatment and served as control.
- **Group 2 (DSS colitic model):** For 10 days, mice were supplied 2.5% w/v DSS in drinking water ad libitum, (*ad.lib.*) in order to induce colitis.
- **Group 3 (DSS + βG@Apr-WPG NMs, Treatment group):** Colitis-induced mice were treated with **βG@Apr-WPG NMs** at an equivalent dose corresponding to 50 mg/kg body weight of apremilast (Apr), administered orally *(p.o.)* via gavage for 10 alternate days post-induction of colitis to evaluate the better therapeutic effect of **β**G@Apr-WPG NMs against the colitis.
- **Group 4 (DSS + Apremilast, Free Apr):** Colitis-induced mice received a 50 mg/kg body weight dose of free Apr, administered orally *(p.o.)* via gavage for 10 alternate days post-induction of colitis to evaluate the therapeutic effect of naïve apremilast against the colitis.
- **Group 5 (DSS + βG@B-WPG NMs, blank nanomicelles without Apr):** Colitis-induced mice, an equal dosage was administered orally *(p.o.)* of β**G@B-WPG NMs** via gavage for 10 alternate days to evaluate the efficacy of β**G@B-WPG NMs** in DSS-induced colitis mice model.
- **Group 6 (Control + βG@B-WPG NMs, Safety group):** Healthy mice were treated with an equal dosage administered orally *(p.o.)* of **βG@B-WPG NMs** via gavage for 10 alternate days for evaluation of the toxicity towards vital organs such as the heart and spleen, kidney, lungs and liver.

## 3.1 Colitis model induction in mice

Ulcerative colitis was induced in Swiss albino mice by administering 2.5 % w/v DSS in drinking water ad libitum (*ad.lib.*) for 10 days. Colonic inflammation was evaluated 10 days post-DSS treatment by assessing physical activity, stool consistency, and rectal haemorrhage, confirming the establishment of colitis.

## 3.2 Assessment of physical parameters

A disease activity index was established, considering factors like body weight loss (physical activity), blood in the stool via fecal occult blood test (FOBT)*,* and stool consistency to assess the severity of DSS-induced inflammation in mice. Colon shortening, indicative of severe inflammation, was evaluated as a significant indicator. Post-sacrifice assessment of colon length allowed for the evaluation of anti-inflammatory effects of both Apr and β**G@Apr-WPG NMs**, facilitating quantification of inflammation severity and assessment of therapeutic interventions.

The DAI provided a comprehensive measure of colitis progression and the therapeutic efficacy of βG@Apr-WPG NMs in reducing inflammation and improving clinical symptoms.

* Disease activity index (DAI) score used to evaluate the DSS-induced colitis

## 3.3 Histological analysis

To preserve cellular structure and morphology, the colons and vital organs, including the heart, kidneys, lungs, spleen, and liver, were promptly immersed in 10% neutral buffered formalin for 24 hours following euthanasia. Following fixation, the tissues were embedded in paraffin and sectioned into thin slices (5 μm thick) using a microtome. These sections were then mounted onto glass slides and subjected to Hematoxylin and Eosin (H&E) staining and other without any stain. The stained sections were subsequently examined under a light microscope for histological damages such as compromised integrity of the mucosal epithelial lining, partly erosion of colonic epithelial layer or lamina propria, crypt ablation, interstitial edema, and heavy infiltration of inflammatory cells.

## 3.4 Assessment of Hematoxylin and Eosin (H&E) staining

To visualize the tissue architecture, cell morphology, and pathological changes like epithelial erosion, crypt abscesses, and inflammatory infiltration in ulcerative colitis, tissue sections (5 μm thick) underwent deparaffinization in xylene, followed by alcohol and water hydration. H&E staining was performed, and the sections were mounted with DPX for histological analysis under an Olympus microscope at 20x and 40x magnifications ^[1]^.

## 3.5 Assessment of Alcian Blue and neutral red (AB-NR) staining

To assess the goblet cell depletion and mucosal inflammation, histological sections of the distal colon (5 μm thick) were deparaffinized with xylene and rehydrated with alcohol. Slides were treated with 1% Alcian blue solution (pH 2.5) to prevent non-specific staining, followed by rinsing with 3% acetic acid. Counterstaining with neutral red, dehydration, and mounting with DPX were performed for microscopic examination ^[1]^.

## 3.6 Assessment of High Iron Diamine-Alcian Blue (HID- AB) staining

To intrigue insights into goblet cell depletion, shifts in mucin types, and the impact of inflammation on mucosal integrity, distal colonic 5 μm tissue sections underwent deparaffinization through heating and xylene treatment, followed by dehydration using alcohol-water solutions (30%, 50%, 70%, and 100% alcohol). Sections were immersed in a high iron diamine (HID) solution for 18 to 22 hours, rinsed, and stained with a 1% alcian blue solution for 30 minutes. Slides were cleaned with xylene, rehydrated with alcohol, and mounted for subsequent analysis ^[1]^.

## 3.7 Assessment of immunohistochemical staining

Immunohistochemistry for specific biomarkers, five μm colonic tissue sections were paraffin-embedded and assessed TLR4, STING, IRF3, CREB, PDE4, NF-κB, NLRP3, CD8, AMPK, MUC2, Occludin, TJP, Claudin, and ZO1 expression. Sections were deparaffinized in xylene, rehydrated in graded ethanol, and subjected to antigen retrieval in 10 M citrate buffer (pH 6.0). Endogenous peroxidase activity was neutralized, and sections were incubated with 1% BSA before overnight exposure to rabbit polyclonal CREB, IRF3, TLR4, NF-kB p65, PDE4, PKA, STING, TJP, Claudin, Occludin, and ZO-1 primary antibodies at 4°C. After washing, sections were incubated with an HRP-conjugated secondary antibody, counterstained with hematoxylin, and mounted with DPX. Observations were made using a 40X magnification Olympus bright-field microscope ^[1]^.

## 3.8 Assessment of myeloperoxidase (MPO) activity

The activity of myeloperoxidase (MPO) was assessed as a marker of neutrophil infiltration in mouse colonic tissue. This measurement was conducted following a previously validated protocol. MPO activity was determined and reported in units per gram of protein (U/g) ^[1]^.

## 3.9 Nitrite determine via the Griess reaction

Colon tissues collected from euthanized animals were carefully rinsed with phosphate-buffered saline (PBS, pH 7.4) and maintained on ice, adhering to previously described methodologies ^[2,3]^. A 50 µl sample aliquot was combined with 100 µl of Griess reagent, and the mixture was incubated at room temperature for 5–10 minutes, protected from light. Absorbance was recorded at 540 nm using a microplate reader, following the manufacturer’s guidelines. Nitrite levels were quantified by comparing the absorbance readings to a standard curve prepared with sodium nitrite in the same buffer used for tissue homogenization ^[1]^.

## 3.10 Protein content analysis

Protein concentration was determined using the Bradford assay, with bovine serum albumin (BSA) serving as the standard, following a previously described protocol with slight modifications ^[1]^.

## 3.11 Hemolysis assay

The hemocompatibility of the βG@Apr-WPG nanomicelles was assessed on healthy BALB/c mice erythrocytes. The blood was collected in a tube containing EDTA and erythrocytes were isolated by centrifuging the blood (1000 × g, 10 min) and washing three times with PBS (pH 7.4). A 2% (v/v) RBC suspension was made in PBS. The dispersion of βG@Apr-WPG nanomicelles in PBS was done at 10, 25, 50, 100, 200 and 400 μg mL⁻¹. Equivalent amounts (500 μL) of the erythrocyte suspension and nanomicelle samples were mixed and then incubated at 37°C for 2 h. The negative and positive controls were PBS and 0.1% Triton X-100, respectively. After incubation, samples were centrifuged (1000×g, 10 minutes) and absorbance of the supernatants was read at 540 nm. The percentage hemolysis was determined with the positive controls (PBS) and negative controls (Triton X-100). Each experiment was conducted three times (n = 3) and the data are shown as mean ± SD. The hemolysis values of less than 5% were considered as acceptable hemocompatibility.

# 4. Neurobehavioral tests ****in IBD mice treated with βG@Apr-WPG NMs****

## ****4.1. Beam walk test****

The balance beam test is a widely used behavioral assay to evaluate motor coordination and balance in animals. In this study, the test was employed to assess the impact of inflammatory bowel disease (IBD) on complex motor movements and coordination in mice, as well as the therapeutic effects of βG@Apr-WPG NMs.

#### Experimental procedure

Each experimental group of mice underwent five trials in a beam-walking task using a cylindrical beam measuring 160 cm in length and 2.5 cm in diameter. The beam was elevated above the ground to create a challenging motor coordination task. The mean performance was calculated based on three selected trials within the testing period.

At the beginning of each trial, the mouse was placed at the designated starting position on the beam (2.5 × 160 cm). The trial was considered complete once the animal successfully traversed a distance of 1 meter. If the mouse failed to reach the 1-meter mark within 60 seconds, the trial was terminated, and the traversal time was recorded as 60 seconds.

To assess balance and motor coordination, each mouse was positioned on the narrow, 2 cm-wide beam at the start of the trial. The total duration the mouse maintained its balance on the beam within the 60-second timeframe was recorded. A scoring system was used to quantify balance performance, with a maximum score of 5 awarded if the mouse remained on the beam for the entire 60 seconds. The score was incrementally assigned, with one point given for every 12-second interval the animal maintained its position.

In addition to scoring balance duration, two key parameters fall frequency and total beam traversal time were recorded as indicators of vestibulomotor function. Mice that fell before completing the 1-meter distance were noted, and their time was registered accordingly.

## ****4.2. Grip strength test****

The grip strength test is a behavioral assay used to evaluate neuromuscular function and overall physical strength in mice. The apparatus consists of a 50 cm long string tightly stretched between two vertical supports, elevated 40 cm above a flat surface. Mice are placed midway on the string and assessed based on their ability to hold onto or traverse the string, scored on a six-point scale:

1. **0 = Fall off**
2. **1 = Hangs onto string by two forepaws**
3. **2 = Attempts to climb the string**
4. **3 = Hangs onto string by two forepaws and one or both hind paws**
5. **4 = Hangs onto string by all forepaws with tail wrapped around the string**
6. **5 = Escapes**

In the context of ulcerative colitis (UC), this test is particularly significant as UC is associated with systemic inflammation, which can lead to muscle weakness, fatigue, and neuromuscular dysfunction. Chronic inflammation and the release of pro-inflammatory cytokines, such as TNF-α and IL-6, can impair muscle function and coordination, contributing to reduced grip strength. Additionally, UC-related malnutrition, stress, and neuroinflammation may further exacerbate these physical impairments.

The grip strength test provides a quantitative measure of the systemic effects of UC and the efficacy of therapeutic interventions, such as βG@Apr-WPG NMs, in restoring neuromuscular function. Improvements in grip strength following treatment reflect not only the alleviation of systemic inflammation but also the potential of the therapy to enhance overall physical health and quality of life in UC patients. This test, therefore, serves as a valuable tool for evaluating the broader physiological impact of UC and the therapeutic potential of the treatments.

## 4.3. Elevated Plus Maze (EPM) test

Anxiety-like behavior was assessed using the Elevated Plus Maze (EPM), consisting of two open arms and two enclosed arms arranged in a plus-shaped configuration and elevated 50 cm above the floor. The open arms measured 35 cm in length and 5 cm in width, whereas the closed arms were surrounded by 15 cm high opaque walls. At the beginning of the experiment, each mouse was placed in the central platform facing an open arm and allowed to freely explore the maze for 5 min. Anxiety-related behavior was evaluated by quantifying the time spent in the open and closed arms, as well as the frequency of entries into the open arms. Increased exploration of the open arms was interpreted as reduced anxiety-like behavior.

## 4.4. Forced Swim Test (FST)

Depression-like behavior was evaluated using the Forced Swim Test (FST). Briefly, mice were individually placed in a transparent cylindrical container (10 cm diameter) filled with water (25 ± 1°C) to a depth of 30 cm, preventing the animals from touching the bottom with their tails or hind limbs. Each mouse was subjected to a 6-min test session. The duration of immobility was quantified as the time during which the animal remained floating passively, making only the minimal movements necessary to keep its head above the water surface. Increased immobility time was considered indicative of depression-like behavior, whereas reduced immobility following treatment was interpreted as an antidepressant-like effect.

## 4.5. Sucrose Preference Test (SPT)

Anhedonia-like behavior was evaluated using the sucrose preference test. Mice were acclimatized to a 1% (w/v) sucrose solution prior to testing. Following the habituation period, animals were provided with two pre-weighed bottles containing either sucrose solution or drinking water for 24 h. The volumes consumed from each bottle were recorded, and sucrose preference was calculated using the following equation:

Sucrose Preference (%) = [Sucrose Intake / (Sucrose Intake + Water Intake)] × 100

A reduction in sucrose preference was considered indicative of anhedonia-like behavior, whereas restoration of sucrose consumption reflected improvement in reward-seeking behavior.

# 5. Preparation of Single-Cell Suspensions from Gut-Associated Lymphoid Tissue (GALT)

In detail, it involved the mechanical dissection of colonic tissue with enzymatic digestion to release and isolate multiple types of single intestinal cells from colon tissue layers. The colons were isolated from mice, washed with ice-cold PBS (pH 7.4) and placed in a 50 mL tube containing 20 mL of pre-digestion media (Dulbecco's Modified Eagle's Medium (DMEM) with 10% fetal bovine serum). The falcon tubes were in an incubator shaker (with vigorous shaking at 250 rpm) at 37℃ for 20 minutes. After the first round of pre-digestion, the tubes were vortexed vigorously for 10 seconds and filtered through the 70 μm strainers, where the filtrate contained the IELs. The pre-digestion stem was carried out 3 times, vortexed and filtered every time to collect IEL cells. To isolate the LP cells, the tissues were digested with 20 mL complete media (DMEM, with 10% FBS) containing 150 units per mL of collagenase enzyme in the same incubator shaker (250 rpm, 20 minutes at 37 ℃). The digested tissue was resuspended in cell suspension buffer (PBS, pH 7.4 with 0.25% BSA and 2% FBS). Both LP and IEL cell suspensions were centrifuged at 1250 rpm for 8 minutes, supernatants were discarded, cell pellets were resuspended in 5 mL complete media (DMEM + 10% FBS), and the single-cell suspension was placed in a 5 mL sterile FACS tube for staining and acquisition. Cells were stained with an Antibody cocktail (50 μL each FACS tube) containing cell viability dye and cell surface marker antibodies for CD11b, CD11c, CD4+, CD8+, Ly6G, CD3+, CD19+, MHC-class II and Siglec-F etc after staining cells were washed once with 500 μL of complete media and fixed with fixation buffer (220 μL each FACS tube) for acquisition on BD FACS-Aria III flow cytometer. Live cells were gated for single cells (as shown in the SI.2), followed by gating the CD11c high and CD11c low cells. Macrophages and dendritic cells were gated out of the CD11c high gate, while B cells, T-cells, and neutrophils were gated out of the Cd11c low gate with their respective antibody markers, CD11c-APC, MHC-II-FITC, SiglecF-PerCP eFluor710, Ly6G-PE, CD8-BV786 and CD4-BV605 were used as single color controls for setting the instrumental compensations.

# 6. Detailed protocol for brain dissociation, microglia enrichment, and flow cytometric analysis

**Tissue processing and cell isolation**

Mice were anesthetized with sacrificed via right atrial puncture. Brains were extracted, neural cells were dissociated using the Neural Tissue Dissociation Kit (Miltenyi Biotec) with adjustments for mice tissue. Each brain was processed in a GentleMACS C-tube with 4 ml Buffer X and 100 µl Enzyme P, mechanically dissociated (program m_brain_01), incubated 15 min at 37 °C, followed by m_brain_02 with 40 µl Buffer Y and 20 µl Enzyme A for 10 min, and a final digestion step (m_brain_03) under identical conditions. Cell suspensions were filtered (70 μm), centrifuged, and washed with HBSS.

**Myelin removal and microglia enrichment**

Cells were suspended in 30% Percoll in HBSS, centrifuged (700 × g, 10 min, no brake). The myelin layer was discarded, and cells washed to remove Percoll (500 × g, 10 min). The resulting cell pellet was used for CD11b/c magnetic sorting (Miltenyi Biotec) to enrich microglia per manufacturer instructions. Enriched and non-enriched cells were maintained overnight in microglia-specific medium (ScienCell Research Labs).

**Flow cytometry and analysis**

CD11b/c-enriched cells were stained with antibodies per a previously validated multicolor panel ^[4]^ . After washing and dilution, cells were divided for M1/M2 phenotyping and incubated with specific antibody cocktails for 30 min at room temperature in the dark. Following a second wash, secondary antibodies and a live/dead dye (Ghost reagent) were added for 20 min. Cells were resuspended with counting beads (Cyto-Cal™) and analyzed using an LSRII cytometer (BD Biosciences). Data acquisition used Diva software; compensation was performed with VersaComp beads (Beckman Coulter).

**Characterization of Trp-PLGA-GSH (WPG) conjugate**

# Figure S1: ^1^H NMR of PLGA-GSH (PG) conjugate.

**Step 1 Synthesis of PLGA-GSH:**

PG characterised as, (400 MHz, DMSO-*d*_6_) δ 10.88 (s, 2H)CO-NH, 8.50 (s, 1H), 8.39 (s, 1H), 8.33 (s, 1H, CO-NH), 7.90 (s, 1H), 7.50 (d, *J* = 10.4 Hz, 1H), 7.33 (s, 2H), 7.10 (d, *J* = 29.0 Hz, 3H), 6.97 (s, 1H), 5.23 (d, *J* = 20.4 Hz, 22H), 5.00 – 4.63 (m, 32H), 4.54 (d, *J* = 9.4 Hz, 12H), 4.22 (d, *J* = 11.8 Hz, 3H), 4.18 – 3.97 (m, 11H), 2.94 (s, 2H), 1.70 (s, 13H), 1.24 (s, 2H).

# Figure S2: ^1^H NMR of Trp-PLGA-GSH (WPG) conjugate.

**Step 2 Synthesis of Trp- PLGA-GSH (WPG):**

WPG characterised as, 400 MHz, DMSO-*d*_6_) δ 10.88 (s, 2HCO-NH), 8.50 (s, 1H), 8.39 (s, 1H), 8.33 (s, 1HCO-NH), 7.90 (s, 1H), 7.50 (d, *J* = 10.4 Hz, 1H), 7.33 (s, 2H), 7.10 (d, *J* = 29.0 Hz, 3H), 6.97 (s, 1H), 5.23 (d, *J* = 20.4 Hz, 22H), 5.00 – 4.63 (m, 32H), 4.54 (d, *J* = 9.4 Hz, 12H), 4.22 (d, *J* = 11.8 Hz, 3H), 4.18 – 3.97 (m, 11H), 2.94 (s, 2H), 1.70 (s, 13H), 1.24 (s, 2H).

# Figure S3: ****^1^H NMR Stacking spectra of Glutathione (G), PLGA-Glutathione (PG), and Tryptophan-PLGA-Glutathione (WPG) conjugate in DMSO-d6.****

# Figure S4. HRMS assessment for Tryptophan–PLGA–Glutathione conjugate (ESI-QTOF, Positive Mode)

Figure S5. FTIR analysis for functional group characterization Beta-glucan (βG), glutathione (G), PLGA (P), tryptophan (W), WPG NMs, apremilast (Apr), βG@Apr-WPG NMs. FTIR analysis confirmed the successful synthesis of the PLGA–Glutathione–Tryptophan nanocarrier, β-glucan coating, and Apremilast loading. PLGA showed characteristic peaks at ~1750 cm⁻¹ (C=O stretching), 1180–1260 cm⁻¹ (C–O–C), and 2940–2880 cm⁻¹ (C–H). Glutathione displayed broad O–H/N–H stretching (~3300 cm⁻¹), amide I and II bands (~1650 and ~1540 cm⁻¹), and a weak S–H stretch (~2550 cm⁻¹), which disappeared upon conjugation. Tryptophan exhibited N–H (~3350 cm⁻¹), aromatic C–H (~3100 cm⁻¹), and indole ring vibrations (~1515–1590 cm⁻¹). Conjugation of PLGA with GSH and tryptophan was confirmed by the appearance of new amide peaks (~1650 and ~1540 cm⁻¹), C–N stretching (~1250 cm⁻¹), and the shift of C=O from ~1750 to ~1725 cm⁻¹. Apremilast showed peaks at ~1730 cm⁻¹ (ester C=O), ~1650 cm⁻¹ (amide), and ~1600 cm⁻¹ (aromatic C=C). β-glucan coating was confirmed by a broad O–H band (~3400 cm⁻¹), glycosidic C–O–C (~1155 cm⁻¹), and a β-linkage peak (~890 cm⁻¹). The final nanocarrier exhibited all characteristic peaks with broadened and slightly shifted bands, indicating successful conjugation, drug loading, and surface coating.

Figure S6. XRD analysis of Beta-glucan (βG), glutathione (G), PLGA (P), tryptophan (W), WPG NMs, apremilast (Apr), βG@Apr-WPG NMs.

# Figure S7. ****Calibration curve of apremilast at varying concentrations (μg/mL).****

Figure S8. Rheological characterization of βG@Apr-WPG nanomicelles (NMs). (A) Amplitude sweep showing the storage modulus (G′) and loss modulus (G″) as a function of shear strain (0.01–100%). The linear viscoelastic region (LVR) is identified by a plateau in G′ (~30 Pa), indicating stable nanostructure under small deformations. (B) Frequency sweep illustrating G′ and G″ across an angular frequency range of 0.1–100 rad/s. The dominance of G′ over G″ throughout the frequency range confirms elastic, solid-like behavior of the nanoformulation. These profiles highlight the structural integrity and viscoelastic stability of βG@Apr-WPG NMs under dynamic conditions.

Figure S9. Concentration-dependent release of apremilast from βG@Apr-WPG nanomicelles (NMs) in response to esterase and MMP-9 at pH 7.4**.** The release profile of apremilast was evaluated under physiological pH (7.4) in the presence of increasing concentrations of (A) esterase and (B) matrix metalloproteinase-9 (MMP-9). Data demonstrate a dose-dependent increase in apremilast release, indicating enzymatic responsiveness of the βG@Apr-WPG NMs. Results are presented as mean ± SD (n = 3).

Figure S10. Establishment of an *in vitro* and *ex vivo* BBB co-culture model and evaluation of βG@Apr-WPG nanomicelles. **(A)** Schematic illustration of the BBB co-culture system comprising bEnd.3 brain endothelial cells cultured on Transwell inserts and U87 cells seeded in the basolateral compartment. BBB integrity was evaluated using transendothelial electrical resistance (TEER) measurements. LPS stimulation induced BBB disruption, whereas treatment with βG@Apr-WPG nanomicelles promoted barrier restoration and reduced permeability. **(B)** TEER measurements demonstrating the preservation of BBB integrity following βG@Apr-WPG nanomicelle treatment compared with free apremilast and βG@B-WPG nanomicelles. Data are presented as mean ± SD (n = 3). **(C)** *Ex vivo* fluorescence imaging of isolated brain and colon tissues following administration of ICG-labeled nanomicelles. Negligible fluorescence signals were detected in brain tissues, whereas strong fluorescence accumulation was observed in the colon, indicating preferential localization of βG@ICG-WPG nanomicelles at inflamed intestinal sites with minimal BBB penetration. These findings suggest that the therapeutic effects on neuroinflammation are unlikely to result from direct brain accumulation and are more consistent with modulation of the microbiota–gut–brain axis.

Figure S11. Assessment of rectal bleeding in experimental groups**.** Rectal bleeding was evaluated as part of the Disease Activity Index (DAI) to assess the severity of colitis in different experimental groups. Representative images show the presence or absence of blood in fecal samples from (A) Control (healthy), (B) DSS-induced colitis, (bleeding was observed, red dotted circle) (C) DSS + βG@Apr-WPG NMs (no bleeding observed, green dotted circle), (D) DSS + free apremilast, (E) DSS + βG@B-WPG NMs, and (F) Control + βG@B-WPG NMs groups. Rectal bleeding scores were quantified and correlated with the progression of colitis and the therapeutic efficacy of βG@Apr-WPG NMs. Data are presented as mean ± SEM (n = 8).

Figure S12. Bloating is a prominent clinical characteristic of ulcerative colitis. (A) Physical examination showing evident abdominal bloating in the 2.5% DSS-induced colitis model. (B) Treatment with βG@Apr-WPG NMs effectively restored both bloating and colon morphology in the treated group (n = 8).

Figure S13. Quantitative assessment of colonic injury and mucin preservation. **(A)** Histopathological injury score derived from H&E-stained colon sections. **(B)** Semi-quantitative analysis of mucin content based on AB-NR staining. **(C)** Semi-quantitative analysis of sulfomucin abundance determined from HID-AB staining. Data are expressed as mean ± SD (n = 3); statistical significance is denoted as #P < 0.05, ##P < 0.01, ###P < 0.001, ####P < 0.0001 versus Control group; *P < 0.05, **P < 0.01, ***P < 0.001, ****P < 0.0001 versus DSS group, ns - non-significant.

Figure S14. Mast cell activation in the submucosal layer of the colon in colitis and treatment groups. Histological analysis shows prominent activation of mast cells (red arrow) in the submucosal layer of the colon in colitis-induced mice. In contrast, mast cells were absent in healthy controls, βG@B-WPG NMs-treated, and βG@Apr-WPG NMs-treated groups, indicating effective suppression of inflammation.

Figure S15. Hemolysis assay of βG@Apr-WPG NMs. Mouse erythrocytes were incubated with βG@Apr-WPG nanomicelles (10–400 μg mL⁻¹) for 2 h at 37°C. PBS and 0.1% Triton X-100 were used as negative and positive controls, respectively. The results of quantitative analysis showed that the hemolysis of βG@Apr-WPG nanomicelles was less than 5% at all concentrations tested, demonstrating excellent hemocompatibility. Data was presented as Mean ± SD (n = 3).

Figure S16. βG@Apr-WPG NMs mitigate colitis-induced neuroinflammation. Immunohistochemical staining of (A) pro-inflammatory cytokines (TNF-α, IL-1β, IL-18), (B) macrophage polarization factors (NF-κB), (C) oxidative stress markers (iNOS, COX-2), and (D) tissue integrity factors (fibronectin (FN), Beclin, Occludin, Claudin) in the dentate gyrus (DG) and cortex regions. The images demonstrate the ability of βG@Apr-WPG NMs to reduce neuroinflammation, oxidative stress, and tissue damage while restoring blood-brain barrier (BBB) integrity in DSS-induced colitis mice. (A) Control (healthy), (B) DSS-induced colitis, (C) DSS + βG@Apr-WPG NMs, (D) DSS + free apremilast, (E) DSS + βG@B-WPG NMs, and (F) Control + βG@B-WPG NMs. Scale bar = 50 µm.

Figure S17. Grip strength assessment in experimental groups. Grip strength was evaluated to assess neuromuscular function in mice from different experimental groups: (A) Control (healthy), (B) DSS-induced colitis, (C) DSS + βG@Apr-WPG NMs, (D) DSS + free apremilast, (E) DSS + βG@B-WPG NMs, and (F) Control + βG@B-WPG NMs. The test was performed using a wire grid, and the ability of mice to hold onto the grid was scored on a six-point scale (0 = Fall off; 1 = Hangs onto string by two forepaws; 2 = Attempts to climb the string; 3 = Hangs onto string by two forepaws and one or both hind paws; 4 = Hangs onto string by all forepaws with tail wrapped around the string; 5 = Escapes.). Data represent the mean ± SEM (n = 8) and demonstrate the therapeutic efficacy of βG@Apr-WPG NMs in restoring neuromuscular function in DSS-induced colitis mice.

Figure S18. Elevated plus maze (EPM) analysis of anxiety-like behavior in DSS-induced colitis mice. **(A)** Representative EPM tracking patterns and quantification of open-arm entries before and after treatment. **(B)** Representative heatmaps showing mouse movement during the 5 min EPM test. DSS-treated mice displayed reduced open-arm exploration, whereas βG@Apr-WPG NMs treatment restored exploratory behavior. Data are expressed as mean ± SD (n = 8); statistical significance is denoted as #P < 0.05, ##P < 0.01, ###P < 0.001, ####P < 0.0001 versus Control group; *P < 0.05, **P < 0.01, ***P < 0.001, ****P < 0.0001 versus DSS group, ns - non-significant.

Figure S19. Complete gating strategy employed in flow cytometry for acquisition and analysis of populations of various cell types. Among the CD11c-APC high cells, macrophages are taken as MHC II-FITC mid to high and (vs) Siglec-FPerCPeFluor 710-high population, while the dendritic cells are taken as MHC II-FITC high and (vs) SiglecF F PerCPeFluor 710-low population. Among CD11c-APC low cell populations, T cells are plotted as CD19/CD3e – APCCy7 high and MHC II – FITC low cells while B cells are plotted as CD19/CD3e – APCCy7 high and MHC II – FITC high cells. Furthermore, among the T cells, CD4+ cells are taken as CD4 – BV605 positive andCD8 – BV786 negative cells, while CD8+ cells are plotted as CD8 – BV786 positive and CD4 – BV605 negative cells. CD19/CD3e – APCCy7 low cells were further sub-gated and neutrophils were plotted as CD11b-PECy7 high, Ly6G-PE high cell populations.

Figure S20. Gating strategy for microglia identification: To identify microglial cells, a specific gating strategy was applied. Initially, the primary cell population was selected; debris and cell doublets were eliminated by analyzing forward scatter (FSC) area versus height to ensure single-cell events. Viable cells were then gated by excluding those stained with the Ghost™ viability dye. For comparative analysis, microglia was defined as cells expressing CD45, CD11b/c, and P2Y12 surface markers (CD45⁺CD11b/c⁺P2Y12⁺).

Figure S21. Gating strategy for astrocytes identification: Representative flow cytometry plots illustrating the sequential gating strategy used to identify astrocyte populations. Initial gating excluded debris and doublets based on forward and side scatter (FSC/SSC) properties. Singlets were then gated using FSC-H vs. FSC-A. Viable cells were selected using a viability dye exclusion method. Astrocytes were identified by positive expression of GFAP and/or other relevant markers. Fluorescence minus one (FMO) controls and isotype controls were used to define gating thresholds. This strategy was applied uniformly across experimental groups for consistent quantification.

 Figure S22. Food intake in IBD mice after gavage administration of βG@Apr-WPG NMs. Food intake was monitored daily for 10 days (after the treatment started ) in DSS-induced colitis mice treated with βG@Apr-WPG NMs, free apremilast, or blank nanomicelles (βG@B-WPG NMs). The graph illustrates the average food consumption (g/day) across experimental groups: (A) Control (healthy), (B) DSS-induced colitis, (C) DSS + βG@Apr-WPG NMs, (D) DSS + free apremilast, (E) DSS + βG@B-WPG NMs, and (F) Control + βG@B-WPG NMs. Data represent mean ± SEM (n = 8) and demonstrate the impact of βG@Apr-WPG NMs on improving appetite and nutritional status in colitis mice.

 **Supporting scheme 1 (SS 1). Schematic illustration of the mechanism of action of βG@Apr-WPG NMs in the experimental colitis model.** **(A)** Beta-glucan (βG) exhibits immunoregulatory effects in inflammatory bowel disease (IBD). **(B)** βG modulates tryptophan metabolism via the AHR-IL22 pathway, enhancing anti-inflammatory cytokine expression. **(C)** Apremilast (Apr) inhibits PDE4, attenuating inflammation by suppressing M1 and T cell activation. Elevated cAMP levels activate PKA, leading to CREB phosphorylation (increasing anti-inflammatory cytokines) and NF-κB phosphorylation (reducing pro-inflammatory cytokines). **(D)** βG@Apr-WPG NMs regulates the gut microbiome by enriching *Akkermansia muciniphila* and Firmicutes, outcompeting pathogenic *Escherichia coli* and *Shigella*. **(E)** Serotonin (5-HT), a key neurotransmitter, influences autonomic function, cognition, and emotional processes. Neurocognitive disorders like anxiety and depression are linked to reduced 5-HT and acetylcholine levels. βG@Apr-WPG NMs alleviate colitis symptoms and improve neurocognition via the gut-brain axis by enhancing melatonin and 5-HT production in enterochromaffin (EC) and neuronal cells, respectively. Created with Biorender.com.

| **Days** | **Hydrodynamic size (nm)** | | **Zeta potential (mV)** | | **Encapsulation efficiency (%)** | |
| --- | --- | --- | --- | --- | --- | --- |
|  | **4°C** | **25°C** | **4°C** | **25°C** | **4°C** | **25°C** |
| **0** | 221± 0.02 | 221 ± 0.02 | -21 ± 0.01 | -21 ± 0.01 | 85 ± 0.8 | 85 ± 0.8 |
| **7** | 218 ± 0.63 | 210 ± 0.84 | -20 ± 0.25 | -19 ± 0.24 | 83 ± 0.19 | 81 ± 0.14 |
| **15** | 212 ± 0.08 | 205 ± 0.03 | -19 ± 0.85 | -18 ± 0.45 | 80 ± 0.64 | 78 ± 0.32 |
| **30** | 200 ± 0.15 | 192 ± 0.69 | -17 ± 0.07 | -15 ± 0.36 | 79 ± 0.21 | 73 ± 0.04 |
| **60** | 193 ± 0.36 | 180 ± 0.21 | -16 ± 0.31 | -11 ± 0.7 | 72 ± 0.06 | 68 ± 0.51 |

*All samples were in triplicate

# Table S1. Storage stability study evaluating changes in hydrodynamic size (nm), Zeta potential (mV), and encapsulation efficiency under storage conditions.

| **Parameters** | **Groups** | |
| --- | --- | --- |
|  | **Control** | **Control**+ **βG@B-WPG NMs** |
| **Creatinine (mg/dl)** | 0.51 ± 0.3 | 0.61 ± 0.08 |
| **Blood urea nitrogen (BUN) (mg/dL)** | 16.56 ± 0.46 | 21.78± 0.51 |
| **Aspartate aminotransferase (AST) (IU/L)** | 41.22 ± 0.11 | 48.56 ± 3.03 |
| **Alanine aminotransferase (ALT) (IU/L)** | 58.78 ± 2.39 | 70.45 ± 2.64 |

# Table S2*.* Serum biomarkers for liver and kidney function were assessed to evaluate the safety profile of **Blank nanomicelles** (Control, and Control + **βG@B-WPG NMs**).

# Legends for Supporting video

## Supporting video file, SV 1: Assessment of physical activity in DSS-induced colitis mice (colitis model) and treated with βG@Apr-WPG NMs (DSS + βG@Apr-WPG NMs) (n = 3).

## Supporting video file, SV 2: Beam walk test to assess motor coordination in IBD mice treated with βG@Apr-WPG NMs for (A) Control (healthy), (B) DSS-induced colitis, (C) DSS + βG@Apr-WPG NMs (n = 3).

## **Supporting video file, SV 3:** **Grip strength test: assessing neuromuscular function in UC mice treated with βG@Apr-WPG NMs for** (A) Control (healthy), (B) DSS-induced colitis, (C) DSS + βG@Apr-WPG NMs (n = 3).

# Reference:

[1]	C. Jori, A. Ahmad, A. Kumar, B. Kumar, A. Ali, N. Ali, H. Tabassum, R. Khan, *Carbohydrate Polymers* **2025**, *359*, 123537.

[2]	R. K. Mishra, A. Ahmad, A. Kumar, A. Ali, null Kanika, C. Jori, S. Tabrez, T. A. Zughaibi, M. N. Almashjary, S. S. Raza, R. Khan, *Biomater Adv* **2023**, *148*, 213383.

[3]	Md. M. Ansari, A. Ahmad, R. K. Mishra, S. S. Raza, R. Khan, *ACS Biomater. Sci. Eng.* **2019**, *5*, 3380.

[4]	N. E. Toledano Furman, K. S. Prabhakara, S. Bedi, C. S. Cox Jr, S. D. Olson, *Cytometry Part A* **2018**, *93*, 182.
